# Genome-wide association study of prostate-specific antigen levels identifies novel loci independent of prostate cancer

**DOI:** 10.1038/ncomms14248

**Published:** 2017-01-31

**Authors:** Thomas J. Hoffmann, Michael N. Passarelli, Rebecca E. Graff, Nima C. Emami, Lori C. Sakoda, Eric Jorgenson, Laurel A. Habel, Jun Shan, Dilrini K. Ranatunga, Charles P. Quesenberry, Chun R. Chao, Nirupa R. Ghai, David Aaronson, Joseph Presti, Tobias Nordström, Zhaoming Wang, Sonja I. Berndt, Stephen J. Chanock, Jonathan D. Mosley, Robert J. Klein, Mridu Middha, Hans Lilja, Olle Melander, Mark N. Kvale, Pui-Yan Kwok, Catherine Schaefer, Neil Risch, Stephen K. Van Den Eeden, John S. Witte

**Affiliations:** 1 Department of Epidemiology and Biostatistics, University of California San Francisco, San Francisco, California 94158, USA; 2 Institute for Human Genetics, University of California San Francisco, San Francisco, California 94143, USA; 3 Division of Research, Kaiser Permanente, Northern California, Oakland, California 94612, USA; 4 Department of Research and Evaluation, Kaiser Permanente Southern California, Pasadena, California 91101, USA; 5 Department of Urology, Kaiser Oakland Medical Center, Northern California, Oakland, California 94612, USA; 6 Department of Medical Epidemiology and Biostatistics, Karolinska Institutet, Stockholm 17177, Sweden; 7 Laboratory of Translational Genomics, Division of Cancer Epidemiology and Genetics, Department of Health and Human Services, National Cancer Institute, National Institutes of Health, Bethesda, Maryland 20814, USA; 8 Department of Medicine, Vanderbilt University, Nashville, Tennessee 37232, USA; 9 Icahn Institute for Genomics and Multiscale Biology, Department of Genetics and Genomic Sciences, Icahn School of Medicine at Mount Sinai, New York, New York 10029 USA; 10 Departments of Laboratory Medicine, Surgery, and Medicine, Memorial Sloan-Kettering Cancer Center, New York, New York 10065, USA; 11 Nuffield Department of Surgical Sciences, University of Oxford, Oxford OX3 7LD, UK; 12 Department of Translational Medicine, Lund University, Malmö 205 02, Sweden; 13 Department of Clinical Sciences, Lund University, Malmö 205 02, Sweden; 14 Department of Urology, University of California San Francisco, San Francisco, California 94158, USA

## Abstract

Prostate-specific antigen (PSA) levels have been used for detection and surveillance of prostate cancer (PCa). However, factors other than PCa—such as genetics—can impact PSA. Here we present findings from a genome-wide association study (GWAS) of PSA in 28,503 Kaiser Permanente whites and 17,428 men from replication cohorts. We detect 40 genome-wide significant (*P*<5 × 10^−8^) single-nucleotide polymorphisms (SNPs): 19 novel, 15 previously identified for PSA (14 of which were also PCa-associated), and 6 previously identified for PCa only. Further analysis incorporating PCa cases suggests that at least half of the 40 SNPs are PSA-associated independent of PCa. The 40 SNPs explain 9.5% of PSA variation in non-Hispanic whites, and the remaining GWAS SNPs explain an additional 31.7%; this percentage is higher in younger men, supporting the genetic basis of PSA levels. These findings provide important information about genetic markers for PSA that may improve PCa screening, thereby reducing over-diagnosis and over-treatment.

## Abstract (web summary)

Prostate-specific antigen is used as a biomarker of prostate cancer, but levels can be affected by other factors not related to cancer. Here, the authors find genes associated with prostate specific antigen levels in healthy men, which could be used to reduce over-diagnosis and over-treatment.

Prostate specific antigen (PSA) is a blood-based biomarker used for the detection and surveillance of prostate cancer (PCa)^1^. PCa can cause disruption of the prostate's cellular architecture, which in turn can result in PSA leaking into circulating blood. However, PSA levels can also be affected by benign prostatic hyperplasia (BPH), local inflammation or infection, prostate volume, age^2^, and germline genetics. In this regard, PSA is an organ—but not cancer—specific biomarker.

PSA screening for PCa has been used for over 20 years, but its use has declined recently because of concerns about over-diagnosis and over-treatment^3^,^4^. While PSA levels at mid-life may modestly predict long-term PCa risk^5^, and high PSA levels are correlated with more aggressive and lethal forms of disease^6^,^7^,^8^, low PSA levels do not rule out PCa, and high PSA levels have a low predictive value for PCa^9^. In the Prostate, Lung, Colorectal, Ovarian (PLCO) Cancer Screening Trial, which had substantial crossover, there was no appreciable reduction in mortality directly related to PSA screening^10^. Another randomized trial, however, showed that PSA screening may reduce PCa mortality^11^. Between 20 and 60% of PSA-screened PCas are estimated to be over-diagnoses, and non-aggressive PSA-detected PCas are often treated with therapy that may involve substantial side effects^12^,^13^.

The value of PSA screening may be higher among individuals defined by particular characteristics, such as family history of PCa, ethnicity, age, and genetic factors. PSA is a glycoprotein enzyme encoded by kallikrein-3 (*KLK3*) on chromosome 19, but evidence from genetic association studies suggests that PSA levels are a complex polygenic trait, influenced by several different genes. Determining the genetic basis of PSA levels unrelated to cancer may help increase both the sensitivity and specificity of screening for PCa by adjusting PSA levels for constitutive germline genetics. Doing so could improve PSA screening performance. Clinicians could more accurately decide who should have a prostate biopsy, thereby reducing unnecessary procedures and their associated morbidities, as well as decreasing over-diagnosis^14^,^15^.

Twin studies estimate that 40–45% of the variation in PSA levels can be explained by inherited factors^16^,^17^. However, the single-nucleotide polymorphisms (SNPs) that have been identified thus far^14^,^18^,^19^,^20^,^21^,^22^,^23^,^24^ only explain a limited percentage of the variation in PSA levels (4.2% in 4,620 subjects from Iceland, and 11.8% in 454 subjects from the UK)^14^. In addition, several of the loci that harbor SNPs associated with PSA levels also harbor SNPs associated with PCa, making it complicated to disentangle genetic effects on PSA levels versus PCa. PSA level associations with PCa risk variants may reflect a number of factors, including: (1) true disease-related increases in PSA levels; (2) the use of PSA levels to restrict controls in case-control studies of PCa; and/or 3) non-cancer related PSA levels that prompt additional biopsy screening (Supplementary Fig. 1). One study reported^14^ that correcting PSA levels using four PSA-associated variants reclassifies 3.0% of individuals as needing biopsies and 3.0% as not needing biopsies. It did not, however, assess whether cases and controls were reclassified in the desired direction or look at clinical outcomes.

To discover more variants directly associated with PSA levels, here we undertake a large genome-wide association study (GWAS) of PSA. Our study includes a total of 45,931 men: 40,328 men from a large Kaiser Permanente (KP) cohort^25^,^26^ and 5,603 independent controls. We first search genome-wide for variants associated with PSA levels among KP non-Hispanic white non-cases (i.e., individuals who had not been diagnosed with PCa, N=28,503). Then we test our findings for replication in the remaining 17,428 men, comprised of 11,825 additional men in the KP cohort (non-cases of other race or ethnicity groups, and PCa cases using their PSA levels at least two years before cancer diagnosis) and 5,603 external replication non-cases. We further investigate which variants were exclusively associated with PSA levels and not with PCa in the KP cohort. Finally, we assess how much variation in PSA levels is explained by the replicated variants and how much can be explained by the remaining SNPs on the array.

## Results

### GWAS of PSA levels

Details of the discovery and replication samples are presented in Table 1. The discovery GWAS included 28,503 non-Hispanic white men from KP who had not been diagnosed with PCa at the time of the electronic health record (EHR) data extraction in 2012. The 17,428 replication samples included KP non-cases of Latino, East Asian, and African American backgrounds, KP non-Hispanic white PCa cases (using PSA measures from at least two years prior to diagnosis), and three independent studies of non-Hispanic white non-cases. Median PSA levels were similar across the discovery and replication populations, except for the KP PCa cases, who had higher PSA levels.

**Figure 1:**
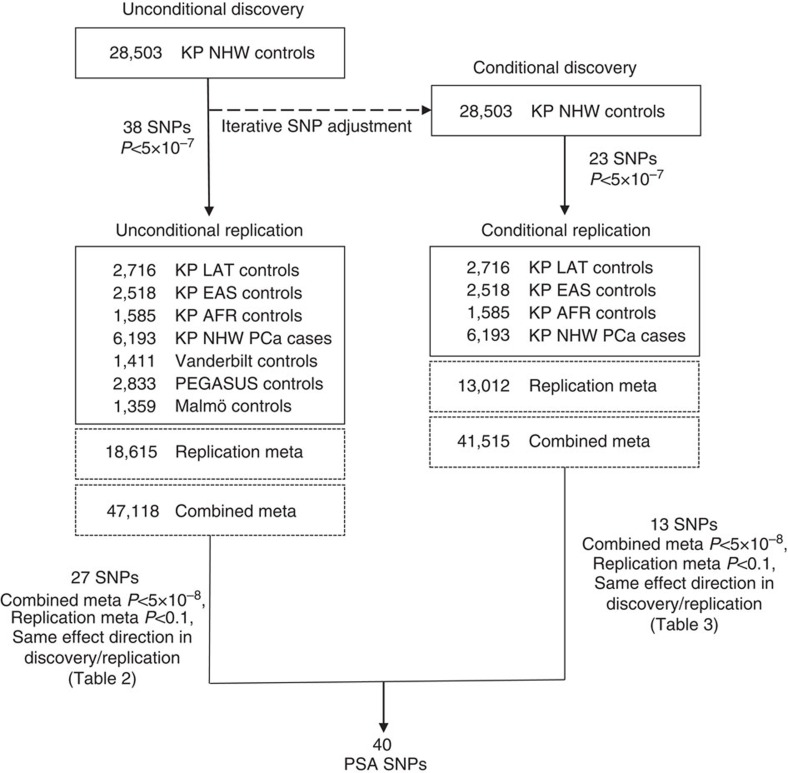
Flow chart highlighting the approach and samples used to detect SNPs associated with PSA from GWAS. First we undertook an unconditional discovery GWAS in the KP non-Hispanic white non-cases. Thirty-eight SNPs associated with PSA (*P*<5 × 10^−7^) were then included as covariates in a second conditional discovery GWAS. All SNPs associated with PSA (*P*<5 × 10^−7^) from these two GWAS (38+23) were evaluated for replication in two steps, first in an analysis of the replication cohorts alone, and then in a meta-analysis combining the discovery and replication cohorts. A total of 40 independent SNPs (27 in unconditional GWAS, 13 in conditional GWAS) met criteria of combined meta *P*<5 × 10^−8^, replication meta *P*<0.1, and same effect direction in discovery/replication.

Our approach to discovery and replication is outlined in Fig. 1. The primary criterion for inclusion of SNPs in the main tables of this manuscript was that the meta-analysis of discovery and replication cohorts met the conventional genome-wide significance threshold of *P*<5 × 10^−8^. To be considered for the meta-analysis, SNPs also had to have *P*<5 × 10^−7^ in the discovery cohort, *P*<0.10 in the replication, and the same effect estimate direction in the replication as discovery. All PSA association analysis and tests were undertaken with linear regression, adjusting for age and ancestry (see Methods section).

The primary discovery analysis entailed an unconditional GWAS in KP non-Hispanic white non-cases. In a secondary discovery step to identify additional independently associated SNPs, we performed a conditional GWAS, also in KP non-Hispanic white non-cases, in which we conditioned on the lead SNPs at each locus which had *P*<5 × 10^−7^ from the primary discovery analysis. We then tested all unconditional and conditional hits with *P*<5 × 10^−7^ for replication. While this significance level is slightly more liberal than the typical genome-wide significance threshold of 5 × 10^−8^, it allowed us to retain more potential SNPs to test in the replication cohort, and to test for genome-wide significance in the meta-analysis of discovery and replication. We tested the unconditional hits in all available replication cohorts and the conditional hits in the KP replication cohorts only (that is, the cohorts for which we had individual level data).

**Figure 2:**
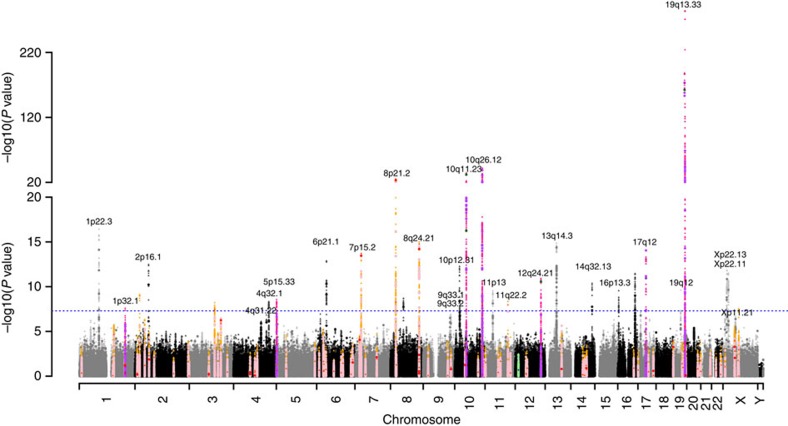
Results from a GWAS of PSA levels in 28,503 Kaiser Permanente non-Hispanic white non-cases. *P* values are for variant associations with log-transformed PSA levels, adjusted for age and ancestry principal components using a linear regression model. Black and grey peaks indicate novel findings. Dark purple and magenta indicates previously reported PSA level associated genotyped and imputed hits, respectively, and light purple and magenta indicate those within 0.5 Mb of previously reported hits that were replicated at genome-wide significance. Dark pink and red points denote previously reported PCa SNPs genotyped and imputed, respectively, and pink and orange indicate those within 0.5 Mb of previously reported PCa SNPs genotyped and imputed. Dark blue and green points denote the previously reported genotyped and imputed, respectively, SNPs associated with PSA levels only (and not PCa), and light blue and green those within 0.5 Mb previously reported hits. Circles denoted genotyped SNPs and triangles represent imputed SNPs.

Our unconditional GWAS yielded 38 lead SNPs (i.e., at each ±1 Mb locus) independently associated with PSA levels with *P*<5 × 10^−7^. These findings are highlighted in Fig. 2 (Manhattan plot). The genomic inflation factor for the GWAS was 1.06, indicating that our findings were not due to systematic bias that could reflect differences in population substructure (Q–Q plot, Supplementary Fig. 2).

In genome-wide conditional analyses, we detected 23 additional SNPs independently associated with PSA levels with *P*<5 × 10^−7^. The first round of these analyses conditioned on the 38 lead SNPs discovered in the unconditional GWAS. Models in subsequent rounds included the 38 original lead SNPs and all SNPs identified with *P*<5 × 10^−7^ in the previous round(s) of analysis. The conditional variants detected at new loci generally had *P* values just above 5 × 10^−7^ in the unconditional analysis. The decrease in *P* values after conditioning on other SNPs may have resulted from accounting for additional phenotypic variance.

The unconditional and conditional GWAS yielded 61 total SNPs (with *P*<5 × 10^−7^) for replication analysis. Of the 38 lead hits, 27 met the following three criteria and are displayed in Table 2 (more details in Supplementary Dataset 1): *P*<0.1 in the meta-analysis of all replication cohorts, the same effect direction in discovery and replication, and genome-wide significant (*P<*5 × 10^−8^) in the combined meta-analysis of the discovery and replication samples. Of the 23 conditional hits detected in discovery, 13 met the same three criteria (Table 3; more details in Supplementary Tables 1–3, including unconditional results).

Thus, in total, we detected 40 independent PSA hits meeting our criteria: 27 from unconditional and 13 from conditional analyses (Tables 2 and 3, each locus in Supplementary Fig. 3). Of the 40 SNPs, 19 were at 17 novel loci for both PSA levels and PCa, where a novel locus is defined as being located at least 1 Mb away from and not correlated with (*r*^2^<0.3) previous GWAS-identified variants (Tables 2 and 3). Six of the 40 SNPs were at four loci previously associated with PCa only, one was at a location previously associated with PSA only, and 14 occurred at 7 loci previously associated with both PCa and PSA^14^,^20^,^21^,^23^,^24^,^26^.

Ten PSA variants from unconditional analyses were either the same SNP—or correlated (*r*^2^>0.3) with a SNP—previously reported to be associated with PSA levels and/or PCa: rs4951018 (*SLC45A3*), rs37004 (*TERT-CLPTM1L*), rs10993994 (*MSMB*), rs12285347 (*MMP7*), and rs11263761 (*HNF1B*), as well as rs266849, rs266868, rs17632542, rs11665748 and rs6070 (*KLK3*–*KLK2*).

Six variants were novel for PSA levels, but were located at loci previously reported to be associated with PCa (pink in Fig. 2; Table 2, Table 3). These include: intronic SNP rs1991431 of *ZBTB38* on chromosome 3 (meta-analysis *P*=2.5 × 10^−11^); intronic SNP rs10486567 of *JAZF1* on chromosome 7 (*P*=4.3 × 10^−19^); rs4614003 near *SLC25A37* (*P*=1.0 × 10^−15^) and rs13272392 near *NKX3-1* (*P*=3.5 × 10^−34^), both on chromosome 8p; rs17464492 (*P*=1.5 × 10^−10^) and rs10505477 (*P*=6.5 × 10^−21^) at the chromosome 8q24 cancer-risk locus.

At 10q26.12 we detected a novel PSA association with rs10886902 in *FGFR2* (SNP previously reported associated with PCa), and subsequent conditional iterations identified nearby independent associations for intergenic SNP rs200367988 near *WDR11* and rs10749415 near *7SK* (Tables 2 and 3, and Supplementary Tables 1–3). These 3 SNPs comprised 6 common haplotypes (with frequency ≥0.01), all of which were associated with PSA levels (*P*<5 × 10^−4^; Supplementary Tables 4–5).

For *KLK3-KLK2*, we detected an extremely strong primary associated lead SNP (rs17632542, previously reported), and six other independently significant SNPs: rs266849, rs266868, rs11665748, rs61752561, rs2739472 and rs6070 (Tables 2 and 3, and Supplementary Tables 1–3; region in Supplementary Fig. 4). The locus is very complex; there were 18 common haplotypes with frequency ≥0.01, most of which were associated with PSA levels (Supplementary Tables 6–7).

Taken together, our results replicated almost all 17 previously reported PSA level SNPs at a strict Bonferroni correction of *P*<0.003 in the meta-analysis of all KP race or ethnicity groups (Supplementary Dataset 2)^14^,^20^,^21^,^23^,^24^. These included previously reported SNPs near *SLC45A3* and *SLC41A1* on 1q32.1, originally discovered in Chinese^21^, Japanese^24^ and Korean^20^ populations, and also replicated in our study most strongly in KP East Asian non-cases. The only exception was rs6679073 (ref. 20).

### Risk variants for PSA versus PCa

**Figure 3:**
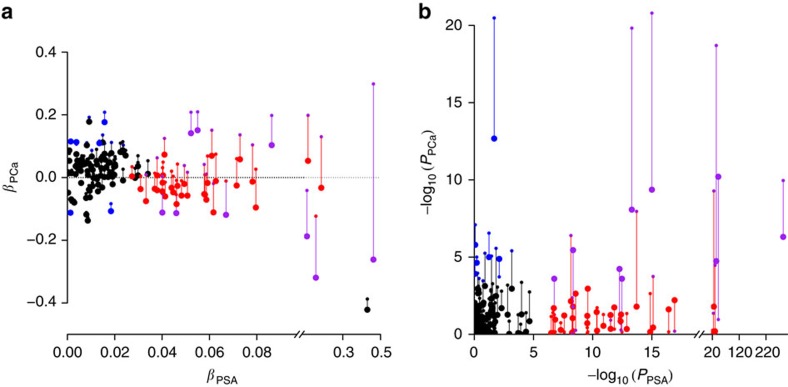
Comparison of the effect of SNPs on PSA levels versus on prostate cancer (PCa) in KP non-Hispanic whites. Results are from separate models regressing PSA level (linear regression) or PCa (logistic regression, unadjusted for PSA levels are small circles, adjusted for PSA levels are large circles) on each of the 40 PSA level SNPs from Tables 2 and 3 and the 105 previously identified PCa SNPs. (**a**) Effect sizes. (**b**) *P* values. Red points are associated with PSA levels only (from Tables 2 and 3), blue with PCa only (*P*<0.00125, a Bonferroni correction for 40 SNPs), purple with both PSA and PCa (for small circles, this also includes previously identified PCa associated SNPs), and black are previously identified PCa associated SNPs (not significant here). Previously reported PCa SNPs within 0.5Mb of a PSA SNP were removed: rs1775148, rs6763931, rs2242652, rs10486567, rs2928679, rs1512268, rs12543663, rs10086908, rs1016343, rs13252298, rs6983561, rs116041037, rs445114, rs16902104, rs6983267, rs7000448, rs11986220, rs10993994, rs2252004, rs11568818, 12:114685571, rs11649743, rs7501939 and rs2735839.

Twenty of the 40 hits for PSA levels reported here were previously associated with PCa or are located near known PCa loci (within 1 Mb and *r*^2^>0.3). Since PCa can have an impact on PSA levels, these overlapping results may reflect latent, undiagnosed disease. Alternatively, some of these PCa findings may be an artifact of screening or non-case sampling based on PSA levels. To differentiate between these possibilities, we analysed the association between PCa and the 40 PSA level SNPs reported here, with and without adjustment for PSA levels. This analysis was undertaken among the 4,999 non-Hispanic white cases that had PSA level measurements, and the 28,520 non-cases with PSA level measurements (Fig. 3, which also includes previously reported PCa SNPs; Supplementary Table 8).

In analyses that did not adjust for PSA levels, nine SNPs were associated with PCa at *P*<0.00125 (Bonferroni correction for the 40 SNPs). Eight of these were previously associated with PCa, and 1 was at a locus (but not SNP) associated with PCa. Seven additional SNPs were suggestively associated with PCa at 1.25 × 10^−3^≤*P*<0.05: 1 previously identified PCa SNP; 1 previously identified PCa locus; and 5 at novel loci. SNPs at the five novel loci were: rs6920449, rs8023057, and rs10855058 (near *RRAGB* at Xp11.21, a Ras-related GTPase^27^), rs202346 (*DLEU1* at 13q24, which is in a region often deleted in chronic lymphocytic leukemia^28^), and rs1991431 (*ZBTB38*). The remaining 24 of 40 PSA level SNPs were not associated with PCa.

When adjusting for PSA levels—coding genotypes to the PSA level increasing allele—the magnitudes of PCa associations were attenuated (Fig. 3). This is expected since PSA screening is used to diagnose PCa. Ten of the 40 PSA SNPs were Bonferroni significant for PCa at *P*<0.00125: 6 were known PCa SNPs (4 of which had *P*<0.00125 in the unadjusted model, 2 had *P*<0.05), and 4 were novel (rs2556375, rs6478343, rs11694038, and rs11084596). Ten additional SNPs were suggestively associated with PCa at 1.25 × 10^−3^≤*P*<0.05: three were at known PCa SNPs (all of these had *P*<0.00125 in the unadjusted), three were at known PCa loci (these had *P*<0.05 in the unadjusted), and four were novel for PCa (rs6662386, rs10023685, rs16980679 and rs5969745; all four had unadjusted *P*>0.05). Of particular interest are SNPs for which the PCa association *P* value decreased when adjusting for PSA; these SNPs are typically those with an antagonistic relationship between PSA levels and PCa (that is, where the allele increasing PSA level is associated with a decrease in PCa risk). The most extreme such example is rs10886902, which was initially not associated with PCa (*P*=0.11), but was strongly associated PCa after adjusting for PSA (*P*=6.2 × 10^−11^). This SNP was previously reported for PCa (described below). Two other neighboring SNPs showed similar effects, and haplotype results for this locus are given in Supplementary Tables 6 and 7.

The unconditional PSA level GWAS SNPs that were within 1 Mb of previously identified PCa SNPs tended to be very highly correlated with, if not the exact same as, the PCa SNP. The SNPs rs10486567 and rs10993994 were the same as previously identified with PCa. Our SNP rs13272391 has an *r*^2^=0.99 (1000 Genomes European ancestry) with the previously identified PCa SNP rs1512268, and we see similarly high correlations for rs10505477 (*r*^2^=0.92 with rs6983267), rs12285347 (*r*^2^=0.92 with rs11568818), rs10886902 (*r*^2^=0.84 with rs11199874), and rs11263761 (*r*^2^=0.98 with rs4430796). Three exceptions are rs1774148, which has a weaker *r*^2^=0.22 with the previously identified PCa SNP rs1775148; rs59482735, which is 784 Kb from the previously identified PCa SNP rs1571801 and has *r*^2^=0.0015; and rs11067228, which is 409 Kb from the previously identified PCa SNP rs1270884 and has *r*^2^=0.00015. All three are most likely independent SNP associations.

### Variation of PSA levels explained by genetics

We investigated how much variation in PSA levels was explained by genetics with three different analyses: heritability using all subjects; familial correlations among the related individuals in the KP cohort; and polygenic risk scores. For the heritability analyses, we partitioned the genome into the 40 PSA SNPs, and the remainder of the genotyped and imputed SNPs. We calculated PSA heritability for these two partitions in the KP non-Hispanic white non-cases. Using a joint variance components analysis of the 40 SNPs and the rest of the genome (see Methods section), we estimated that the 40 SNPs explained ∼9.5% (s.e.=2.0%) of the variability in PSA levels, of which an estimated 38.9% was from the *KLK3* region. The remainder of the genotyped and imputed SNPs explained an additional 31.7% (s.e.=2.7%).

We next calculated the intra-class correlation (ICC) between 200 non-case sibling pairs (average age 56.0 years) in the KP non-Hispanic whites. (Note that one member of each sibling pair was excluded from the GWAS) The overall ICC was 26.2% (95% confidence interval (CI)=12.8–38.7%), leading to an upper estimate of heritability of 52.4% (95% CI=25.6–77.4%). The estimates differed by age, although the confidence intervals overlapped: for ≤54 years was ICC=33.5% (95% CI=14.6–50.1%) leading to *h*^2^=67.0% (95% CI=29.1–100%), and for >54 years was ICC=−1.1% (95% CI=−20.4–18.2%) leading to *h*^2^=0% (95% CI=0–36.5%). For 178 father-son pairs (average father age 66.7y, average son age 47.2 years), the estimated Pearson correlation was 9.7% (95% CI=−5.0–24.0%), leading to an upper estimate of heritability of 19.4% (95% CI=0–48.0%).

We used a polygenic risk score to compare the variance explained by—and the effect sizes of—the 40 PSA level SNPs by KP race or ethnicity groups and also by age. As expected, the risk score was highly significant in all four groups (Table 4). The 95% confidence intervals of most of the effect sizes overlapped, although the variance explained was lower in African Americans. This reflects that frequencies of the risk SNPs are generally lower in African Americans, which could be the result of ascertainment bias (given that the discovery cohort was non-Hispanic white). Of note, the variance explained in the non-Hispanic whites was higher at earlier ages (Table 4), consistent with the correlation patterns in first degree relatives. While effect sizes were comparable across age, the variance of PSA levels increases with age, likely due to a variety of factors such as BPH (Table 4).

## Discussion

Our GWAS detected 40 independent SNPs associated with PSA levels that explained 9.5% of the inter-individual variation. Many of the novel SNPs were associated with PSA and not PCa, although some were associated with PCa even after adjustment for PSA.

Our unconditional and conditional GWAS identified seven common independent SNPs at *KLK3-KLK2* (Supplementary Fig. 3; Supplementary Dataset 2, previously identified PSA SNPs). In *KLK3* exon 3, we identified a 3% frequency missense variant, rs61752561 (Asp102Asn), which has had inconsistent candidate gene results^29^,^30^,^31^,^32^. The SNP was uncorrelated with the two previously reported genome-wide significant PSA SNPs at the locus^14^ (1000 Genomes European ancestry rs266849 *r*^2^=0.006; rs17632542 *r*^2^=0.002). However, three of the SNPs we identified near *KLK3* (rs266868, rs11665748, and rs6070) are correlated with previously reported PSA level SNPs (rs2659051 (ref. 20), rs266870 (ref. 14) and rs1354774 (ref. 23)). Only rs266849, rs11665748 and rs17632542, which were strongly associated with PCa risk in our study (*P*<5 × 10^−8^), appear to be correlated with previous GWAS-identified PCa susceptibility polymorphisms of this locus^18^,^33^,^34^,^35^,^36^. These variants are near the *KLK3* promoter, which harbors several androgen-responsive elements^37^,^38^.

Outside of *KLK3-KLK2*, rs116940348, a 3% frequency variant near COMMD3-BMI1-SPAG6, had the largest estimated PSA effect size (16%, 95% CI=11%–20%). *COMMD3* plays a role in the NF-κB pathway^39^ and is often involved in protein-fusion products with *BMI1*, a polycomb ring finger oncogene overexpressed in PCa^40^. The neighboring gene, *SPAG6,* is a sperm-associated antigen^41^. Other members of the sperm-associated antigen family have been proposed as biomarkers for urological and hormonal cancers^42^,^43^,^44^. Furthermore, the rs116940348 allele associated with increased PSA levels in men without PCa was associated with a decreased PCa risk. Similarly, the minor allele of rs10886902 (*FGFR2*) was associated with an 11% increase (95% CI=9–12%) in PSA levels, but a significantly lower risk of PCa (OR=0.90, *P*<0.00125), consistent with a previous report for a correlated variant (rs11199874 *r*^2^=0.83)^45^. Taken together, these results are particularly intriguing because an understanding of biological mechanisms by which genetically elevated PSA can occur in the absence of increased risk of PCa may help improve PSA specificity.

Five SNPs in or near *SLC45A3* (prostein) at chr 1q32.1 have been previously associated with PSA levels in Asian populations (rs12409639, rs16856139, rs823123, rs6679073 and rs2153904)^20^,^21^,^24^. All five had *P*<0.02 in KP East Asian non-cases, but only rs2153904 replicated in KP non-Hispanic white non-cases (*P*=3.4 × 10^−3^; Supplementary Dataset 2). We identified an additional genome-wide significant intronic SNP of *SLC45A3* (rs4951018) that was weakly correlated with rs2153904 in Asians (*r*^2^=0.36), but not in those of European ancestry (*r*^2^=0.04). These variants were not associated with PCa after PSA adjustment.

Many of the novel SNPs we found to be associated with PSA levels are intronic, including SNPs in genes in pathways involved in cellular signalling, growth, and differentiation (*PHF19*, *TXLNG*, *RAI2*)^46^,^47^,^48^, and in the development of hematologic malignancies (*BCLL1A* and *DLEU1*)^49^,^50^. SNPs correlated with rs202346 near *DLEU1* have also been found to be associated with anthropomorphic traits^51^,^52^,^53^,^54^,^55^. Although intergenic, the 16p13.3 SNP near *transcription factor AP-4* (ref. 56) (*TFAP4*; rs9921192) is in a LD block with a missense variant in exon 5 of this gene (Gln158His; rs251732). *TFAP4* activates both viral and cellular genes^56^. Of note, rs56935123 15 kb 5' from *ZNF827* and rs59482735 4 kb 5' from *PHF19* are insertion-deletions, variant types that have not been commonly examined in previous GWAS of PSA levels.

SNP rs37004 of *TERT-CLPTM1L* locus is correlated with known PSA variant rs401681 (ref. 14) (*r*^2^=0.30), and with variants associated with multiple cancers including testicular, bladder, lung, skin, blood, and pancreatic^57^,^58^,^59^,^60^,^61^,^62^,^63^,^64^,^65^,^66^. It is not, however, correlated with the nearby PCa susceptibility variant rs2242652 (ref. 67) (*r*^2^<0.02 in European, Latino, Asian and African ancestry).

For SNPs that did not replicate, several issues may have reduced our power. First, our replication cohorts were smaller than the original sample, especially in the conditional analysis (which used only the other KP groups). Second, winner's curse^68^ stipulates that discovery estimates are generally stronger than the truth. Third, there may have been heterogeneity among the groups for a variety of reasons, including allele frequency differences, LD differences, race or ethnicity differences, and that the PCa cases tended to have weaker effects. Six of the 27 unconditional (22.2%), and 3 of the 13 conditional (23.1%) SNPs had nominally significant (*P*<0.05) heterogeneity, much more than the 5% expected by chance (Supplementary Dataset 1, Supplementary Tables 1–3). Thus the failure of replication of some of the variants may have been due to low power, and potential heterogeneity among the different groups.

Distinguishing whether SNPs are associated with PSA, PCa or both, is challenging. PCa impacts PSA levels, but so do a number of other factors (for example, benign age related growth, infection, inflammation, genetics). In addition, the use of PSA levels in both screening for PCa and for restriction of controls to men with low PSA levels in case-control association studies may induce apparent associations with PCa^14^. Nevertheless, we tried to address these issues by adjusting our PCa analysis for PSA. Interestingly, we found a number of SNPs associated with both phenotypes. In addition, the SNPs we found within 1 Mb of previously identified PCa SNPs were generally the same or very highly correlated with the PCa SNP, indicating they were often the same signal. Of the 40 PSA level SNPs reported here, 10 were also associated with PCa at a Bonferroni significance level after adjusting for PSA, and an additional 10 were nominal. It may be that genes that impact only PSA levels are associated with distinct biological processes from genes that impact both PSA and PCa. For example, some genes associated with PSA and PCa exhibit carcinogenic properties; *HNF1B* and *NKX3-1* encode transcription factors expressed in prostate adenocarcinoma that may be androgen sensitive^69^. In contrast, some PSA level-only SNPs may be linked with excessive transportation of PSA into circulation independent of malignancy-associated leakage. For example, variants of genes such as *SERPINA3*, which encodes α1-antichymotrypsin, a protease inhibitor frequently found complexed with PSA in circulation, may alter physical attributes of PSA^70^.

The existence of SNPs that influence PSA levels but not PCa while others appear to influence both highlights the complexity of using conventional PSA levels as a screening tool for PCa. Since some PSA level SNPs appear to have no impact on PCa, elevated PSA level alone is not necessarily a risk factor for PCa. Nevertheless, the SNPs associated with both PSA level and PCa indicate some overlapping biological mechanisms for PSA and PCa. Determining the extent to which the mechanisms overlap is complicated by the varying magnitude of associations of these SNPs on the two traits.

For SNPs associated with both PSA and PCa, the estimated effects on PCa may have been previously overestimated due to their impact on PSA levels^71^. This overestimation likely ranges from 2 to 10% depending on the strength of associations with PCa and PSA level^71^. The largest bias occurs when the true association between a SNP and PCa is null, but the SNP has a strong effect on PSA level^71^. Then, previously identified GWAS PCa hits may be due to the impact of PSA level on the detection of asymptomatic PCa. The converse, however, is not likely true- since most of the previously reported PCa SNPs are not associated with PSA levels, the PSA level SNPs reported here are unlikely due to indolent PCa in some controls.

In a heritability analysis, we observed that the 40 SNPs reported here explain ∼9.5% of the total variability in PSA levels, and the remainder of the GWAS array explains another 31.7%. Thus, while the significantly associated SNPs explain a noteworthy proportion of PSA level heritability, much more is ‘hidden' and should be detectable with larger sample sizes. Our estimate of heritability from our siblings depended on age (*h*^2^=52.4% overall, *h*^2^=67.0% for ≤54 years, and *h*^2^=0.0% for >54 years), although the confidence intervals overlapped, and our estimate from father offspring was *h*^2^=19.4%, likely also influenced by age of father and offspring. The estimates are somewhat comparable to previous family-based studies, which estimated heritability to be 44.8% for average age 52 in *n*=84 twins^16^ and 39.6% for average age 43 in *n*=2,604 men in pedigrees including roughly half sibling and half father-offspring^17^. These estimates indicate that a good amount of PSA heritability can be explained by our GWAS array. Additional parts of the missing heritability may be from rare variants, as has been suggested for prostate cancer^72^.

The additional PSA-associated SNPs discovered in this GWAS could be used to help normalize each man's PSA level by the amount by which his SNPs may have increased his PSA level, as has been illustrated before^14^. This can re-classify individuals not requiring biopsies to not having a biopsy ordered, and individuals who should be biopsied to having a biopsy ordered. Then it would be important to evaluate the clinical impact of re-classified PSA values on prostate cancer mortality.

In summary, the current study identifies a number of new loci associated with PSA levels using longitudinal EHR-derived measurements. We also provide evidence that further work will detect additional genetic markers. The discovered PSA SNPs may increase our ability to classify individuals who should and should not be biopsied, which could reduce over-treatment and over-diagnosis.

## Methods

### Participants and phenotypes

We undertook a discovery GWAS of PSA levels in 28,503 non-Hispanic white non-cases from KP, and tested for replication in 17,428 additional samples from KP, PEGASUS, Malmö (Sweden), and Vanderbilt (Table 1).

The KP samples included a total of 40,328 men from the Research Program on Genes, Environment and Health (RPGEH) Genetic Epidemiology Research on Adult Health and Aging (GERA) cohort, the ProHealth Study, and the California Men's Health Study, as previously described^26^. In addition to the 28,503 non-Hispanic white non-cases used for GWAS discovery, the KP replication samples included other race or ethnic non-cases (2,716 Latinos, 2,518 East Asians and 1,585 African Americans) and 5,006 non-Hispanic white PCa cases (using pre-diagnostic PSA levels, described below).

In the KP cohort, PCa status was determined from the Kaiser Permanente Northern California Cancer Registry (KPNCCR), the Kaiser Permanente Southern California Cancer Registry (KPSCCR) or through review of clinical electronic health records (EHR) through the end of 2012 (ref. 26). PSA levels were abstracted from KP EHR from 1981 through 2015. The KP discovery and replication non-cases had a total of 252,744 PSA measurements—on average nine PSA measures per subject. We used all of these repeated PSA measures in our analyses (described below). The KP replication cases were restricted to those men with a PSA measurement at least two years prior to PCa diagnosis, and analyses included only the earliest recorded PSA level so as to capture PSA before any PCa influence. The median time between the cases' oldest PSA level and PCa diagnosis was 8.6 years (MAD=4.8, distribution roughly normally distributed, but truncated at 2), indicating that these PSA levels sufficiently predated the PCa diagnosis to be useful for replication.

The three non-KP replication samples included 2,833 non-Hispanic white non-cases from the PEGASUS study^34^; 1,359 white non-cases from the Malmö Diet and Cancer (MDC) study^30^; and 1,411 white subjects from Vanderbilt University's BioVU^73^. For PEGASUS, non-cases between ages 55–74 years were selected between 1993 and 2001 from non-Hispanic white men in the Prostate, Lung, Colorectal, and Ovarian (PLCO) Cancer Screening Trial, with the Prostate portion of the trial formed to test if screening men with digital rectal examination plus PSA would reduce mortality from PCa, as described previously^74^, and the first screening PSA measure was used. For the MDC, male participants who were not diagnosed with prostate cancer as of December 2014 and for whom genome-wide SNP genotyping and PSA level measurements were available were included. The PSA level measures were conducted in a research setting in a subset of the participants in the MDC as previously described^30^. For BioVU, PSA measurements were collected as part of each subject's routine clinical care, and men with PSA>10 were excluded; for men with multiple PSA measurements, the median PSA was used. The Kaiser Permanente Northern California Institutional Review Board and the University of California San Francisco Human Research Protection Program Committee on Human Research approved the KP study. The institutional review board at each centre and the National Cancer Institute approved the PLCO study. The local ethics committee approved the MDC study. The Vanderbilt Institutional Review Board approved the BioVU study. Written informed consent was obtained from all subjects.

### Genotyping and imputation

All men from the KP cohort were genotyped for over 650,000 SNPs on four race or ethnicity-specific Affymetrix Axiom arrays optimized for individuals of non-Hispanic white, Latino, East Asian, and African-American race or ethnicity respectively^75^,^76^. Genotype quality control (QC) procedures and imputation for the original GERA cohort assays were performed on an array-wise basis, as has been described previously^26^,^75^,^77^. Briefly, imputation was done by pre-phasing KP genotypes with SHAPEIT v2.5 (ref. 78), and then imputing variants from the 1000 Genomes Project October 2014 release with 2,504 samples (http://1000genomes.org) as a cosmopolitan reference panel with IMPUTE2 v2.3.1 (ref. 79). Our discovery GWAS analysis in the non-Hispanic white non-cases ultimately assessed 10,109,774 variants with *r*^2^_info_≥0.3 and MAF≥0.01.

Genotyping, imputation and QC were similar in the three non-KP replication cohorts as has been previously described. Briefly, PEGASUS men were genotyped with the Illumina HumanOmni2.5 Beadchip^34^; Malmö men were genotyped on the Illumina Human OmniExpressExome v1.0 BeadChip; and BioVU men were genotyped on the Illumina Human660W-Quadv1_A, HumanOmin1-Quad, and HumanOmni5-Quad^73^. All studies were pre-phased and imputed with the same software as the GERA cohort, except Malmö used SHAPEIT v2r790 and IMPUTE2 v2.3.0.

### GWAS analysis and replication

To account for the uncertainty of genotype imputation, we modelled each SNP in our GWAS using additive dosages, which has been shown to work well^80^. We initially modelled log(PSA), transformed to be more normally distributed, with linear mixed models (to account for the repeated measures) adjusting for age at each PSA level measurement and ancestry covariates, and computed the residuals from the model. This is nearly identical to a long-term average^81^, except it uses the median instead of the mean (to better handle any potential outlier PSA level values). We repeated the analysis using the mean instead of median, and the results did not change materially. The top ten principal components from Eigenstrat v4.2 (ref. 82), as has been previously described^26^, were included in the linear model as ancestry covariates. Each SNP was then tested for association in a linear regression with each man's median residual from the models. We retained the lead SNPs in this unconditional GWAS analysis using a ±1 Mb window.

Following the unconditional GWAS, we sought to identify additional independently associated SNPs by repeating the GWAS analysis but conditioning on the observed unconditionally suggestive results (*P*<5 × 10^−7^). We elected to use a more liberal *P*<5 × 10^−7^ to test replication with a larger number of SNPs, recognizing that a smaller percentage of those with only marginal genome-wide significance would end up with evidence of replication. We created groups of SNPs with *P*<5 × 10^−7^ in the unconditional discovery GWAS that were within 1 Mb of any other SNP with *P*<5 × 10^−7^. We then chose the most significant lead SNP in each of these groups, and reran the full genome-wide analysis in the non-Hispanic whites, adjusting for these lead SNPs, along with age and ancestry covariates, to find additional independent PSA SNPs with *P*<5 × 10^−7^. We iterated this process until no additional SNPs were found, which required a total of six rounds of conditional analyses.

We tested SNPs with *P*<5 × 10^−7^ identified from unconditional analyses for replication in all replication cohorts. We assessed replication for SNPs with *P*<5 × 10^−7^ identified from conditional analyses only in the KP replication samples (since only these samples had full individual-level data available for conditioning). We tested for replication in two steps. First, we performed a meta-analysis of replication cohorts, testing the previously retained SNPs for association using the same additive linear model. All SNPs with a replication association *P*<0.1 and with an effect in the same direction as in the discovery analysis were retained. Second, these retained SNPs were tested for association in the meta-analysis of the discovery and replication cohorts combined. All genome-wide significant SNPs (*P*<5 × 10^−8^) in this meta-analysis are presented in the main tables. All replication analyses adjusted for age at PSA test and ancestry covariates (using principal components); BioVU additionally adjusted for body mass index. We combined results across studies using fixed effects meta-analysis.

### Analysis of PSA and PCa SNPs

For the previously reported and newly identified PSA-associated SNPs, we conducted a logistic regression analysis of PCa using the KP non-Hispanic white cases and non-cases, adjusting for age, body mass index and ancestry PCs (as described for PSA levels), to investigate whether the PSA level-associated SNPs were also associated with PCa risk. These analyses were performed twice, once adjusting for PSA levels, and once without adjustment, to determine whether the SNP effect on PCa could be fully explained by or confounded by its effect on PSA levels. We then compared the SNP-specific *P* values and effect estimates for PSA levels and PCa, with respect to magnitude and direction.

### GWAS array heritability

We estimated the narrow sense (additive) heritability of PSA levels explained by the associated SNPs and by the remainder of the SNPs on the Affymetrix Axiom array using a joint variance components fit in Genome-wide Complex Trait Analysis (GCTA)^83^. Array heritability estimates can be more sensitive to artifacts than GWAS results^83^, so we limited this analysis to 26,993 non-Hispanic white non-cases genotyped with the Axiom v1.0 Reagent Kit from KP, and undertook a number of additional QC steps, as previously described^26^. We additionally removed individuals such that there were no pairwise relationships with estimated kinship >0.05 remaining in the sample, resulting in 23,445 non-cases at 402,748 autosomal genotyped SNPs (allele frequency filtered so that MAF≥0.01 and LD-filtered so no two SNPs had *r*^2^≥0.8) and 2,184,083 imputed SNPs (after filtering for *r*_info_^2^≥0.3, MAF≥0.01, and LD-filtered as described for the genotyped SNPs).

### Polygenic risk scores

We constructed a polygenic risk score for PSA by summing the additive coding of each SNP weighted by the estimated effect size, and then standardized the distribution of all groups simultaneously by the mean and standard deviation (that is, to a standard normal distribution) for interpretability purposes. We used the lead SNP from each locus.

### Data availability

To maintain individuals' privacy, data on the GERA cohort are available by application to the Kaiser Permanente Research Bank (researchbank.kaiserpermanente.org).

## Additional information

**How to cite this article:** Hoffmann, T. J. *et al*. Genome-wide association study of prostate-specific antigen levels identifies novel loci independent of prostate cancer. *Nat. Commun.* **8**, 14248 doi: 10.1038/ncomms14248 (2017).

**Publisher's note:** Springer Nature remains neutral with regard to jurisdictional claims in published maps and institutional affiliations.

## Supplementary Material

Supplementary Information Supplementary Figures and Supplementary Tables

Supplementary Dataset 1 SNPs associated with PSA levels in KP non-Hispanic white controls and replication studies.

Supplementary Dataset 2 Associations in KP for 22 SNPs previously-identified associated with PSA levels.

## Figures and Tables

**Table 1:** Sample size, age and PSA levels of subjects included in discovery GWAS and replication studies.

**Study population**	***N***	Mean age* **(s.d.)**	Median PSA* **(MAD)**
Discovery			
KP non-Hispanic White non-cases	28,503	68.8 (10.2)	1.5 (1.2)
			
Replication			
KP Latino non-cases	2,716	65.6 (11.2)	1.4 (1.2)
KP East Asian non-cases	2,518	66.0 (10.5)	1.5 (1.2)
KP African American non-cases	1,585	66.1 (9.3)	1.4 (1.2)
KP non-Hispanic White Cases	5,006	60.8 (7.6)	2.7 (2.2)
Vanderbilt White non-cases	1,411	59.7 (11.4)	0.8 (3.5)
PEGASUS White non-cases	2,833	63.3 (5.1)	1.1 (0.89)
Malmö White non-cases	1,359	61.4 (6.3)	1.0 (0.70)

GWAS, genome-wide association study; KP, Kaiser Permanente; MAD, median absolute deviation; PSA, prostate-specific antigen.

KP non-Hispanic white non-cases are included in the discovery GWAS. The other seven populations are included in the replication.

^*^For KP non-cases, age and median are of their most recent PSA measurement. For KP cases, age and median are of their oldest PSA measurement, required to be at least two years prior to diagnosis.

**Table 2:** Unconditional genome-wide association study results.

**Chrom**	**Position**	**Genes**	**SNP**	**PSA increasing allele/other allele**	**Kaiser Permanente NHW non-cases (*****n*****=28,503)**			**Replication meta-analysis (*****n*****=18,615)**		**Combined meta-analysis (*****n*****=47,118)**		
					**PSA Increasing Allele Frequency**	***β***† **(× 10**^**2**^**)**	***P*****-value**	***β***† **(× 10**^**2**^**)**	***P*****-value**	***β***† **(× 10**^**2**^**)**	***P*****-value**	**Previous PCa/PSA GWAS hits**‡
1p22.3	88,190,037	*LMO4*	rs6662386	C/T	0.44	5.8	3.8 × 10^−17^	3.8	1.1 × 10^−4^	5.2	7.2 × 10^−20^	N*/N
1q32.1	205,636,334	*SLC45A3*	rs4951018	C/A	0.22	4.8	2.5 × 10^−8^	2.4	0.035	3.9	1.0 × 10^−8^	N/S
2p16.1	60,759,747	*BCL11A*	rs2556375	G/T‡	0.17	6.7	3.4 × 10^−13^	6.2	3.3 × 10^−7^	6.5	6.2 × 10^−19^	N**/N
4q31.22	146,874,227	*ZNF827*	rs56935123	AT/A	0.45	3.8	1.5 × 10^−7^	2.9	7.3 × 10^−3^	3.5	4.4 × 10^−9^	N/N
4q32.1	157,534,249	*PDGFC*	rs10023685	G/C‡	0.37	4.1	4.6 × 10^−9^	2.1	0.034	3.5	1.5 × 10^−9^	N*/N
5p15.33	1,356,684	*TERT-CLPTM1L*	rs37004	C/T	0.79	6.2	2.8 × 10^−9^	5.8	6.6 × 10^−5^	6	8.9 × 10^−13^	L*/S
6p21.1	43,710,348	*VEGFA*	rs6920449	T/C	0.16	7.2	1.3 × 10^−13^	2.1	0.095	5.3	6.4 × 10^−12^	N/N
7p15.2	27,976,563	*JAZF1*	rs10486567	G/A	0.76	6.1	2.0 × 10^−14^	5.1	2.6 × 10^−6^	5.8	4.3 × 10^−19^	S*/N
8p21.2	23,528,511	*NKX3-1*	rs13272392	T/A	0.43	7.3	3.5 × 10^−26^	6	6.5 × 10^−10^	6.9	3.5 × 10^−34^	S*/N
8q24.21	128,407,443	8q24/*MYC*	rs10505477	A/G	0.49	5.5	9.9 × 10^−16^	4.8	6.3 × 10^−7^	5.3	6.5 × 10^−21^	S**/N
9q33.1	120,732,749	*TLR4*	rs6478343	C/T	0.82	4.6	1.8 × 10^−7^	3	0.03	4.2	2.4 × 10^−8^	N**/N
9q33.2	123,643,426	*PHF19*	rs59482735	T/TAA‡	0.32	4	4.9 × 10^−8^	2.8	0.014	3.6	3.8 × 10^−9^	N/N
10p12.31	22,581,581	*COMMD3-BMI1-SPAG6*	rs116940348	A/G	0.03	15.7	5.4 × 10^−13^	10.7	3.4 × 10^−3^	14.4	1.4 × 10^−14^	N**/N
10q11.23	51,549,496	*MSMB*	rs10993994	T/C	0.39	8.6	3.4 × 10^−35^	8.1	1.8 × 10^−17^	8.4	1.2 × 10^−50^	S**/S
10q26.12	123,049,264	*FGFR2*	rs10886902	C/T	0.24	11	8.1 × 10^−43^	8.3	3.7 × 10^−13^	10.1	1.5 × 10^−53^	S**/S
11p13	34,783,417	*EHF-APIP*	rs4378355	C/G	0.39	4.4	2.9 × 10^−10^	2.1	0.046	3.7	2.4 × 10^−10^	N/N
11q22.2	102,396,607	*MMP7*	rs12285347	C/T	0.46	4	4.5 × 10^−9^	3.5	5.0 × 10^−4^	3.8	8.2 × 10^−12^	S**/S
12q24.21	115,094,260	*TBX3*	rs11067228	A/G	0.54	4.7	1.2 × 10^−11^	3.8	1.4 × 10^−4^	4.4	1.3 × 10^−14^	N/N
13q14.3	51,087,443	*DLEU1*	rs202346	A/C	0.25	6.3	1.4 × 10^−15^	6	6.3 × 10^−8^	6.2	3.4 × 10^−22^	N/N
14q32.13	95,097,556	*SERPINA3*	rs8023057	A/G	0.82	5.9	4.3 × 10^−11^	6.4	6.3 × 10^−6^	6.1	1.6 × 10^−15^	N/N
16p13.3	4,349,111	*GLIS2-TFAP4*	rs9921192	C/T‡	0.5	4.5	2.8 × 10^−10^	1.7	0.086	3.5	1.0 × 10^−9^	N/N
17q12	36,097,775	*HNF1B*	rs11263761	A/G	0.51	5.2	4.9 × 10^−14^	5	2.6 × 10^−7^	5.2	5.6 × 10^−20^	S**/S
19q12	32,104,979	*THEG5*	rs11084596	T/C	0.61	4.6	2.6 × 10^−10^	5	6.6 × 10^−7^	4.8	9.6 × 10^−16^	N**/N
19q13.33	51,361,757	*KLK3*	rs17632542	T/C	0.92	46.2	7.3 × 10^−285^	35.3	2.1 × 10^−56^	43.5	2.4 × 10^−340^	S**/S
Xp22.13	16,741,044	*RAI2*	rs16980679	G/A	0.95	8	1.5 × 10^−12^	6.8	3.1 × 10^−4^	7.7	2.9 × 10^−15^	N*/N
Xp22.11	24,046,434	*EIF2S3-KLHL15*	rs6627995	T/C	0.33	3.7	3.1 × 10^−12^	2.6	5.0 × 10^−3^	3.4	9.7 × 10^−14^	N/N
Xp11.21	55,324,960	*RRAGB*	rs10855058	A/G	0.3	2.7	2.3 × 10^−7^	2.1	9.7 × 10^−3^	2.5	1.0 × 10^−8^	N/N

L, previously identified locus; N, novel locus; NHW, Non-Hispanic white; PCa, prostate cancer; PSA, prostate-specific antigen; S, previously identified SNP; SNP, single-nucleotide polymorphism.

SNPs included here have association *P* <5 × 10^−8^ in combined meta-analysis of all samples, *P*<10^−7^ in Kaiser Permanente non-Hispanic white non-cases, and *P*<0.10 in replication studies, with same direction of effect. A total of 38 SNPs were tested for replication. Single nucleotide polymorphisms (SNPs) associated with prostate specific antigen (PSA) levels in Kaiser Permanente non-Hispanic white non-cases and replication studies.

*, ** Associated with PCa in our study when adjusting for PSA: **P<0.00125, *0.00125<*P*<0.05.

^†^Change in log-transformed PSA per PSA level-increasing allele.

^‡^More than two alleles have been reported; other alleles are not included in the model.

**Table 3:** Conditional genome-wide association study results.

**Chrom**	**Position**	**Genes**	**SNP**	**Allele**	**Kaiser Permanente NHW non-cases (n=28,503)**			**Replication meta-analysis (*****n*****=11,825)**		**Combined meta-analysis (*****n*****=40,328)**		
					**Allele Frequency**	***β***† **(× 10**^**2**^**)**	***P*****-value**	***β***† **(× 10**^**2**^**)**	***P*****-value**	***β***† **(× 10**^**2**^**)**	***P*****-value**	**Previous PCa/PSA GWAS Hits**
3q23	141,133,450	ZBTB38^R1^	rs1991431	A/G	0.43	3.8	5.5 × 10^−9^	3.6	0.0011	3.7	2.5 × 10^−11^	S/N
8p21.2	23,466,984	SLC25A37^R1^	rs4614003	A/G	0.3	4.9	3.0 × 10^−12^	5.3	6.10 × 10^−5^	5	1.0 × 10^−15^	S/N
8q24.21	128,342,866	8q24/MYC^R1^	rs17464492	A/G	0.71	4.1	6.9 × 10^−9^	3.7	0.0055	4.0.	1.5 × 10^−10^	S*/N
10p12.1	28,094,419	ARMC4^R1^	rs2492906	G/C	0.79	4	3.2 × 10^−7^	3.0	0.0092	3.7	1.3 × 10^−8^	N/N
10q26.12	122,674,849	WDR11-FGFR2^R1^	rs200367988	A/G	0.33	5.9	1.2 × 10^−17^	3.0	0.0064	5.1	4.7 × 10^−18^	L*/L
10q26.13	123,185,303	WDR11-FGFR2^R3^	rs10749415	A/G	0.95	11.6	7.9 × 10^−16^	12.0	2.10 × 10^−10^	11.7	9.1 × 10^−25^	L/L
19q13.33	51,349,090	KLK3^R4^	rs266849	A/G	0.79	5.8	4.4 × 10^−13^	5.4	1.60 × 10^−5^	5.7	4.0 × 10^−17^	S/S
19q13.33	51,352,937	KLK3^R3^	rs266868	G/A	0.71	5.1	4.0 × 10^−13^	3.3	0.0034	4.6	1.4 × 10^−14^	L/S
19q13.33	51,354,397	KLK3^R1^	rs11665748	A/G	0.65	7.8	1.4 × 10^−30^	11.9	1.50 × 10^−27^	9	3.1 × 10^−54^	S/S
19q13.33	51,361,382	KLK3^R2^	rs61752561	G/A	0.97	18.6	1.4 × 10^−24^	10.2	0.011	17.2	2.4 × 10^−25^	L/L
19q13.33	51,373,279	KLK2^R6^	rs2739472	C/T	0.56	3.3	1.9 × 10^−7^	3.6	9.3 × 10^−4^	3.4	7.8 × 10^−10^	L*/L
19q13.33	51,380,110	KLK2^R5^	rs6070	T/A	0.65	4	4.9 × 10^−9^	6.8	1.20 × 10^−9^	4.8	4.2 × 10^−16^	L/S
Xp22.2	16,830,673	TXLNG^R1^	rs5969745	T/C	0.60	3.1	4.6 × 10^−11^	2.6	0.0012	3	2.7 × 10^−13^	N*/N

L, previously identified locus; N, novel locus; NHW, non-Hispanic White; PCa, prostate cancer; PSA, prostate-specific antigen; S, previously identified SNP; SNP, single-nucleotide polymorphism.

The replication samples for these conditional analyses are restricted to the other KP samples (that is, all KP samples except the non-Hispanic white non-cases). Listed SNPs have association *P*<5 × 10^−8^ in combined meta-analysis of all KP samples, *P*<5 × 10^−7^ in KP non-Hispanic white non-cases, and *P*<0.10 in Kaiser replication studies and with same direction of effect. A total of 23 conditional hits were tested for replication. Single nucleotide polymorphisms (SNPs) associated with prostate specific antigen (PSA) levels from conditional analyses that adjust for other significantly associated SNPs (Table 2). Full details of conditional models given in Supplementary Tables 1–3.

*, ** Associated with PCa in our study adjusting for PSA: *P<0.00125, **0.00125<*P*<0.05.

^R1^Conditional round 1, conditioning on: rs6662386, rs4951018, rs9306895, rs13395387, rs2556375, rs35350834, rs56935123, rs10023685, rs37004, rs74786629, rs6920449, rs10486567, rs13272392, rs12676621, rs10505477, rs6478343, rs59482735, rs116940348, rs10993994, rs10886902, rs4378355, rs12285347, rs11067228, rs202346, rs8023057, rs9921192, rs62046493, rs7213911, rs11263761, rs151059257, rs11084596, rs17632542, rs55891214, rs16980679, rs6627995, rs10855058, rs13441059.

^R2^Conditional round 2: R1, rs111862174, rs1991431, rs906496, rs4614003, rs17464492, rs2492906, rs200367988, rs74922337, rs9596300, rs11665748, rs5969745.

^R3^Conditional round 3: R2, rs58235267, rs4871796, rs4752569, rs66624999, rs61752561.

^R4^Conditional round 4: R3, rs10749415, rs12429206, rs266868.

^R5^Conditional round 5: R4, rs147520802, rs266849.

^R6^Conditional round 6: R5, rs6070.

^†^Change in log-transformed PSA per PSA-increasing allele.

**Table 4:** SNP risk score effects on PSA levels and variance explained (*r* ^2^).

**KP Group**	**Ages**	**s.d. of PSA levels**	**Effect (95% CI)**	***P*****-value**	***r***^**2**^
Non-Hispanic white	Overall	0.814	0.254 (0.245, 0.263)	10^−737^	0.106
Non-Hispanic white	40–50	0.728	0.224 (0.205, 0.244)	2.5 × 10^−109^	0.100
Non-Hispanic white	50–60	0.804	0.232 (0.219, 0.245)	1.4 × 10^−256^	0.087
Non-Hispanic white	60–70	0.922	0.270 (0.251, 0.282)	5.4 × 10^−176^	0.093
Non-Hispanic white	70–80	0.999	0.244 (0.206, 0.282)	1.3 × 10^−34^	0.064
Non-Hispanic white	80–90	1.196	0.254 (0.144, 0.363)	8.5 × 10^−6^	0.066
Latino	Overall	0.805	0.263 (0.233, 0.293)	7.8 × 10^−62^	0.096
East Asian	Overall	0.796	0.317 (0.277, 0.357)	5.5 × 10^−52^	0.087
African American	Overall	0.784	0.192 (0.146, 0.239)	7.5 × 10^−16^	0.040

KP, Kaiser Permanente; PSA, prostate-specific antigen; SNP, single-nucleotide polymorphism.

The effect size has been normalized to be a unit distribution across all race or ethnicity groups, so that the effect sizes can be compared.
