# Supplementary material for: Genome-wide association study of prostate-specific antigen levels identifies novel loci independent of prostate cancer
Source: Nat Commun. 2017 Jan 31;8:14248. doi: 10.1038/ncomms14248 (PMC5290311; doi:10.1038/ncomms14248)
Supplement: Supplementary Information — Supplementary Figures and Supplementary Tables [file ncomms14248-s1.pdf]

Supplementary Table 1: Unconditional results of SNPs found in KP non-Hispanic whites discovery conditional rounds. *P*-values are from linear regression. Effect sizes are the log-transformed PSA pre PSA-increasing allele multiplied by 100.

					Non-Hispanic white controls				Latino controls				East Asian controls				African American controls				Non-Hispanic white cases				Replication Meta				Combined Meta			
SNP	Chr	Pos	Round	Allele	Frq	info	Eff	P	Frq	info	Eff	P	Frq	info	Eff	P	Frq	info	Eff	P	Frq	info	Eff	P	Eff	P	I^2	Het P	Eff	P	I^2	Het P
rs111862174	1	67518976	0	T/TA	0.387	0.97	3.5	1.2e-06	0.410	0.95	-0.7	0.76	0.345	0.98	-0.5	0.82	0.430	0.88	-2.9	0.32	0.378	0.97	-0.8	0.68	-1.0	0.37	0	0.92	2.2	0.00035	66	0.019
rs58235267	2	63277843	0	G/C	0.471	0.99	3.1	4.2e-06	0.508	1.00	-0.8	0.72	0.682	0.98	4.3	0.079	0.466	0.99	-0.5	0.85	0.497	0.99	3.1	0.075	1.8	0.096	18	0.3	2.8	1.5e-06	15	0.32
rs1991431	3	141133450	0	A/G	0.431	1.00	3.4	9.4e-07	0.394	1.00	2.2	0.32	0.233	1.00	-1.7	0.52	0.666	1.00	6.3	0.032	0.446	1.00	6.5	0.00017	3.9	0.0005	63	0.043	3.5	2.2e-09	52	0.082
rs906496	4	122833314	0	C/T	0.680	0.99	3.6	7.4e-07	0.674	1.00	-3.8	0.11	0.619	1.00	-0.0	0.99	0.678	1.00	-3.5	0.24	0.681	0.99	-2.4	0.2	-2.3	0.043	0	0.68	1.9	0.0019	81	0.00034
rs4614003	8	23466984	0	A/G	0.296	1.00	4.2	2.3e-08	0.241	1.00	4.3	0.091	0.125	0.99	6.0	0.074	0.086	1.00	-2.1	0.68	0.299	1.00	4.8	0.012	4.4	0.0011	0	0.59	4.2	1e-10	0	0.74
rs17464492	8	128342866	0	A/G	0.709	1.00	4.1	5.5e-08	0.776	0.98	3.9	0.15	0.906	1.00	6.8	0.073	0.786	0.97	4.0	0.24	0.738	1.00	4.0	0.045	4.3	0.0014	0	0.92	4.1	3.2e-10	0	0.97
rs4871796	8	128473525	0	A/G	0.073	0.97	6.6	6.7e-07	0.068	0.95	-7.3	0.097	0.085	0.98	6.1	0.13	0.178	0.90	5.1	0.19	0.075	0.97	-2.9	0.39	0.3	0.89	60	0.058	4.6	3.1e-05	73	0.005
rs2492906	10	28094419	0	G/C	0.790	1.00	3.8	6.4e-06	0.652	0.99	-1.2	0.58	0.380	0.99	6.8	0.0031	0.400	0.97	4.5	0.12	0.796	1.00	2.4	0.27	2.9	0.014	55	0.085	3.5	3.5e-07	43	0.14
rs200367988	10	122674849	0	A/G	0.332	0.99	6.4	1.7e-18	0.417	0.99	4.5	0.043	0.422	0.99	2.6	0.25	0.212	0.98	0.9	0.8	0.334	0.99	3.4	0.067	3.2	0.0047	0	0.84	5.4	7.5e-19	37	0.17
rs10749415	10	123185303	0	A/G	0.948	0.98	9.7	3.1e-10	0.856	0.98	11.1	0.00035	0.978	0.99	19.4	0.012	0.797	0.96	16.1	3.6e-06	0.956	0.98	6.6	0.12	12.3	4.2e-10	24	0.27	10.7	1.4e-18	21	0.28
rs4752569	10	123331690	0	A/T	0.503	0.95	4.6	5.8e-11	0.516	0.96	-1.1	0.64	0.712	0.94	3.0	0.24	0.644	0.96	4.2	0.15	0.504	0.95	1.2	0.5	1.4	0.2	0	0.47	3.7	5e-10	51	0.088
rs147520802	11	129314183	0	A/G	0.017	0.91	13.2	1.4e-06	0.009	0.87	5.6	0.65	0.001	0.73	-43.2	0.27	0.004	0.80	9.1	0.71	0.015	0.91	2.5	0.74	2.6	0.68	0	0.68	11.5	4.5e-06	0.69	0.4
rs74922337	13	41895266	0	G/T	0.917	0.99	5.7	4.3e-06	0.940	0.99	1.9	0.68	0.972	0.99	6.5	0.33	0.974	0.99	0.8	0.93	0.929	0.99	2.6	0.44	2.8	0.25	0	0.94	5.1	3.6e-06	0	0.83
rs9596300	13	51081991	0	A/C	0.853	1.00	7.5	5e-15	0.828	0.99	0.7	0.81	0.958	1.00	2.5	0.66	0.851	0.98	9.1	0.021	0.860	1.00	3.4	0.18	3.4	0.036	0	0.39	6.5	5.4e-15	48	0.11
rs12429206	13	51446114	0	A/G	0.343	0.99	4.2	8.3e-09	0.365	0.97	2.0	0.38	0.346	0.97	1.3	0.59	0.428	0.91	0.8	0.77	0.349	0.99	3.8	0.037	2.3	0.037	0	0.77	3.6	2e-09	0	0.55
rs66624999	16	79855832	0	T/C	0.864	0.98	4.5	5.9e-06	0.869	0.95	-1.3	0.7	0.680	0.99	4.9	0.041	0.528	0.97	1.6	0.58	0.865	0.98	2.1	0.41	2.3	0.09	0	0.49	3.8	3.1e-06	5.3	0.38
rs266849	19	51349090	0	A/G	0.794	0.96	20.1	5.7e-122	0.775	1.00	21.5	2.2e-16	0.592	1.00	21.8	1.5e-22	0.877	1.00	9.7	0.023	0.815	0.96	13.4	4e-09	17.9	7.8e-44	76	0.0057	19.4	9.2e-165	72	0.0057
rs266868	19	51352937	0	A/G	0.295	1.00	1.6	0.035	0.241	1.00	1.9	0.45	0.403	1.00	-11.8	1.2e-07	0.395	1.00	-2.3	0.43	0.304	1.00	2.2	0.23	-2.3	0.045	89	6.3e-06	0.4	0.5	89	5e-07
rs11665748	19	51354397	0	A/G	0.650	0.98	17.8	4.9e-136	0.656	1.00	20.0	3.8e-18	0.546	0.98	24.7	2.4e-28	0.537	1.00	3.5	0.22	0.683	0.98	11.7	4.9e-10	15.8	4.1e-45	93	2.9e-09	17.2	3.1e-181	91	3.9e-09
rs61752561	19	51361382	0	G/A	0.965	0.91	12.2	3.2e-10	0.983	0.94	19.3	0.024	0.999	0.67	-14.4	0.73	0.988	0.93	35.8	0.0065	0.967	0.91	-2.6	0.61	6.1	0.14	72	0.014	11.1	2.5e-10	68	0.014
rs2739472	19	51373279	0	C/T	0.562	1.00	11.3	3.8e-61	0.603	1.00	13.7	1.2e-09	0.776	1.00	15.3	1.6e-08	0.665	1.00	5.5	0.059	0.578	1.00	6.2	0.00035	9.6	2e-17	78	0.0035	10.9	1.5e-77	74	0.0041
rs6070	19	51380110	0	T/A	0.654	0.94	2.8	0.00013	0.672	1.00	11.0	1.9e-06	0.748	0.93	11.6	1.2e-05	0.571	0.96	3.3	0.25	0.653	0.94	4.6	0.015	7.4	2.7e-10	67	0.026	4.1	3.9e-11	80	0.00048
rs5969745	23	16830673	0	T/C	0.597	0.96	3.2	1.3e-10	0.385	0.96	3.1	0.052	0.166	0.86	2.0	0.36	0.347	0.96	4.5	0.033	0.599	0.96	1.5	0.25	2.5	0.0031	0	0.62	3.0	2.7e-12	0	0.68

Supplementary Table 2: Conditional fits at each round at SNPs discovered in KP non-Hispanic whites. *P*-values are from linear regression. Effect sizes are the log-transformed PSA pre PSA-increasing allele multiplied by 100.

					Non-Hispanic white controls				Latino controls				East Asian controls				African American controls				Non-Hispanic white cases				Replication Meta				Combined Meta			
SNP	Chr	Pos	Ro-und	Allele	Frq	info	Eff	P	Frq	info	Eff	P	Frq	info	Eff	P	Frq	info	Eff	P	Frq	info	Eff	P	Eff	P	I^2	Het P	Eff	P	I^2	Het P
rs111862174	1	67518976	1	T/TA	0.387	0.97	3.4	4e-07	0.410	0.95	0.4	0.84	0.345	0.98	-1.2	0.59	0.430	0.88	-2.8	0.32	0.378	0.97	-0.5	0.79	-0.8	0.48	0	0.83	2.3	8.6e-05	65	0.022
rs58235267	2	63277843	2	G/C	0.471	0.99	3.2	4.9e-07	0.508	1.00	0.1	0.95	0.682	0.98	3.6	0.11	0.466	0.99	-1.9	0.48	0.497	0.99	2.3	0.18	1.4	0.19	4.1	0.37	2.7	6.4e-07	25	0.25
rs1991431	3	141133450	1	A/G	0.431	1.00	3.8	5.5e-09	0.394	1.00	1.7	0.43	0.233	1.00	-1.2	0.65	0.666	1.00	5.8	0.043	0.446	1.00	6.3	0.00032	3.6	0.0011	59	0.063	3.7	2.5e-11	45	0.12
rs906496	4	122833314	1	C/T	0.680	0.99	3.7	6.9e-08	0.674	1.00	-2.4	0.29	0.619	1.00	0.8	0.72	0.678	1.00	-3.1	0.28	0.681	0.99	-2.9	0.12	-1.9	0.091	0	0.58	2.2	0.0002	80	0.00041
rs4614003	8	23466984	1	A/G	0.296	1.00	4.9	3e-12	0.241	1.00	5.9	0.016	0.125	0.99	6.9	0.035	0.086	1.00	-1.1	0.82	0.299	1.00	5.4	0.0055	5.3	6.1e-05	0	0.58	5.0	1e-15	0	0.73
rs17464492	8	128342866	1	A/G	0.709	1.00	4.1	6.9e-09	0.776	0.98	2.9	0.26	0.906	1.00	5.6	0.13	0.786	0.97	4.1	0.22	0.738	1.00	3.5	0.084	3.7	0.0055	0	0.94	4.0	1.5e-10	0	0.98
rs4871796	8	128473525	2	A/G	0.073	0.97	6.9	3.1e-08	0.068	0.95	-6.3	0.13	0.085	0.98	4.9	0.2	0.178	0.90	4.8	0.21	0.075	0.97	-3.2	0.34	0.0	0.98	54	0.09	4.8	4.3e-06	75	0.0032
rs2492906	10	28094419	1	G/C	0.790	1.00	4.0	3.2e-07	0.652	0.99	-0.2	0.92	0.380	0.99	6.7	0.0026	0.400	0.97	4.9	0.087	0.796	1.00	1.7	0.44	3.0	0.0092	48	0.12	3.7	1.3e-08	37	0.17
rs200367988	10	122674849	1	A/G	0.332	0.99	5.9	1.2e-17	0.417	0.99	4.9	0.019	0.422	0.99	2.4	0.27	0.212	0.98	-0.5	0.88	0.334	0.99	3.0	0.1	3.0	0.0064	0	0.57	5.1	4.7e-18	41	0.15
rs10749415	10	123185303	3	A/G	0.948	0.98	11.6	7.9e-16	0.856	0.98	12.3	2.2e-05	0.978	0.99	15.7	0.032	0.797	0.96	14.8	1e-05	0.956	0.98	5.3	0.22	12.0	2.1e-10	11	0.34	11.7	9.1e-25	0	0.49
rs4752569	10	123331690	2	A/T	0.503	0.95	4.7	7.3e-13	0.516	0.96	0.1	0.95	0.712	0.94	1.6	0.52	0.644	0.96	3.8	0.18	0.504	0.95	0.7	0.71	1.2	0.28	0	0.74	3.8	1.4e-11	55	0.066
rs147520802	11	129314183	4	A/G	0.017	0.91	12.9	3.9e-07	0.009	0.87	4.8	0.68	0.001	0.73	-56.6	0.12	0.004	0.80	4.4	0.85	0.015	0.91	-0.7	0.93	-0.4	0.95	0	0.45	10.9	3.3e-06	41	0.15
rs74922337	13	41895266	1	G/T	0.917	0.99	6.1	2e-07	0.940	0.99	1.6	0.71	0.972	0.99	5.6	0.39	0.974	0.99	1.1	0.9	0.929	0.99	3.1	0.36	2.9	0.23	0	0.96	5.5	1.8e-07	0	0.78
rs9596300	13	51081991	1	A/C	0.853	1.00	5.6	6.5e-10	0.828	0.99	-0.2	0.93	0.958	1.00	0.2	0.97	0.851	0.98	5.1	0.18	0.860	1.00	1.0	0.71	1.2	0.45	0	0.72	4.5	9.8e-09	43	0.13
rs12429206	13	51446114	3	A/G	0.343	0.99	3.4	3.4e-07	0.365	0.97	2.9	0.18	0.346	0.97	1.4	0.52	0.428	0.91	1.1	0.68	0.349	0.99	2.4	0.18	2.1	0.052	0	0.95	3.1	7e-08	0	0.83
rs66624999	16	79855832	2	T/C	0.864	0.98	4.7	4.9e-07	0.869	0.95	0.8	0.79	0.680	0.99	3.8	0.088	0.528	0.97	2.2	0.42	0.865	0.98	2.2	0.38	2.5	0.054	0	0.88	4.0	1.9e-07	0	0.63
rs266849	19	51349090	4	A/G	0.794	0.96	5.8	4.4e-13	0.775	1.00	6.4	0.0088	0.592	1.00	5.0	0.019	0.877	1.00	5.3	0.19	0.815	0.96	5.0	0.028	5.4	1.6e-05	0	0.97	5.7	4e-17	0	0.99
rs266868	19	51352937	3	G/A	0.705	1.00	5.1	4e-13	0.759	1.00	5.2	0.029	0.597	1.00	2.9	0.17	0.605	1.00	2.3	0.42	0.696	1.00	2.8	0.14	3.3	0.0034	0	0.83	4.6	1.4e-14	0	0.6
rs11665748	19	51354397	1	A/G	0.650	0.98	7.8	1.4e-30	0.656	1.00	13.0	2.9e-09	0.546	0.98	24.5	1.1e-29	0.537	1.00	1.3	0.63	0.683	0.98	6.1	0.0014	11.9	1.5e-27	95	1e-12	9.0	3.1e-54	94	4e-14
rs61752561	19	51361382	2	G/A	0.965	0.91	18.6	1.4e-24	0.983	0.94	25.6	0.0015	0.999	0.67	-7.6	0.85	0.988	0.93	32.7	0.0099	0.967	0.91	0.8	0.88	10.2	0.011	71	0.015	17.2	2.4e-25	71	0.0073
rs2739472	19	51373279	6	C/T	0.562	1.00	3.3	1.9e-07	0.603	1.00	4.0	0.053	0.776	1.00	6.2	0.015	0.665	1.00	4.8	0.086	0.578	1.00	1.6	0.36	3.6	0.00093	0	0.47	3.4	7.8e-10	0	0.63
rs6070	19	51380110	5	T/A	0.654	0.94	4.0	4.9e-09	0.672	1.00	10.2	1.9e-06	0.748	0.93	8.1	0.0011	0.571	0.96	4.3	0.11	0.653	0.94	4.6	0.016	6.8	1.2e-09	41	0.17	4.8	4.2e-16	58	0.048
rs5969745	23	16830673	1	T/C	0.597	0.96	3.1	4.6e-11	0.385	0.96	3.5	0.02	0.166	0.86	1.9	0.38	0.347	0.96	5.0	0.015	0.599	0.96	1.4	0.29	2.6	0.0012	0	0.43	3.0	2.7e-13	0	0.55

Round 1, conditioning additionally on: rs6662386, rs4951018, rs9306895, rs13395387, rs2556375, rs35350834, rs56935123, rs10023685, rs37004, rs74786629, rs6920449, rs10486567, rs13272392, rs12676621, rs10505477, rs6478343, rs59482735, rs116940348, rs10993994, rs10886902, rs4378355, rs12285347, 12, rs202346, rs8023057, rs9921192, rs62046493, rs7213911, rs11263761, rs151059257, rs11084596, rs17632542, rs55891214, rs16980679, rs6627995, rs10855058, rs13441059
Round 2: R1, rs111862174, rs1991431, rs906496, rs4614003, rs17464492, rs2492906, rs200367988, rs74922337, rs9596300, rs11665748, rs5969745
Round 3: R2, rs58235267, rs4871796, rs4752569, rs66624999, rs61752561
Round 4: R3, rs10749415, rs12429206, rs266868
Round 5: R4, rs147520802, rs266849
Round 6: R5, rs6070

Supplementary Table 3: Joint fit of all KP non-Hispanic whites discovered SNPs at loci with more than one significant SNP. P-values are from linear regression. Effect sizes are the log-transformed PSA pre PSA-increasing allele multiplied by 100.

		Non-Hispanic white controls		Latino controls		East Asian controls		African American controls		Non-Hispanic white cases		Replication Meta				Combined Meta			
Allele	SNP	Eff	P	Eff	P	Eff	P	Eff	P	Eff	P	Eff	P	I^2	Het p	Eff	P	I^2	Het p
A/G	rs4614003	5.3	1.9e-12	5.4	0.041	7.6	0.03	-0.5	0.92	5.5	0.0043	5.4	8.7e-05	0	0.65	5.4	7.6e-16	0	0.8
T/A	rs13272392	7.9	1.3e-29	7.3	0.0016	9.4	0.00017	6.9	0.021	5.0	0.0047	6.7	3e-09	0	0.54	7.6	3.9e-37	0	0.57
A/G	rs17464492	3.5	2.9e-06	3.8	0.17	7.6	0.055	3.8	0.27	3.9	0.054	4.3	0.0018	0	0.85	3.7	2.2e-08	0	0.91
A/G	rs10505477	5.1	2.1e-13	6.6	0.003	4.2	0.066	2.2	0.54	4.2	0.017	4.7	3.8e-05	0	0.72	5.0	4e-17	0	0.84
A/G	rs4871796	5.9	1.2e-05	-8.3	0.064	6.3	0.12	4.8	0.23	-4.0	0.23	-0.4	0.86	65	0.034	3.9	0.00045	74	0.0037
A/G	rs200367988	6.3	2.8e-18	5.1	0.026	2.6	0.25	0.5	0.89	3.4	0.067	3.3	0.004	0	0.72	5.5	5.2e-19	36	0.18
C/T	rs10886902	11.0	1.4e-42	7.9	0.0023	11.0	1.7e-05	10.8	0.06	4.1	0.052	7.3	4.1e-08	39	0.18	10.0	6e-48	62	0.034
A/G	rs10749415	11.4	2.5e-13	12.0	0.00023	19.1	0.016	15.7	9.3e-06	7.1	0.1	13.0	5.2e-10	3.4	0.38	12.0	9e-22	0	0.5
A/T	rs4752569	5.4	1.1e-14	-0.3	0.89	3.1	0.22	4.9	0.097	1.6	0.38	1.9	0.096	0	0.52	4.5	9.1e-14	56	0.057
A/C	rs9596300	6.1	9.2e-10	-1.1	0.72	0.7	0.9	7.3	0.082	1.4	0.58	1.6	0.36	0	0.45	4.9	8.8e-09	49	0.096
A/C	rs202346	4.9	2.7e-09	6.0	0.027	8.5	0.0083	7.1	0.026	5.7	0.0052	6.5	9.7e-07	0	0.89	5.3	2.3e-14	0	0.78
A/G	rs12429206	3.7	3.8e-07	1.9	0.4	0.9	0.71	0.9	0.75	3.2	0.079	2.0	0.073	0	0.85	3.2	1.6e-07	0	0.68
A/G	rs266849	8.1	2e-16	9.8	0.002	8.1	0.0088	7.3	0.11	6.7	0.0097	7.9	5.6e-07	0	0.9	8.1	6.3e-22	0	0.96
G/A	rs266868	4.4	1.8e-06	2.2	0.44	4.9	0.066	3.4	0.27	2.3	0.33	3.2	0.019	0	0.88	4.0	1.4e-07	0	0.88
A/G	rs11665748	6.7	2.5e-10	8.0	0.016	14.7	1.6e-05	0.4	0.89	5.8	0.039	6.7	1.6e-05	70	0.018	6.7	1.9e-14	60	0.039
G/A	rs61752561	20.9	4.6e-24	33.8	0.00012	8.5	0.84	43.1	0.0017	3.1	0.57	15.0	0.0007	78	0.0037	20.0	3.1e-26	73	0.0046
T/C	rs17632542	37.4	1.5e-151	38.9	7.5e-13	13.7	0.38	35.3	0.00069	20.4	9e-07	27.0	5.6e-19	65	0.034	36.0	7.1e-167	77	0.0016
C/T	rs2739472	6.2	2.3e-12	6.2	0.028	9.2	0.0027	6.0	0.052	2.8	0.22	5.6	5.8e-05	0	0.41	6.0	6.6e-16	0	0.55
T/A	rs6070	5.5	3.1e-13	12.1	1.7e-07	9.4	0.00041	5.7	0.054	5.2	0.0076	7.9	2.5e-11	51	0.11	6.2	2.4e-22	57	0.056

Supplementary Table 4: Linkage disequilibrium at the *FGFR* gene.

FGFR r^2/D'	rs200367988	rs10886902	rs10749415	BP
rs200367988	1	0.00058/0.031	0.0016/0.12	122674849 (+0)
rs10886902	0.00058/0.031	1	0.00058/0.19	123049264 (+374,415)
rs10749415	0.0016/0.12	0.00058/0.19	1	123185303 (+510,454)

Supplementary Table 5: Association with *FGFR* haplotypes (each allele corresponds to the order of the SNPs above), for PSA, for PCa without adjusting for PSA, and for PCa adjusting for PSA. *P*-values are from linear (PSA) or logistic (PCa) regression. PCa, prostate cancer.

		PSA		PCa no PSA adj.		PCa adj. PSA	
Haplotype	Freq.	Effect ($\times 10^{-2}$)	P	OR	P	OR	P
ATG	0.016	-13.3	6.4e-05	0.733	0.0073	0.874	0.28
GTG	0.025	-16.8	3.0e-11	0.781	0.0047	1.01	0.93
ACA	0.078	20.6	4.1e-43	0.994	0.90	0.764	5.2e-07
GCA	0.233	3.0	0.00048	1.04	0.12	1	0.89
ATA	0.148	9.1	4.4e-18	0.949	0.13	0.845	7.9e-06
GTA	0.491	-8.9	1.4e-35	1.04	0.11	1.16	2.6e-09

Supplementary Table 6: Linkage disequilibrium at the *KLK* region.

KLK r2/D'	rs266849	rs266868	rs11665748	rs61752561	rs17632542	rs2739472	rs6070	BP
rs266849	1/1	0.060/0.76	0.18/0.61	0.0088/0.98	0.14/0.68	0.0038/0.11	0.0056/0.11	51349090 (+0)
rs266868	0.060/0.76	1/1	0.17/0.87	0.074/0.93	0.031/0.99	0.011/0.15	0.00098/0.066	51352937 (+3,847)
rs11665748	0.18/0.61	0.17/0.87	1/1	0.019/1	0.14/0.98	0.23/0.58	0.000020/0.0085	51354397 (+53,07)
rs61752561	0.0088/0.98	0.074/0.93	0.019/1	1/1	0.0030/1	0.027/0.98	0.012/0.79	51361382 (+12,292)
rs17632542	0.14/0.68	0.031/0.99	0.14/0.98	0.0030/1	1/1	0.031/0.56	0.0013/0.18	51361757 (+12,667)
rs2739472	0.0038/0.11	0.011/0.15	0.23/0.58	0.027/0.98	0.031/0.56	1/1	0.039/0.27	51373279 (+24,189)
rs6070	0.0056/0.11	0.00098/0.066	0.000020/0.0085	0.012/0.79	0.0013/0.18	0.039/0.27	1/1	51380110 (+31,020)

Supplementary Table 7: Association with *KLK* region haplotypes (each allele corresponds to the order of the SNPs above), for PSA, for PCa without adjusting for PSA, and for PCa adjusting for PSA. *P*-values are from linear (PSA) or logistic (PCa) regression. PCa, prostate cancer.

		PSA		PCa no PSA adj.		PCa adj. PSA	
Haplotype	Freq.	Effect ($\times 10^{-2}$)	P	OR	P	OR	P
CGGGGTT	0.042	-50.6	8.5e-186	0.709	6.5e-08	1.31	0.00012
TAAAGTT	0.106	4.8	3.3e-05	1.04	0.25	0.992	0.85
TAGAGCT	0.204	21.1	2.0e-111	1.09	0.0030	0.881	0.00011
TGGGGCT	0.029	-5.0	0.033	0.899	0.17	0.956	0.6
TAGAGCA	0.107	10.9	8.0e-17	1.09	0.040	0.961	0.38
TGGAGCT	0.023	4.9	0.090	1.09	0.36	1.04	0.70
TGGAGCA	0.019	-0.4	0.89	1.18	0.048	1.21	0.039
CGGGGTA	0.010	-85.6	1.2e-85	0.655	0.0079	1.86	0.00017
TAAAGCA	0.037	4.4	0.032	1.13	0.047	1.07	0.31
TAGGGTA	0.060	-4.7	0.0067	0.962	0.49	1.06	0.35
TAAAGTA	0.045	-3.2	0.12	1	0.94	1.08	0.28
TAAAGCT	0.059	10.0	4.1e-09	1.12	0.032	1.01	0.81
TAAAACT	0.024	-12.4	1.1e-07	0.91	3 0.24	0.99	0.92
TAGGGTT	0.098	1.4	0.25	0.986	0.71	0.974	0.54
TGGGGTT	0.029	-15.3	8.4e-10	0.886	0.14	1.11	0.25
TAGAGTT	0.011	5.4	0.14	1.04	0.71	1.03	0.80
TGGGGTA	0.020	-20.1	3.4e-11	0.753	0.0074	0.946	0.63
CAGGGCT	0.011	-49.4	1.5e-45	0.657	0.0012	1.14	0.35

Supplementary Table 8: Prostate cancer results for SNPs associated with PSA levels or PCa in KP non-Hispanic whites. *P*-values are from logistic regression.

					Control	Case	No PSA Adjustment		Adjusted for PSA	
SNP	Type	Chr	Pos	Allele	Freq	Case	OR	P	OR	P
rs636291	PCa	1	10556097	G/A	0.315	0.310	0.974	0.26	0.979	0.42
rs6662386	PSA	1	88190037	C/T	0.439	0.442	1.009	0.69	0.947	0.024
rs17599629	PCa	1	150658287	A/G	0.789	0.771	0.896	2.8e-05	0.894	0.00012
rs1218582	PCa	1	154834183	G/A	0.461	0.477	1.072	0.0018	1.037	0.14
rs4245739	PCa	1	204518842	C/A	0.275	0.259	0.923	0.0013	0.944	0.034
rs4951018	PSA	1	205636334	A/C	0.777	0.777	1.010	0.71	1.059	0.06
rs1775148	PCa	1	205757824	C/T	0.358	0.361	1.008	0.73	0.971	0.24
rs11902236	PCa	2	10117868	C/T	0.712	0.709	0.982	0.46	0.983	0.52
rs9287719	PCa	2	10710730	C/T	0.492	0.500	1.045	0.046	1.056	0.025
rs13385191	PCa	2	20888265	A/G	0.759	0.751	0.953	0.054	0.993	0.82
rs1465618	PCa	2	43553949	T/C	0.208	0.214	1.024	0.38	1.002	0.94
rs2556375	PSA	2	60759747	G/T	0.171	0.169	0.989	0.7	0.888	0.00025
rs721048	PCa	2	63131731	G/A	0.816	0.802	0.917	0.0016	0.932	0.02
rs10187424	PCa	2	85794297	T/C	0.579	0.598	1.087	0.00019	1.113	1.3e-05
rs12621278	PCa	2	173311553	A/G	0.941	0.951	1.212	0.00011	1.195	0.0011
rs2292884	PCa	2	238443226	A/G	0.763	0.753	0.942	0.035	0.964	0.23
rs3771570	PCa	2	242382864	C/T	0.854	0.845	0.926	0.011	0.917	0.009
rs2660753	PCa	3	87110674	T/C	0.116	0.126	1.118	0.00076	1.125	0.0013
rs2055109	PCa	3	87467332	C/T	0.243	0.253	1.058	0.024	1.055	0.053
rs7611694	PCa	3	113275624	A/C	0.598	0.613	1.071	0.0023	1.027	0.27
rs10934853	PCa	3	128038373	C/A	0.718	0.703	0.931	0.003	0.953	0.069
rs6763931	PCa	3	141102833	G/A	0.566	0.551	0.945	0.0093	1.002	0.95
rs1991431	PSA	3	141133450	G/A	0.569	0.554	0.939	0.0044	0.993	0.77
rs10936632	PCa	3	170130102	C/A	0.502	0.489	0.949	0.018	0.959	0.089
rs10009409	PCa	4	73855253	C/T	0.696	0.690	0.976	0.3	0.969	0.22
rs1894292	PCa	4	74349158	G/A	0.525	0.537	1.047	0.036	1.040	0.1
rs12500426	PCa	4	95514609	A/C	0.459	0.475	1.072	0.0013	1.060	0.014
rs17021918	PCa	4	95562877	C/T	0.651	0.664	1.060	0.012	1.047	0.072
rs7679673	PCa	4	106061534	C/A	0.586	0.610	1.109	3.9e-06	1.084	0.0011
rs56935123	PSA	4	146874227	A/AT	0.553	0.551	0.991	0.69	1.041	0.11
rs10023685	PSA	4	157534249	C/G	0.630	0.633	1.015	0.5	1.062	0.016
rs2242652	PCa	5	1280028	G/A	0.812	0.816	1.056	0.16	1.025	0.55
rs37004	PSA	5	1356684	C/T	0.794	0.792	0.981	0.55	0.895	0.0023
rs12653946	PCa	5	1895829	C/T	0.565	0.556	0.944	0.027	0.967	0.25
rs2121875	PCa	5	44365545	C/A	0.331	0.328	0.984	0.49	0.973	0.28
rs6869841	PCa	5	172939426	C/T	0.785	0.782	0.977	0.38	0.997	0.93
rs4713266	PCa	6	11219030	C/T	0.504	0.510	1.024	0.27	1.021	0.38
rs7767188	PCa	6	30073776	G/A	0.804	0.794	0.947	0.049	0.991	0.76
rs130067	PCa	6	31118511	T/G	0.806	0.809	1.029	0.3	1.043	0.17
rs3096702	PCa	6	32192331	A/G	0.353	0.359	1.018	0.43	1.008	0.76
rs3129859	PCa	6	32400939	G/C	0.700	0.698	1.002	0.93	0.969	0.22
rs1983891	PCa	6	41536427	C/T	0.731	0.713	0.917	0.00034	0.935	0.012
rs6920449	PSA	6	43710348	T/C	0.157	0.165	1.062	0.045	0.975	0.45
rs9443189	PCa	6	76495882	A/G	0.861	0.865	1.039	0.22	1.025	0.48
rs2273669	PCa	6	109285189	A/G	0.857	0.844	0.895	0.00024	0.902	0.0021
rs339331	PCa	6	117210052	T/C	0.703	0.724	1.115	8.6e-06	1.079	0.0048
rs1933488	PCa	6	153441079	A/G	0.578	0.586	1.033	0.14	1.037	0.14
rs9364554	PCa	6	160833664	C/T	0.726	0.703	0.894	2.7e-06	0.925	0.0033
rs12155172	PCa	7	20994491	A/G	0.215	0.224	1.055	0.04	1.012	0.68
rs10486567	PSA	7	27976563	G/A	0.761	0.786	1.163	1.1e-08	1.072	0.016
rs10486567	PCa	7	27976563	G/A	0.761	0.786	1.163	1.1e-08	1.072	0.016
rs56232506	PCa	7	47437244	G/A	0.536	0.533	0.985	0.5	0.989	0.66
rs6465657	PCa	7	97816327	C/T	0.450	0.471	1.076	0.00087	1.048	0.055
rs2928679	PCa	8	23438975	A/G	0.460	0.467	1.032	0.14	0.981	0.43
rs4614003	PSA	8	23466984	A/G	0.296	0.299	1.039	0.12	0.980	0.44
rs1512268	PCa	8	23526463	T/C	0.426	0.459	1.146	4.8e-10	1.062	0.012
rs13272392	PSA	8	23528511	T/A	0.425	0.458	1.146	5.2e-10	1.060	0.016
rs11135910	PCa	8	25892142	C/T	0.842	0.839	0.979	0.48	0.986	0.67
rs12543663	PCa	8	127924659	C/A	0.299	0.316	1.082	0.00098	1.072	0.0079
rs10086908	PCa	8	128011937	T/C	0.701	0.731	1.165	4.5e-10	1.127	8.6e-06
rs1016343	PCa	8	128093297	C/T	0.800	0.760	0.793	2e-19	0.822	5.8e-12
rs13252298	PCa	8	128095156	A/G	0.705	0.722	1.088	0.00047	1.084	0.0027
rs6983561	PCa	8	128106880	A/C	0.968	0.951	0.630	1.1e-18	0.649	1.3e-13
rs116041037	PCa	8	128131809	G/A	1.000	1.000	0.642	0.59	0.841	0.87
rs445114	PCa	8	128323181	T/C	0.626	0.654	1.127	1.7e-07	1.074	0.0046
rs16902104	PCa	8	128340908	C/G	1.000	1.000	2.454	0.41	6.314	0.15
rs17464492	PSA	8	128342866	A/G	0.709	0.738	1.133	4e-07	1.076	0.0071
rs10505477	PSA	8	128407443	A/G	0.495	0.546	1.233	1.6e-21	1.163	4.3e-10
rs6983267	PCa	8	128413305	G/T	0.505	0.557	1.239	2.6e-22	1.166	2.6e-10
rs7000448	PCa	8	128441170	C/T	0.633	0.611	0.904	6.6e-06	0.937	0.0082
rs11986220	PCa	8	128531689	A/T	0.092	0.135	1.549	5.5e-40	1.484	1.1e-26
rs17694493	PCa	9	22041998	C/G	0.862	0.852	0.925	0.012	0.933	0.041
rs817826	PCa	9	110156300	C/T	0.153	0.149	0.977	0.46	0.986	0.67
rs6478343	PSA	9	120732749	T/C	0.181	0.187	1.056	0.054	1.120	0.00025
rs59482735	PSA	9	123643426	T/TAA	0.318	0.325	1.032	0.18	0.984	0.52
rs1571801	PCa	9	124427373	G/T	0.748	0.742	0.974	0.28	0.957	0.11
rs116940348	PSA	10	22581581	G/A	0.970	0.973	1.131	0.092	1.376	5.8e-05
rs2492906	PSA	10	28094419	C/G	0.210	0.204	0.952	0.07	0.993	0.82
rs76934034	PCa	10	46082985	T/C	0.914	0.923	1.121	0.0049	1.091	0.052
rs10993994	PSA	10	51549496	T/C	0.393	0.439	1.219	2e-19	1.109	1.8e-05
rs10993994	PCa	10	51549496	T/C	0.393	0.439	1.219	2e-19	1.109	1.8e-05
rs3850699	PCa	10	104414221	A/G	0.701	0.716	1.071	0.0046	1.049	0.072
rs200367988	PSA	10	122674849	G/A	0.668	0.666	0.989	0.63	1.073	0.0061
rs2252004	PCa	10	122844709	C/A	0.907	0.908	1.022	0.56	1.012	0.78
rs10886902	PSA	10	123049264	T/C	0.762	0.769	1.042	0.11	1.206	6.2e-11
rs10749415	PSA	10	123185303	G/A	0.052	0.044	0.820	0.00018	0.948	0.36
rs4962416	PCa	10	126696872	T/C	0.718	0.714	0.980	0.43	0.994	0.82
rs7127900	PCa	11	2233574	A/G	0.198	0.211	1.116	0.00053	1.085	0.02
rs4378355	PSA	11	34783417	G/C	0.612	0.606	0.980	0.37	1.033	0.19
rs1938781	PCa	11	58915110	A/G	0.795	0.786	0.954	0.074	0.958	0.14
rs10896449	PCa	11	68994667	A/G	0.491	0.443	0.812	3.3e-21	0.838	2.1e-13
rs12285347	PSA	11	102396607	T/C	0.543	0.557	1.053	0.017	1.118	3.5e-06
rs11568818	PCa	11	102401661	T/C	0.543	0.557	1.053	0.018	1.116	4.9e-06
rs11214775	PCa	11	113807181	G/A	0.700	0.712	1.056	0.022	1.043	0.11
12:48419618	PCa	12	48419618	A/C	0.914	0.923	1.136	0.0019	1.147	0.0022
12:49676010	PCa	12	49676010	T/C	0.710	0.688	0.899	1e-05	0.893	2.3e-05
12:53273904	PCa	12	53273904	G/A	0.856	0.839	0.873	5.6e-06	0.894	0.00074
12:114685571	PCa	12	114685571	A/G	0.480	0.497	1.071	0.0017	1.066	0.0078

SNP	Type	Chr	Pos	Allele	Control Freq	Case Case	No PSA Adjustment OR	P	Adjusted for PSA OR	P
12:115094260	PSA	12	115094260	A/G	0.543	0.553	1.044	0.052	0.974	0.28
rs202346	PSA	13	51087443	C/A	0.746	0.732	0.928	0.0023	1.011	0.69
rs9600079	PCa	13	73728139	G/T	0.552	0.541	0.954	0.03	0.939	0.0089
rs8008270	PCa	14	53372330	T/C	0.194	0.183	0.932	0.012	0.928	0.016
rs7153648	PCa	14	61122526	C/G	0.091	0.096	1.075	0.057	1.083	0.059
rs7141529	PCa	14	69126744	T/C	0.493	0.484	0.961	0.066	0.959	0.084
rs8014671	PCa	14	71092256	G/A	0.575	0.582	1.026	0.25	1.039	0.12
rs8023057	PSA	14	95097556	A/G	0.824	0.833	1.071	0.019	0.983	0.58
rs9921192	PSA	16	4349111	T/C	0.502	0.501	0.996	0.84	1.048	0.063
rs12051443	PCa	16	71691329	G/A	0.667	0.664	0.989	0.64	1.001	0.95
rs684232	PCa	17	618965	T/C	0.651	0.636	0.941	0.0081	0.956	0.075
rs11649743	PCa	17	36074979	G/A	0.812	0.831	1.146	2.4e-06	1.125	0.00021
rs11263761	PSA	17	36097775	G/A	0.495	0.443	0.812	1.5e-20	0.868	8.4e-09
rs7501939	PCa	17	36101156	T/C	0.404	0.362	0.839	6.4e-15	0.888	1.8e-06
rs11650494	PCa	17	47345186	G/A	0.915	0.910	0.914	0.02	0.948	0.21
rs7210100	PCa	17	47436749	G/A	1.000	1.000	1.473	0.72	1.524	0.7
rs1859962	PCa	17	69108753	G/T	0.478	0.507	1.124	8.1e-08	1.122	1.6e-06
rs7241993	PCa	18	76773973	C/T	0.694	0.700	1.030	0.22	0.999	0.97
rs11084596	PSA	19	32104979	T/C	0.609	0.607	0.991	0.68	0.919	0.0011
rs8102476	PCa	19	38735613	C/T	0.559	0.575	1.073	0.0016	1.064	0.012
rs11672691	PCa	19	41985587	A/G	0.254	0.253	0.993	0.78	0.978	0.42
rs266849	PSA	19	51349090	G/A	0.206	0.185	0.959	0.19	1.054	0.14
rs266868	PSA	19	51352937	G/A	0.705	0.697	1.017	0.52	0.944	0.052
rs11665748	PSA	19	51354397	G/A	0.350	0.317	0.901	3.4e-05	1.013	0.63
rs61752561	PSA	19	51361382	G/A	0.965	0.967	1.139	0.043	0.968	0.65
rs17632542	PSA	19	51361757	T/C	0.924	0.943	1.348	1.1e-10	0.770	4.9e-07
rs2735839	PCa	19	51364623	A/G	0.154	0.134	0.844	9.4e-08	1.146	0.00012
rs2739472	PSA	19	51373279	T/C	0.438	0.421	0.991	0.75	1.078	0.02
rs6070	PSA	19	51380110	T/A	0.654	0.653	1.003	0.89	0.957	0.089
rs103294	PCa	19	54797848	C/T	0.806	0.798	0.957	0.11	0.953	0.11
rs12480328	PCa	20	49527922	T/C	0.929	0.930	1.015	0.72	0.979	0.64
rs2427345	PCa	20	61015611	C/T	0.609	0.612	1.027	0.4	0.991	0.79
rs6062509	PCa	20	62362563	G/T	0.310	0.300	0.950	0.033	0.937	0.013
rs1041449	PCa	21	42901421	A/G	0.578	0.573	0.985	0.5	0.987	0.61
rs2238776	PCa	22	19757892	G/A	0.802	0.803	1.006	0.85	0.996	0.9
rs9623117	PCa	22	40452119	T/C	0.774	0.767	0.953	0.067	0.961	0.17
rs5759167	PCa	22	43500212	G/T	0.499	0.526	1.123	2.8e-07	1.116	1e-05
rs2405942	PCa	23	9814135	G/A	0.217	0.204	0.962	0.045	0.964	0.083
rs5969745	PSA	23	16830673	T/C	0.598	0.598	1.005	0.75	0.964	0.036
rs16980679	PSA	23	17820345	G/A	0.951	0.953	1.027	0.46	0.909	0.018
rs6627995	PSA	23	24059175	T/C	0.330	0.335	1.014	0.41	0.965	0.056
rs5945572	PCa	23	51229683	A/G	0.348	0.373	1.058	0.00043	1.035	0.051
rs2807031	PCa	23	52896949	C/T	0.175	0.195	1.069	0.00065	1.043	0.053
rs10855058	PSA	23	55936822	A/G	0.296	0.309	1.035	0.04	1.004	0.82
rs5919432	PCa	23	67021550	C/T	0.204	0.187	0.950	0.0092	0.970	0.15
rs6625711	PCa	23	70139850	A/T	0.446	0.455	1.025	0.14	1.002	0.93
rs4844289	PCa	23	70407983	A/G	0.599	0.592	0.981	0.23	0.983	0.31

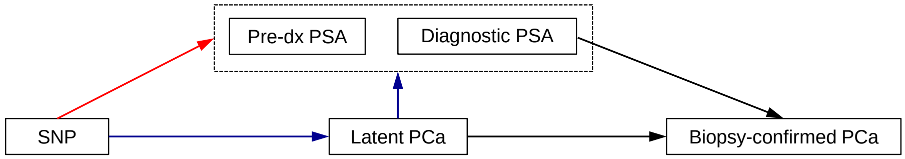

Supplementary Figure 1: Directed acyclic graph (DAG) displaying potential causal relationships between genetic polymorphisms, elevated PSA levels in blood, and prostate cancer incidence. The dotted box highlights potentially reppeated measurements. Genetic variants may cause increases in PSA levels with (blue) or without (red) also causing prostate cancer. It is assumed that elevated PSA levels ware representative of causes of clinically-confirmed, but not latent, prostate cancer. Diagnosis, dx; PCa, prostate cancer.

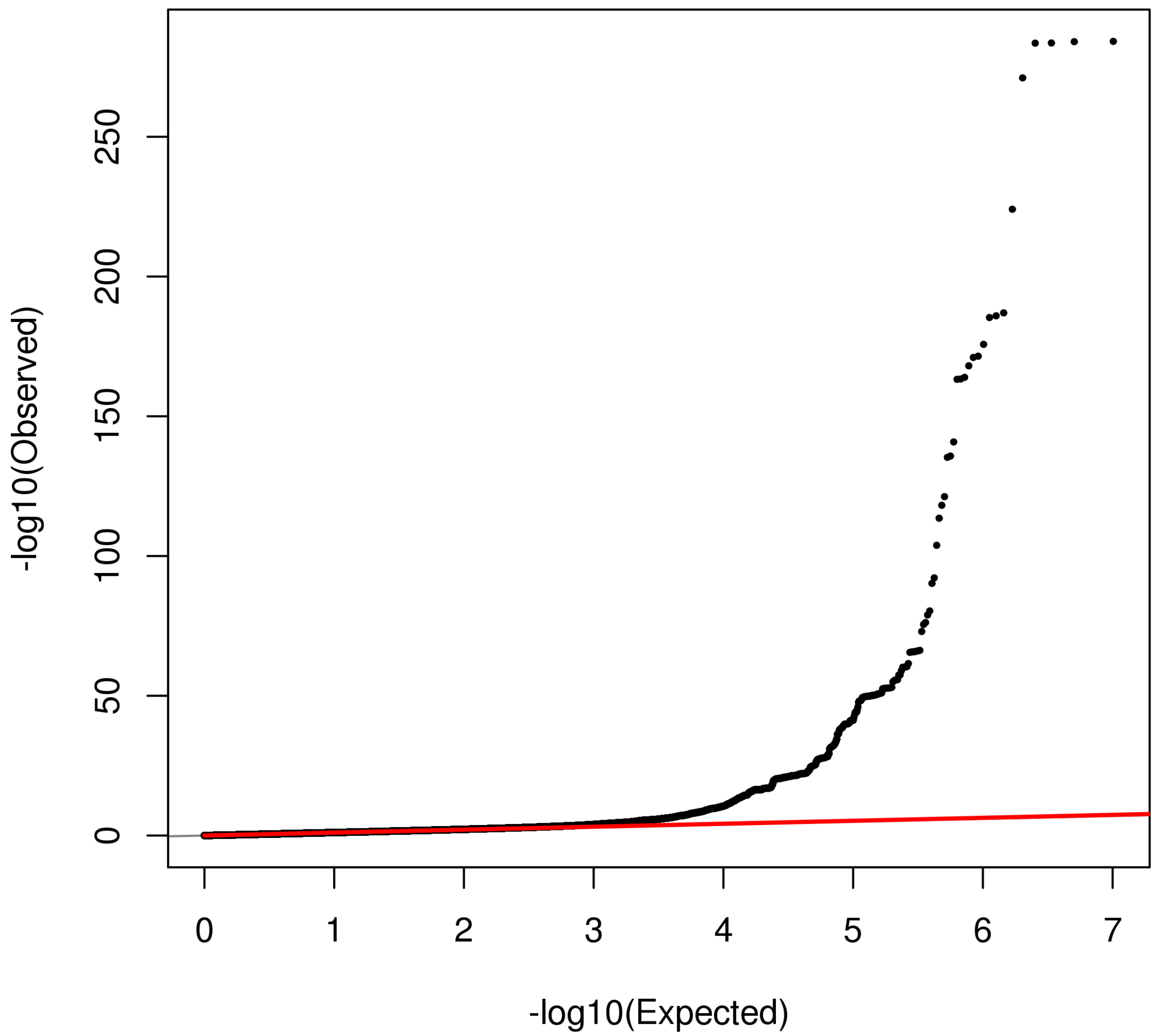

Supplementary Figure 2: Q-Q plot of GWAS results for the KP non-Hispanic whites discovery cohort. P-values are from linear regression.

rs6662386

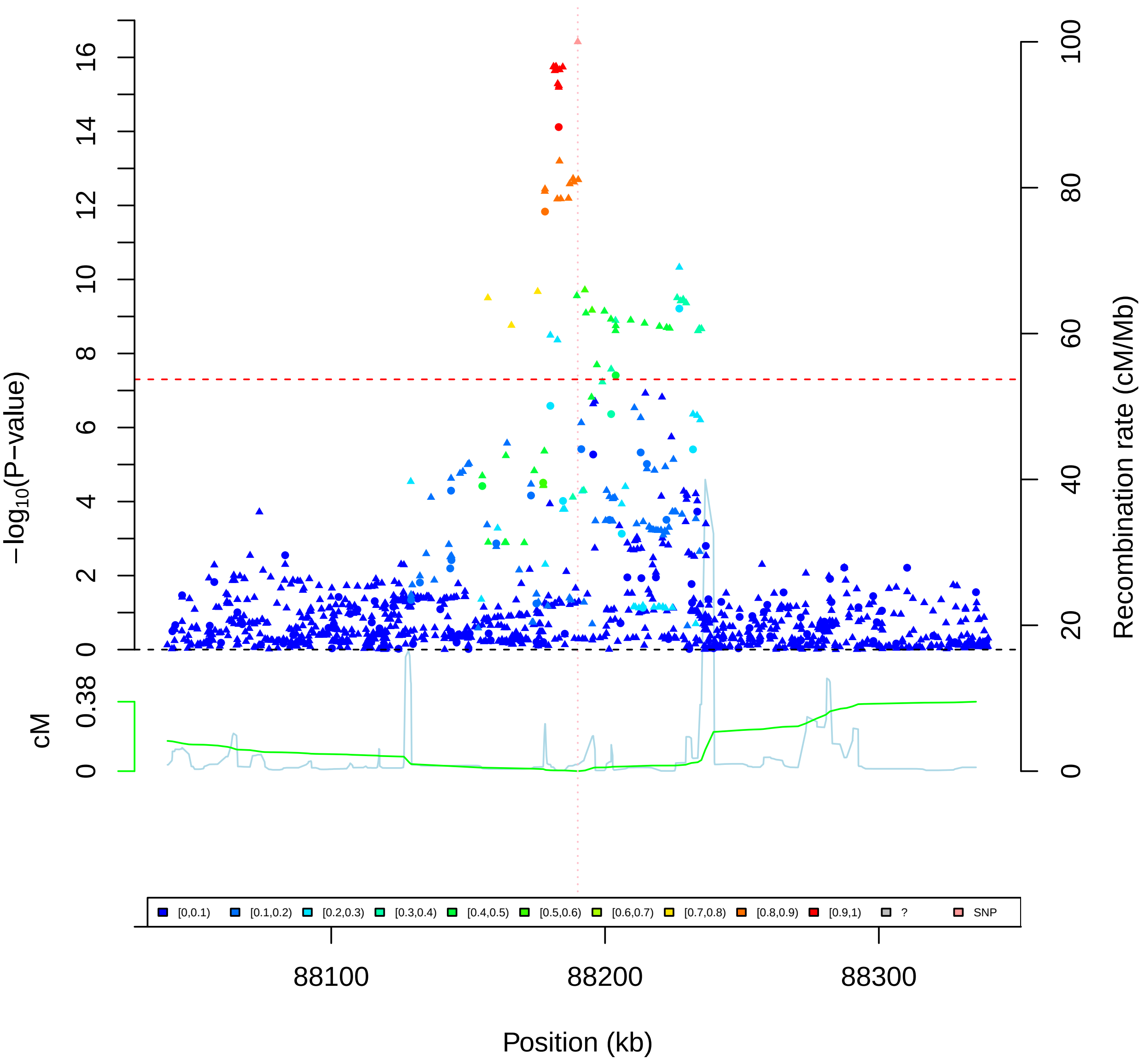

Supplementary Figure 3: Locus-specific plots of significant GWAS regions for KP non-Hispanic whites. In the plots, genotyped SNPs are given by circles, imputed SNPs are given by triangles, and the color scale indicates the correlation to the top locus as defined in the legend below each plot. *P*-values are from linear regression.

rs4951018

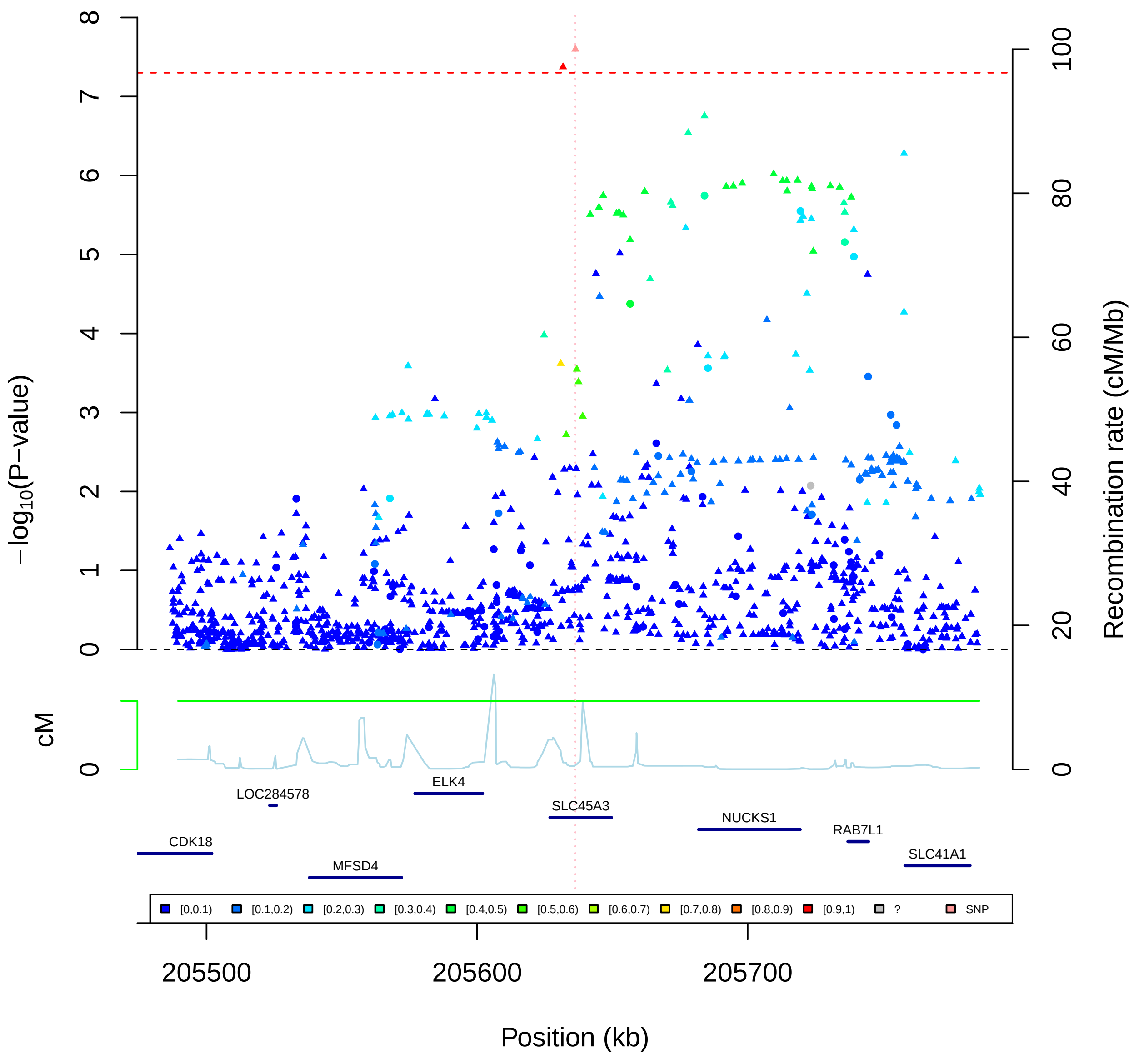

Supplementary Figure 3: Continued from previous page.

rs2556375

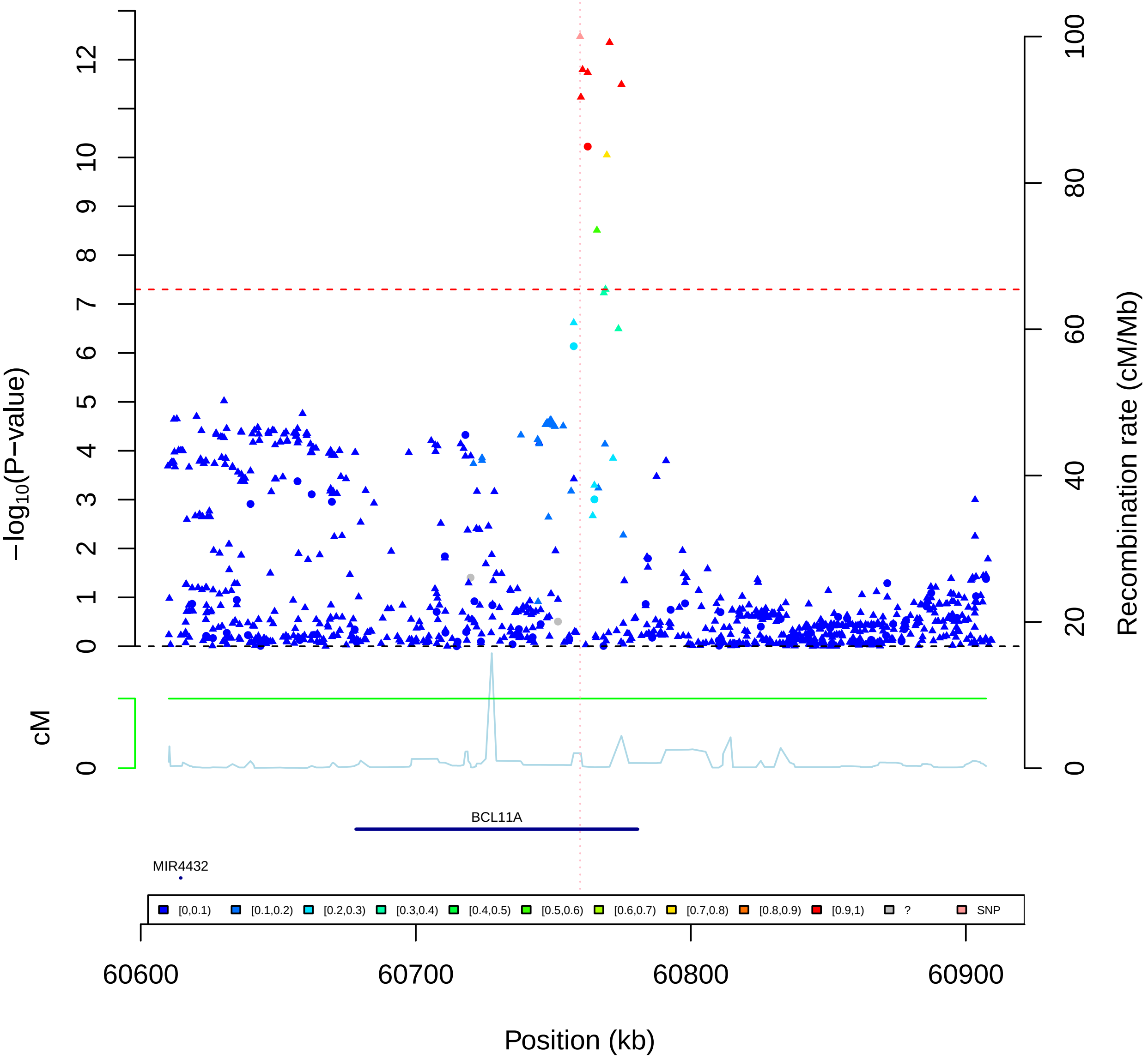

Supplementary Figure 3: Continued from previous page.

rs1991431 (Conditional round 1)

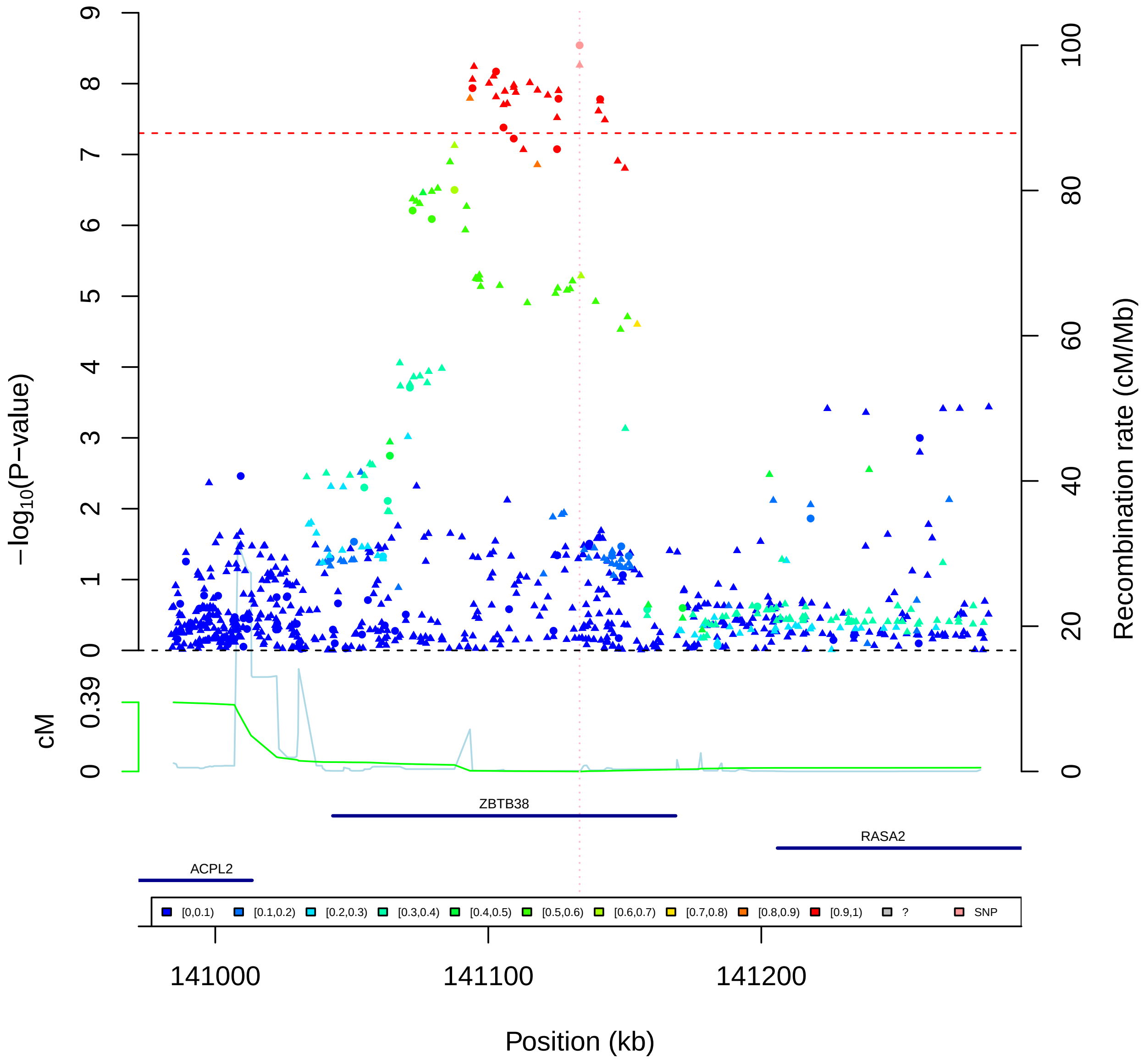

Supplementary Figure 3: Continued from previous page.

rs56935123

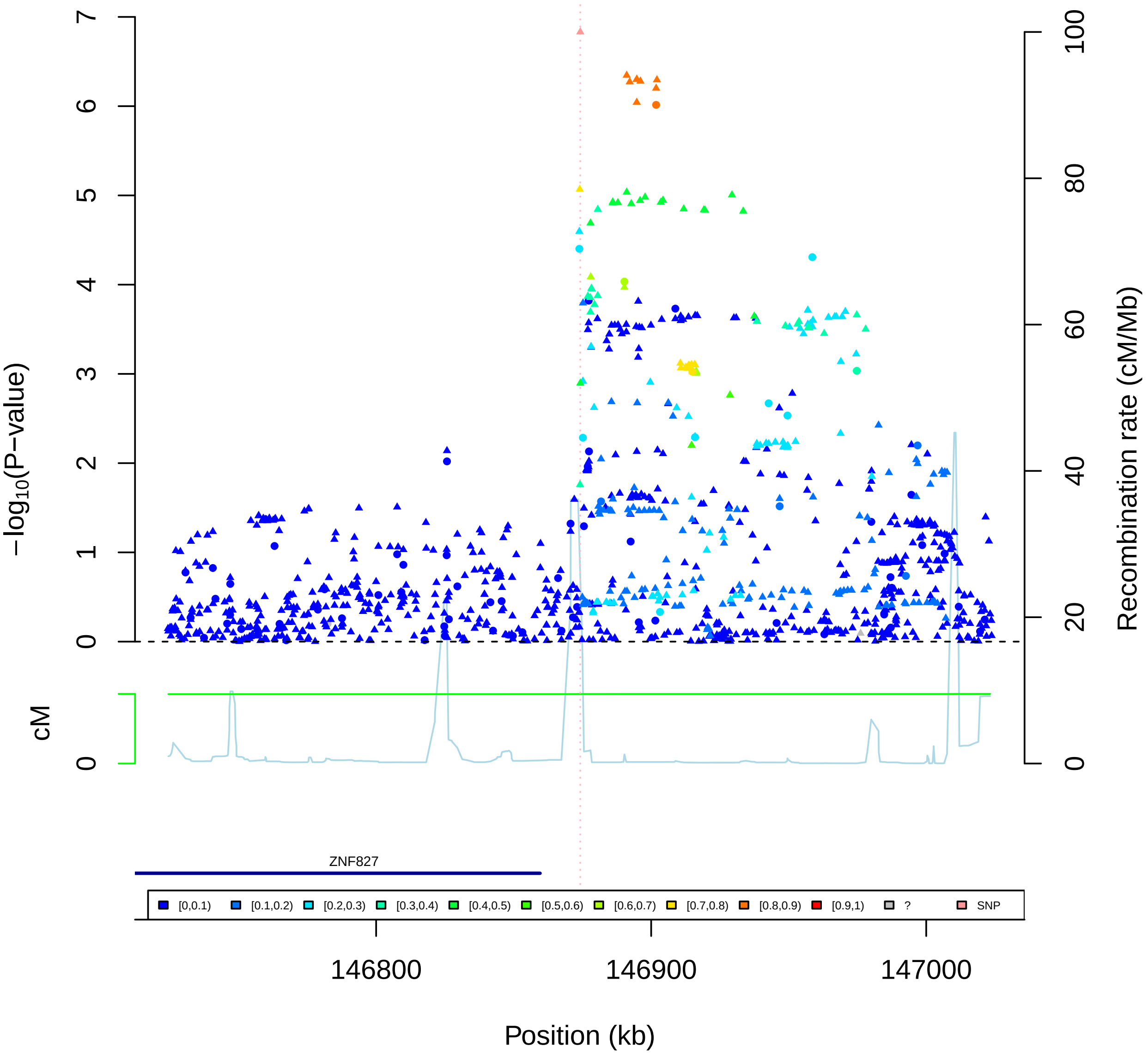

Supplementary Figure 3: Continued from previous page.

rs10023685

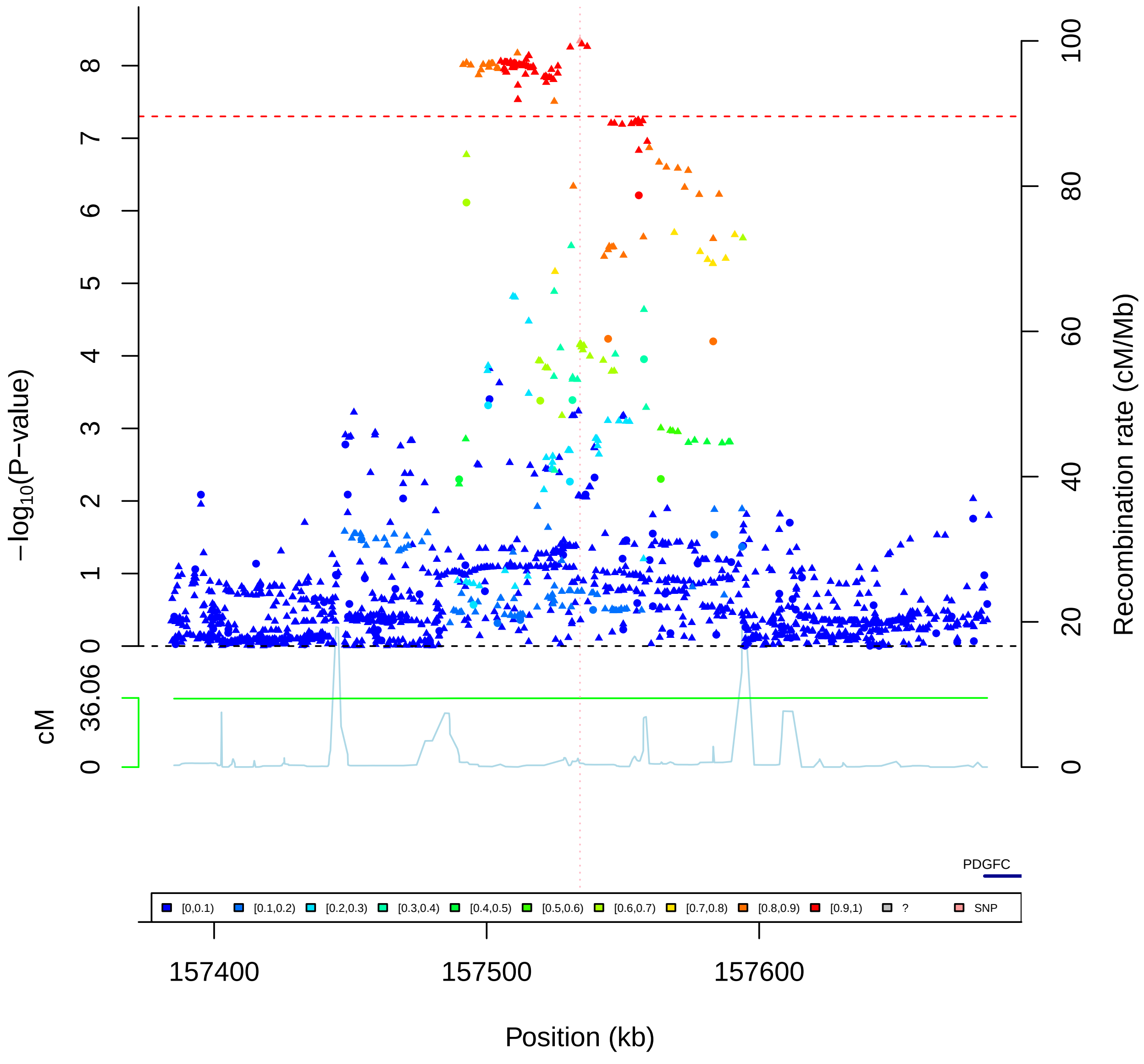

Supplementary Figure 3: Continued from previous page.

rs37004

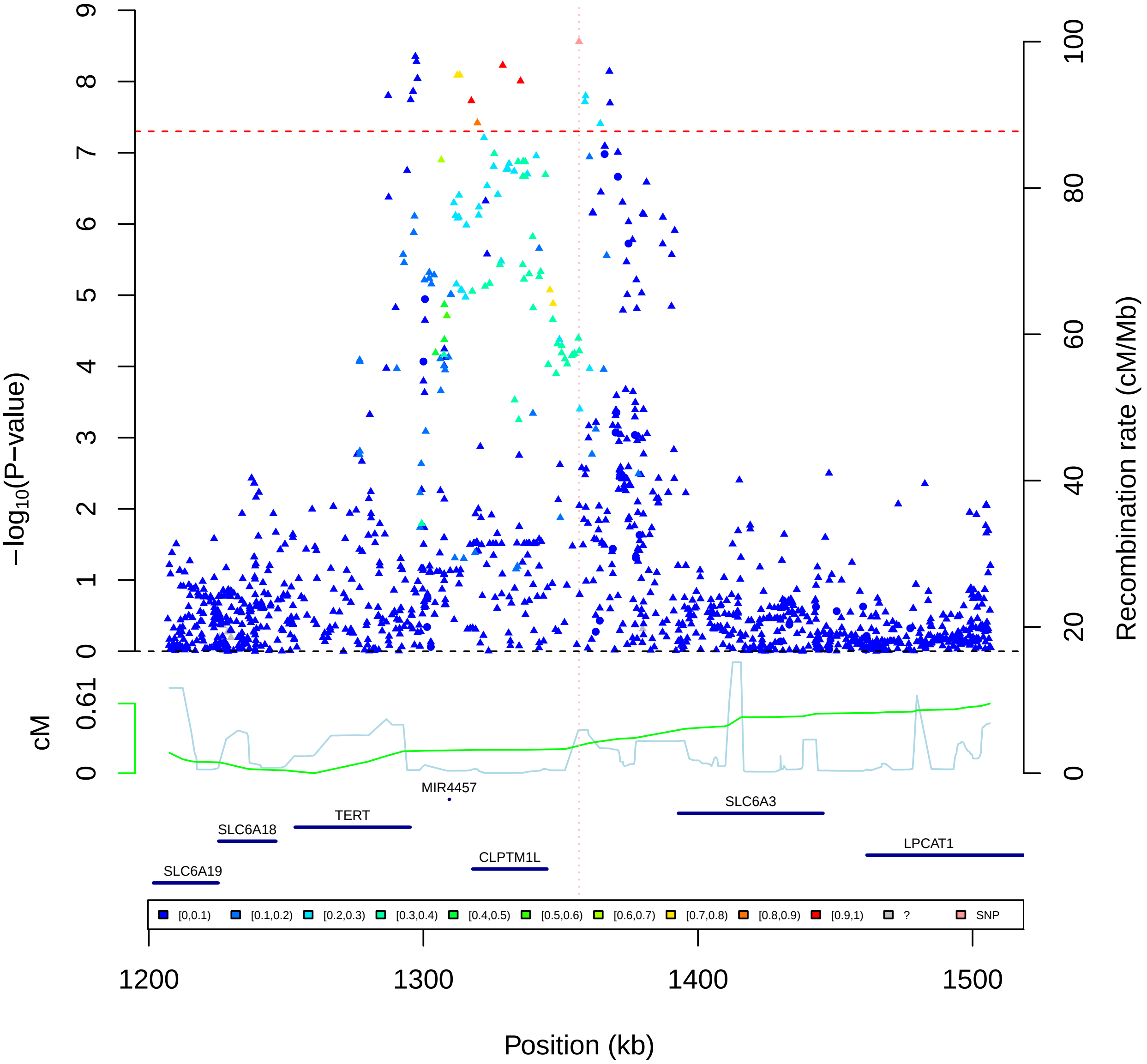

Supplementary Figure 3: Continued from previous page.

rs6920449

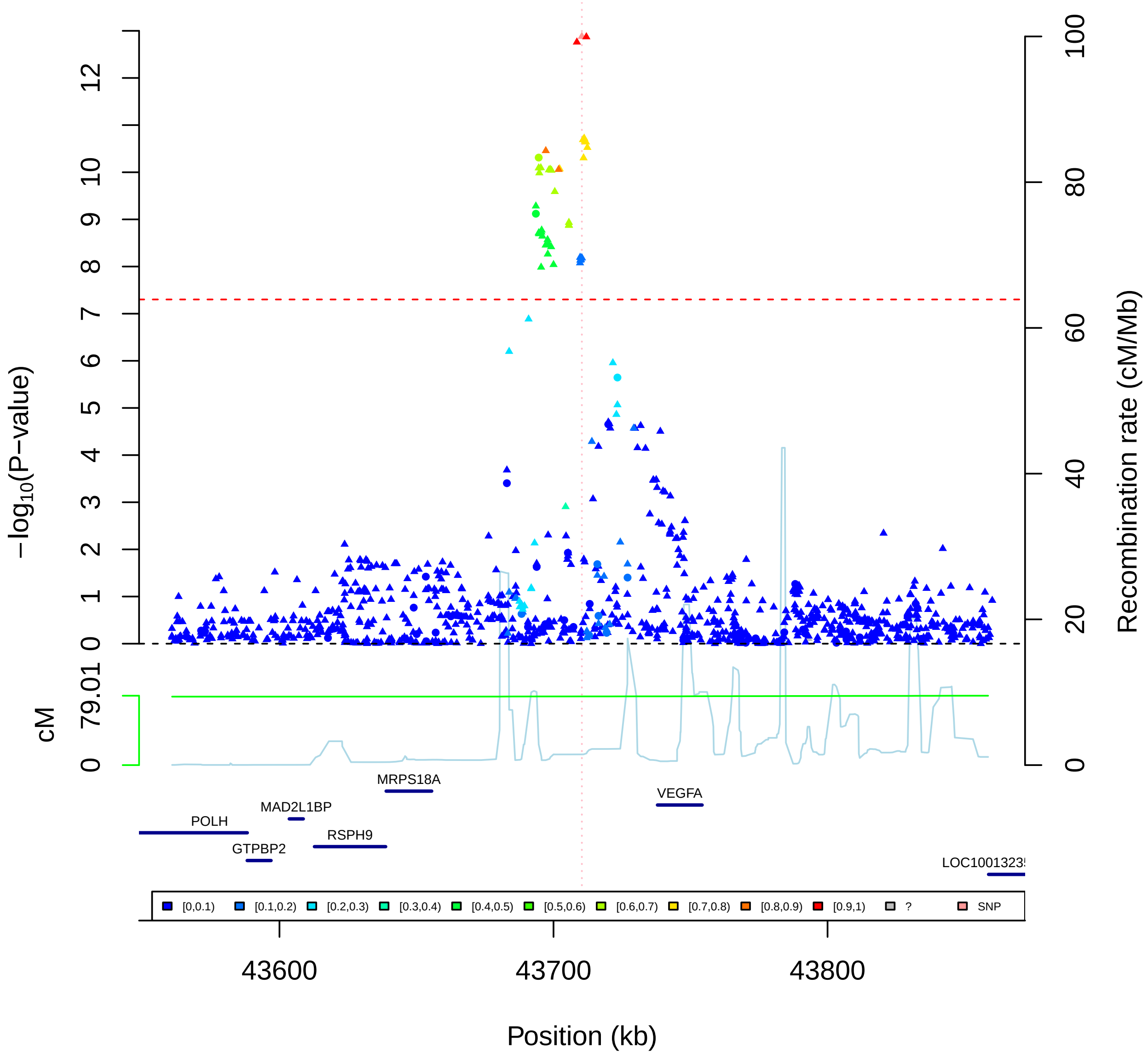

Supplementary Figure 3: Continued from previous page.

rs10486567

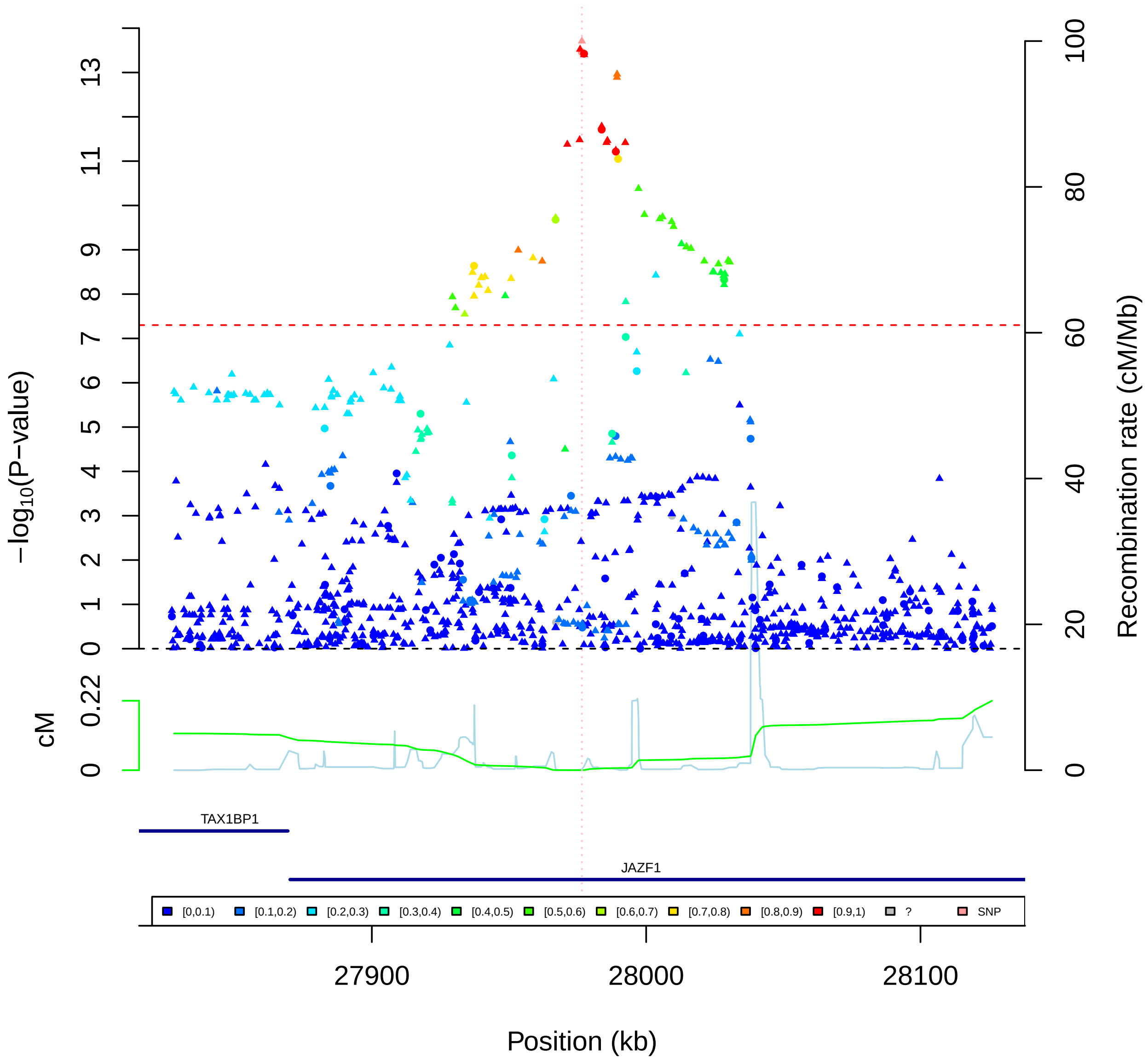

Supplementary Figure 3: Continued from previous page.

rs4614003 (Conditional round 1)

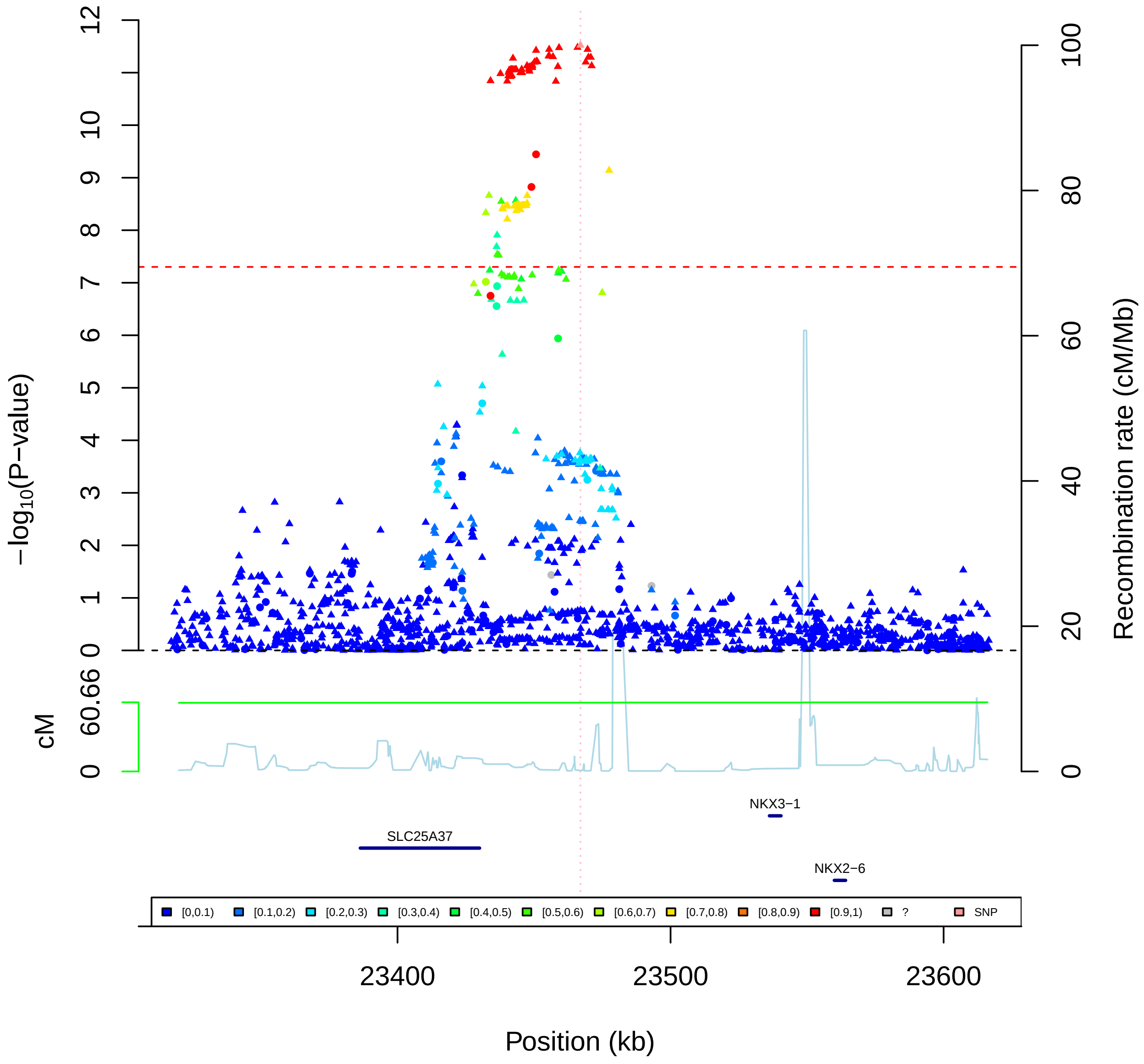

Supplementary Figure 3: Continued from previous page.

rs13272392

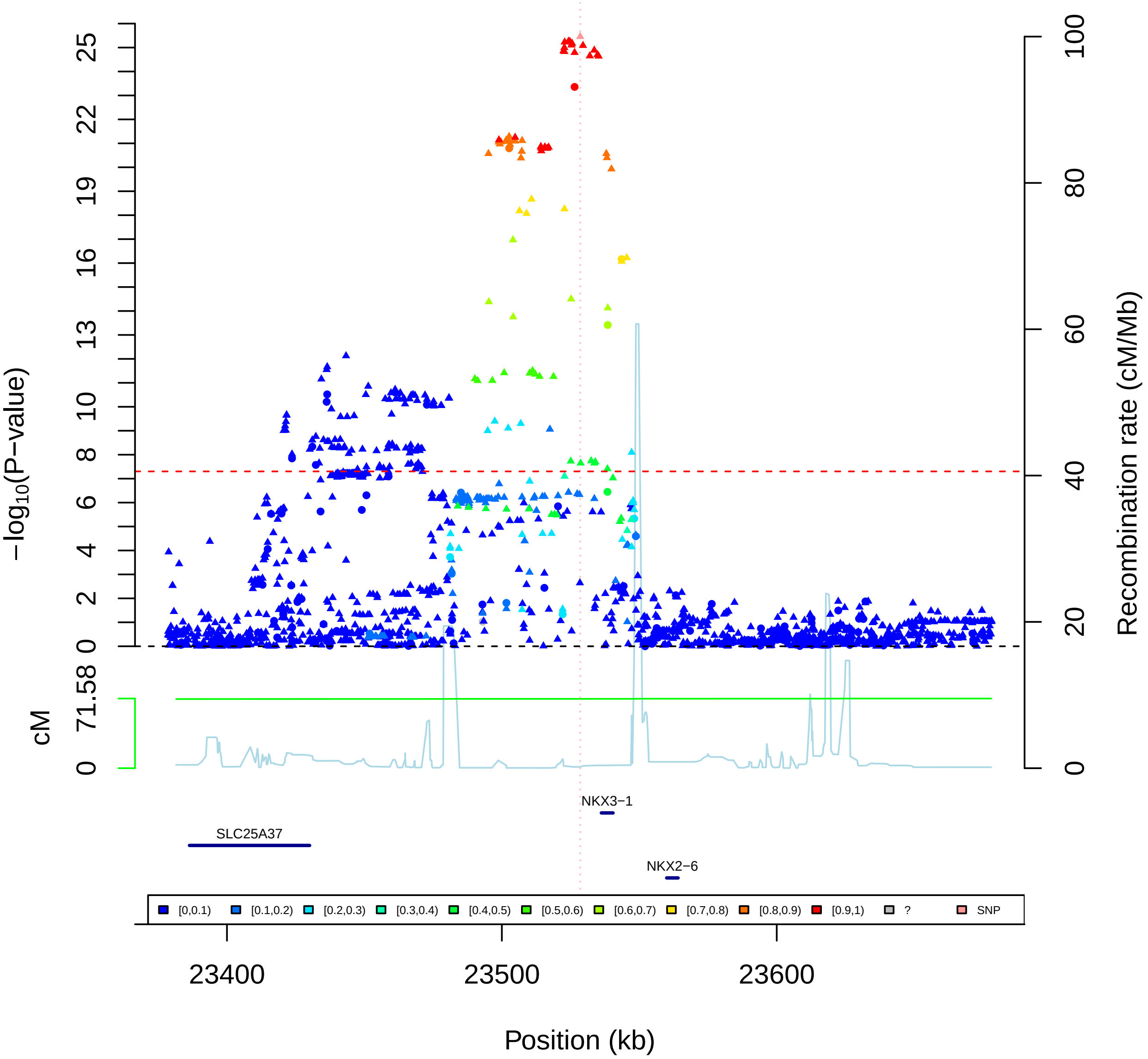

Supplementary Figure 3: Continued from previous page.

rs17464492 (Conditional round 1)

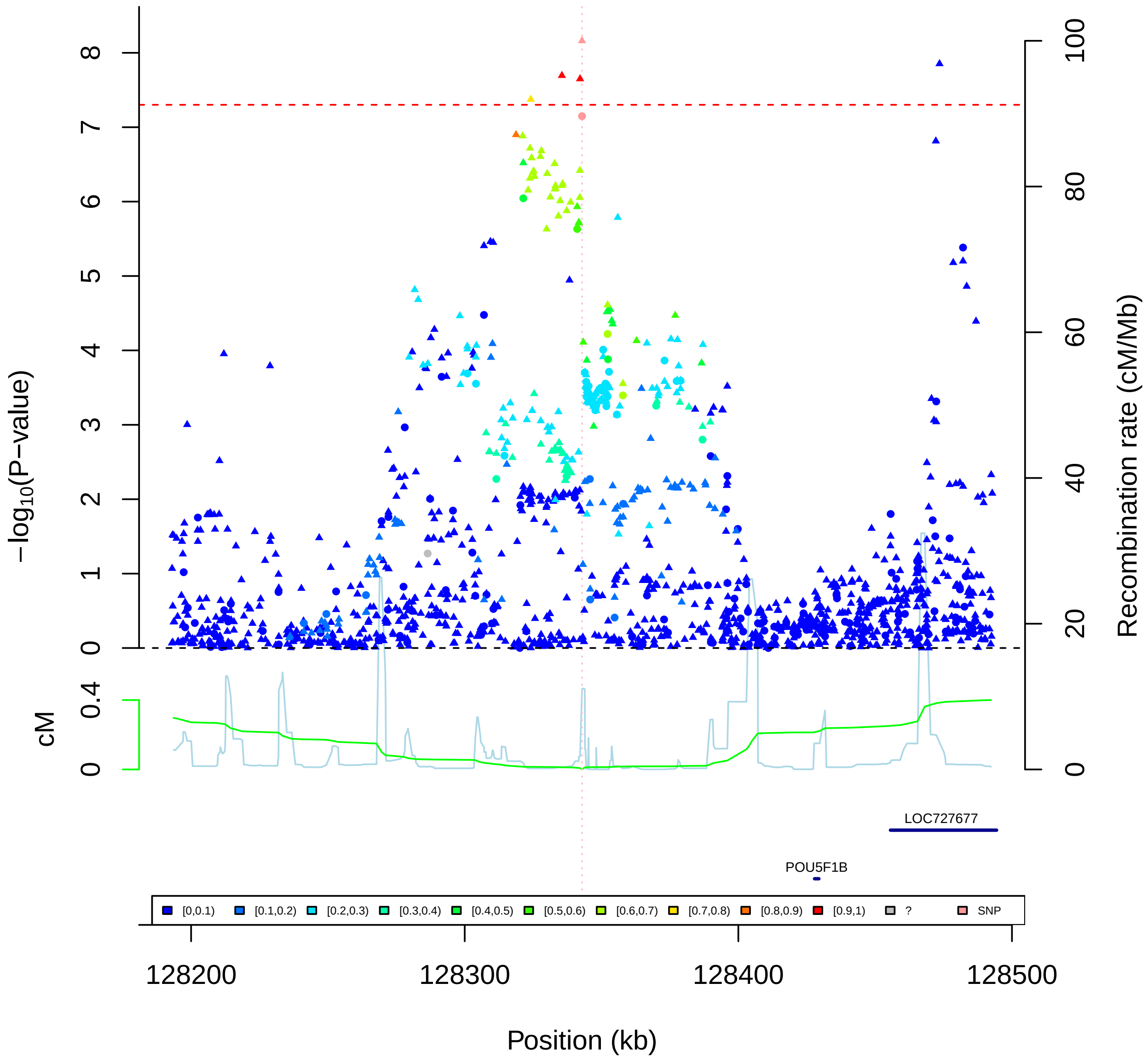

Supplementary Figure 3: Continued from previous page.

rs10505477

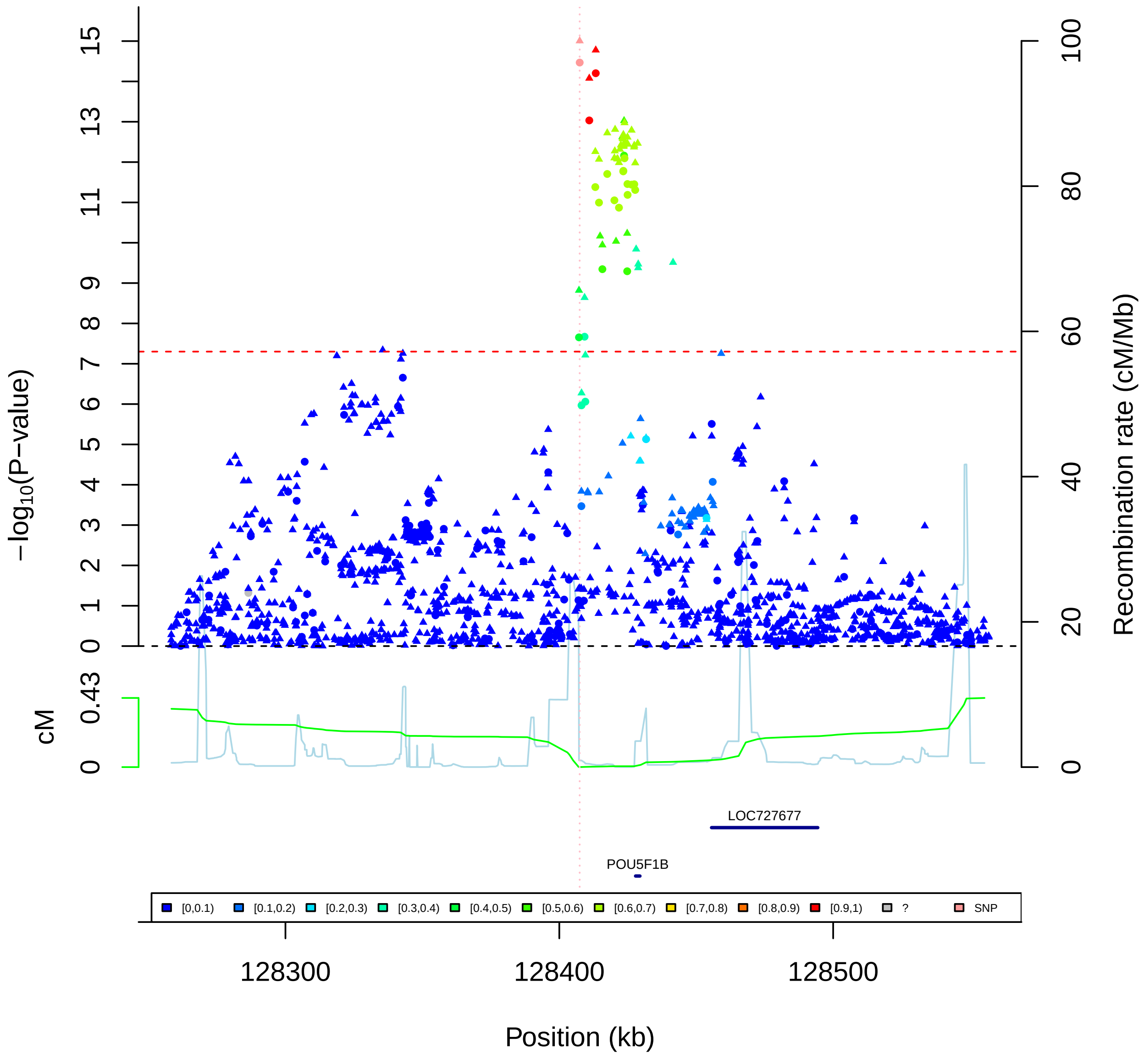

Supplementary Figure 3: Continued from previous page.

rs6478343

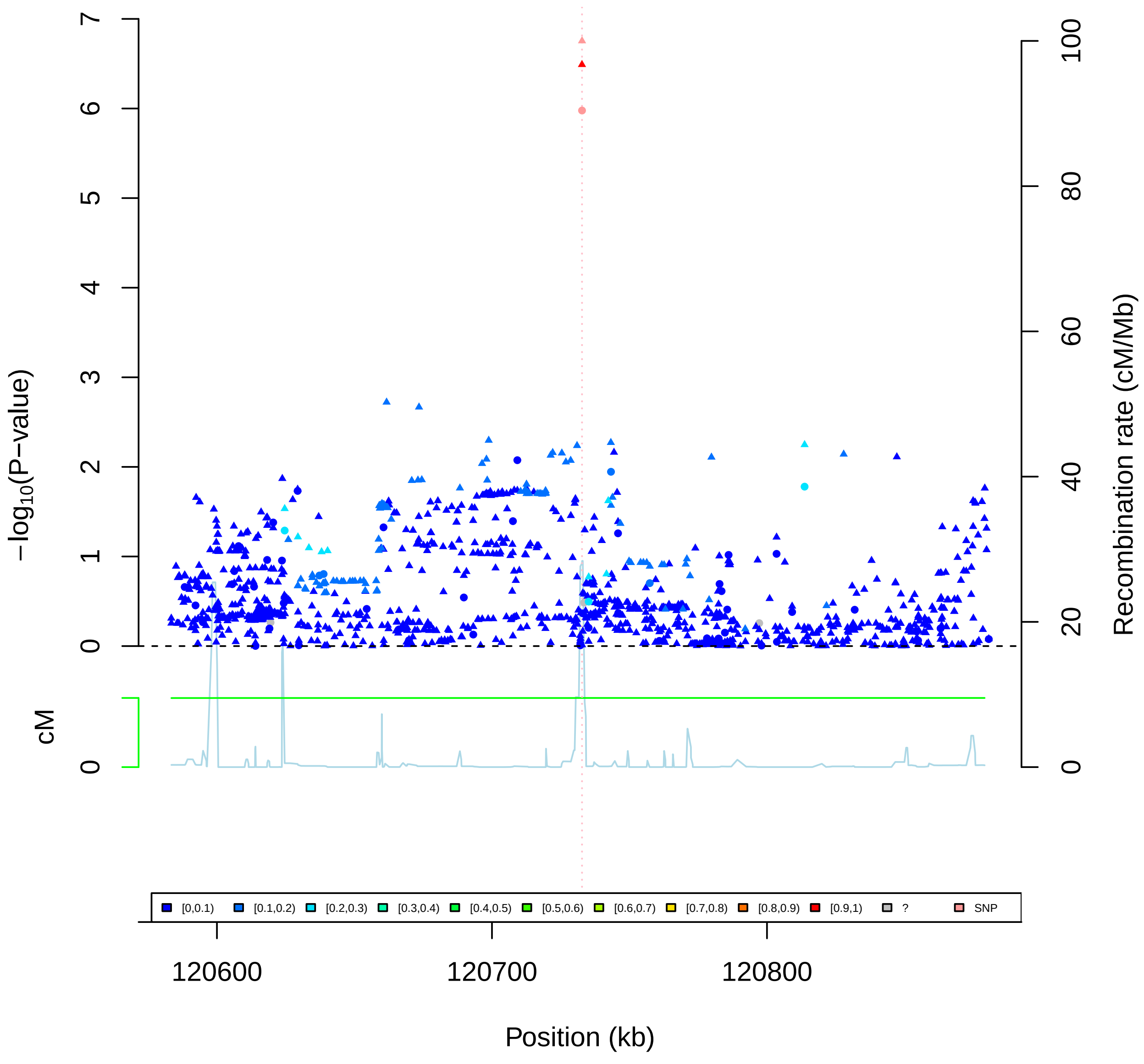

Supplementary Figure 3: Continued from previous page.

rs59482735

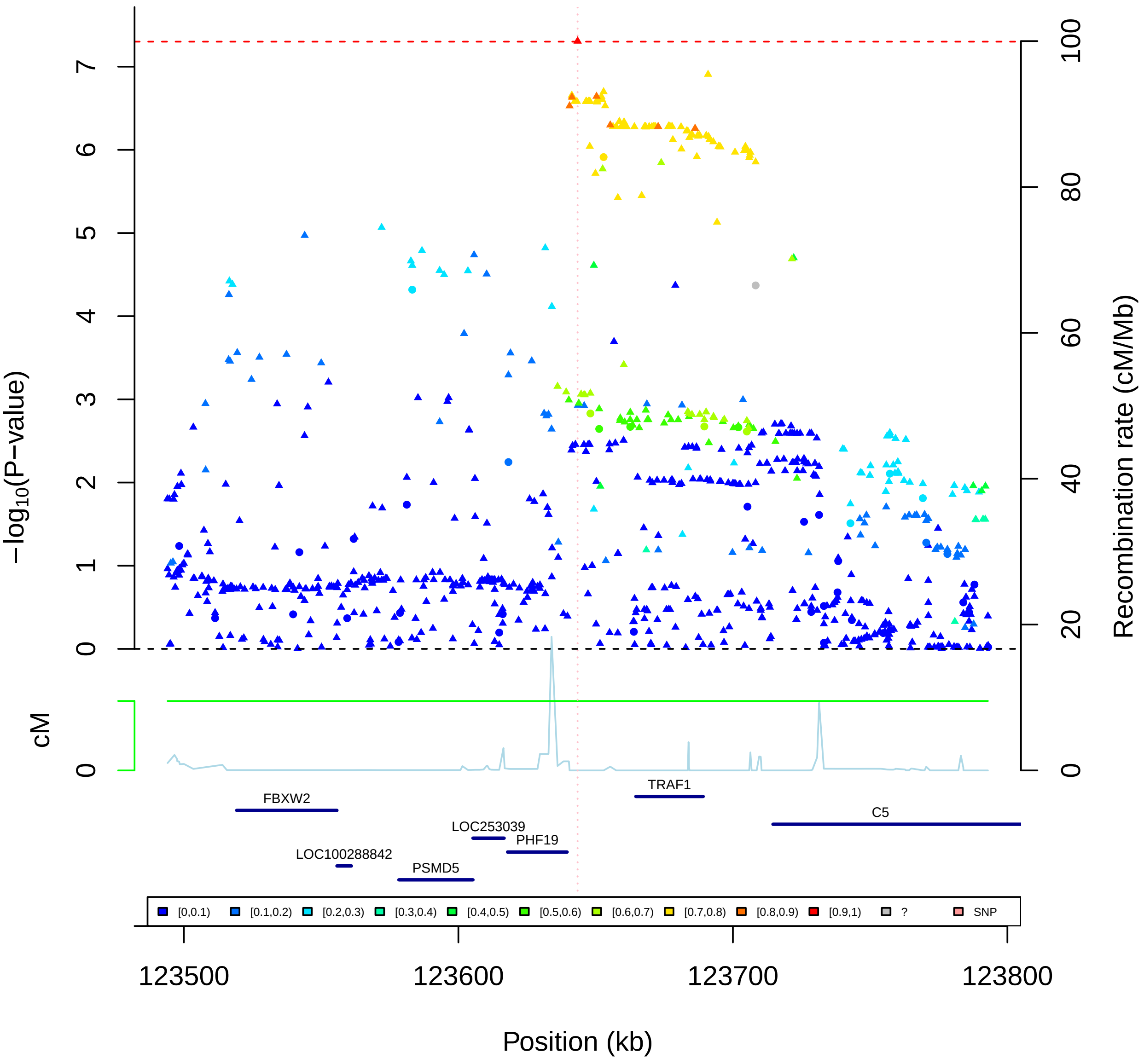

Supplementary Figure 3: Continued from previous page.

rs2492906 (Conditional round 1)

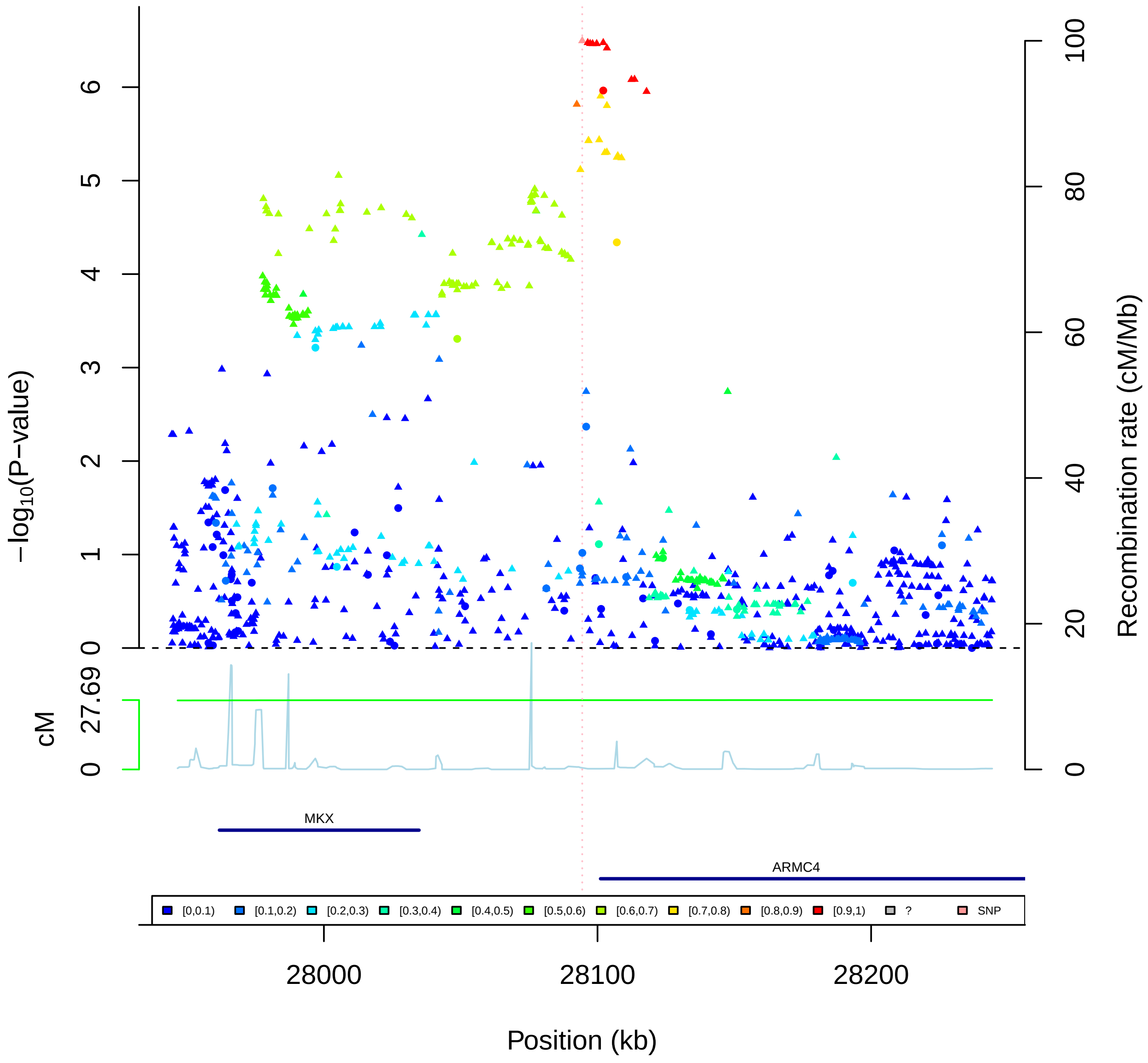

Supplementary Figure 3: Continued from previous page.

rs116940348

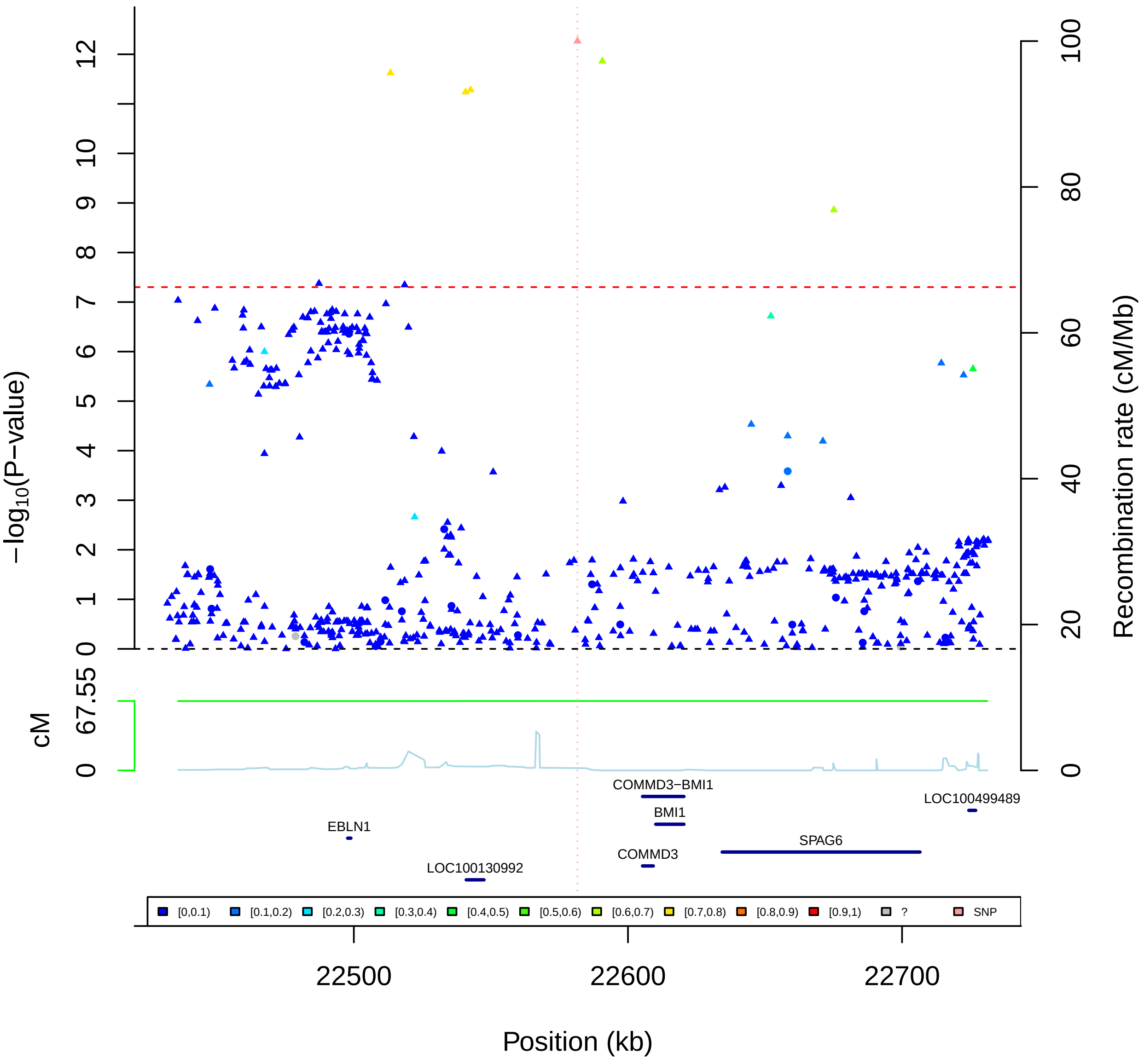

Supplementary Figure 3: Continued from previous page.

rs10993994

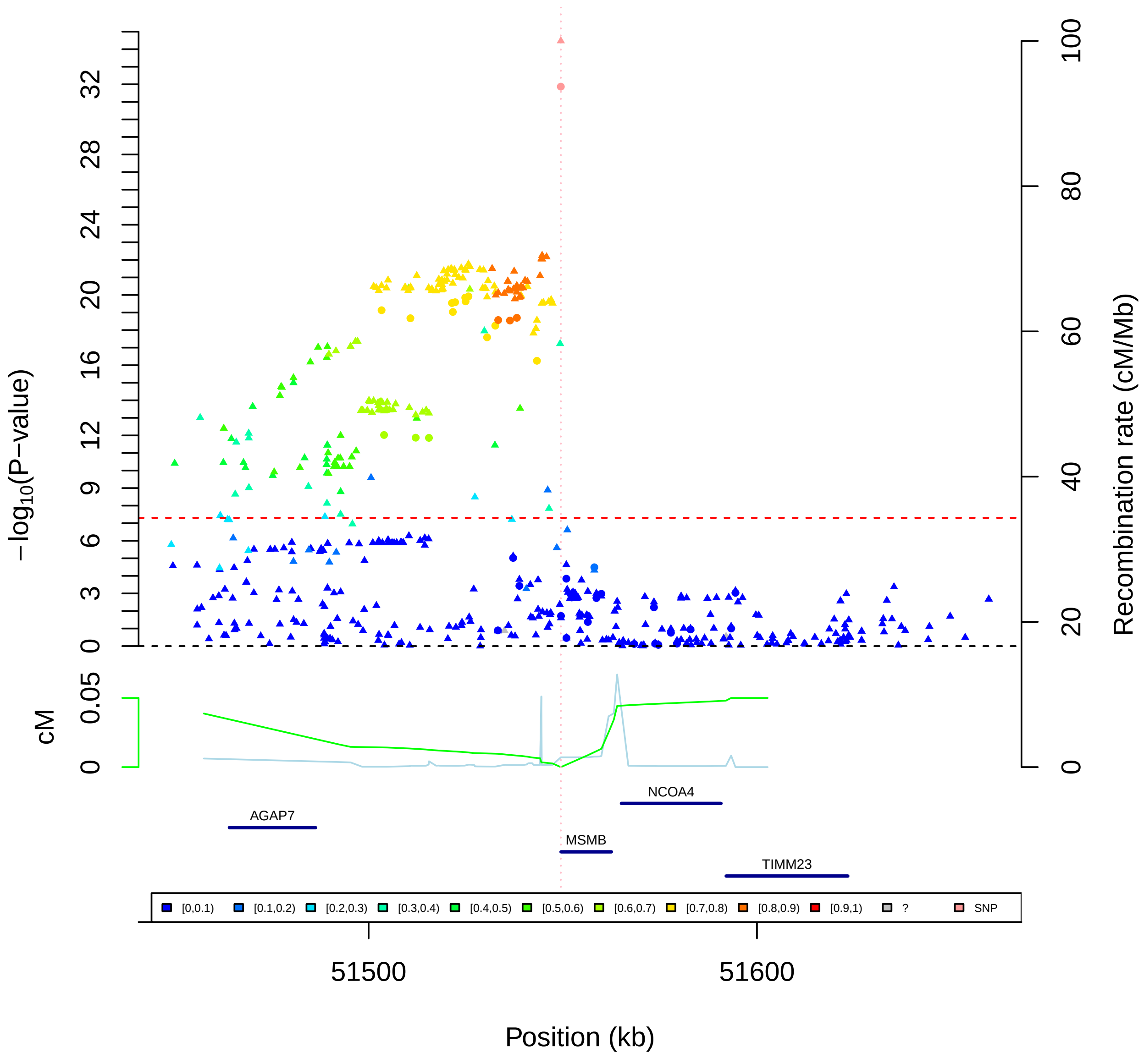

Supplementary Figure 3: Continued from previous page.

rs10886902

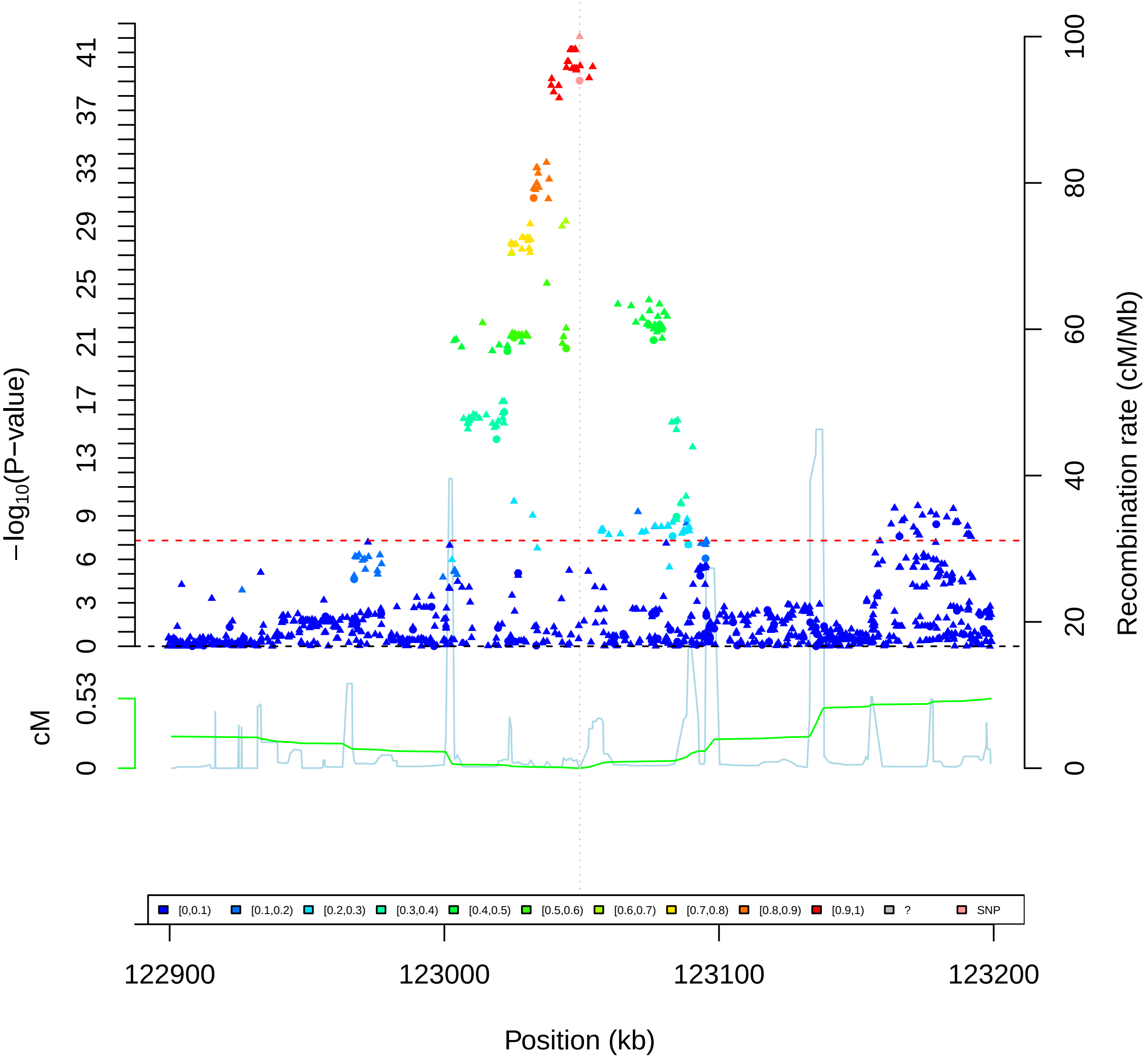

Supplementary Figure 3: Continued from previous page.

rs200367988 (Conditional round 1)

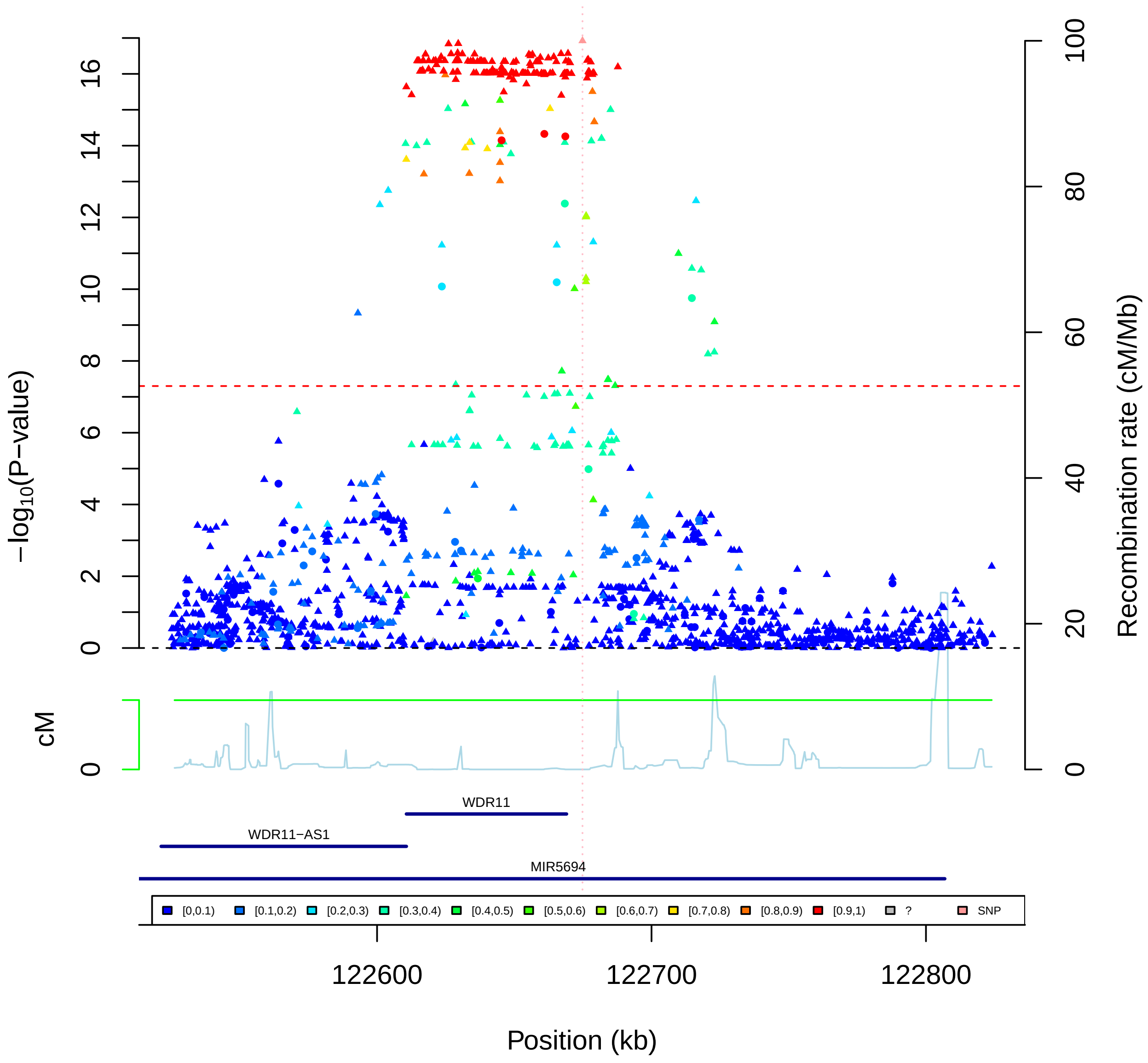

Supplementary Figure 3: Continued from previous page.

rs10749415 (Conditional round 3)

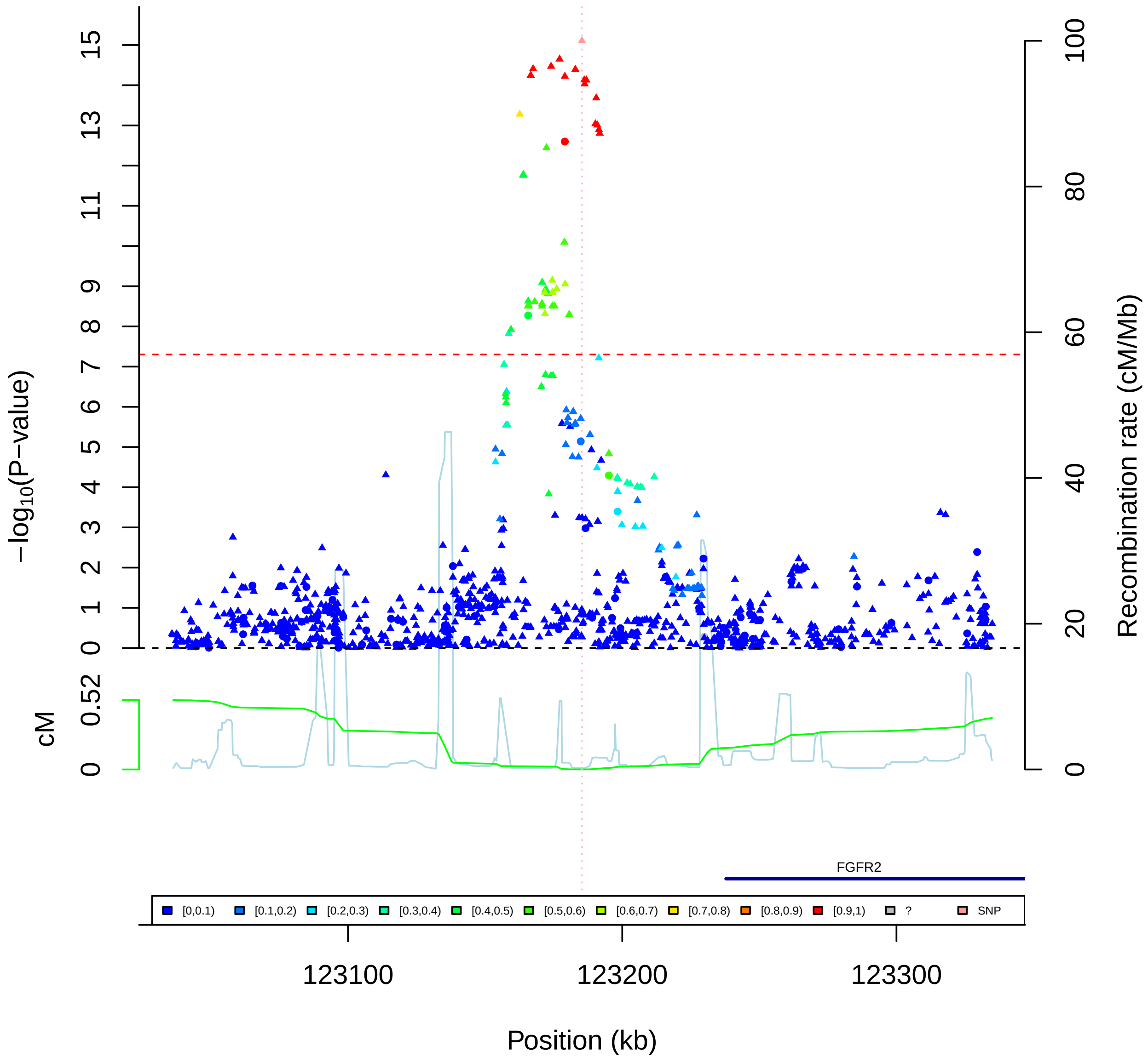

Supplementary Figure 3: Continued from previous page.

rs4378355

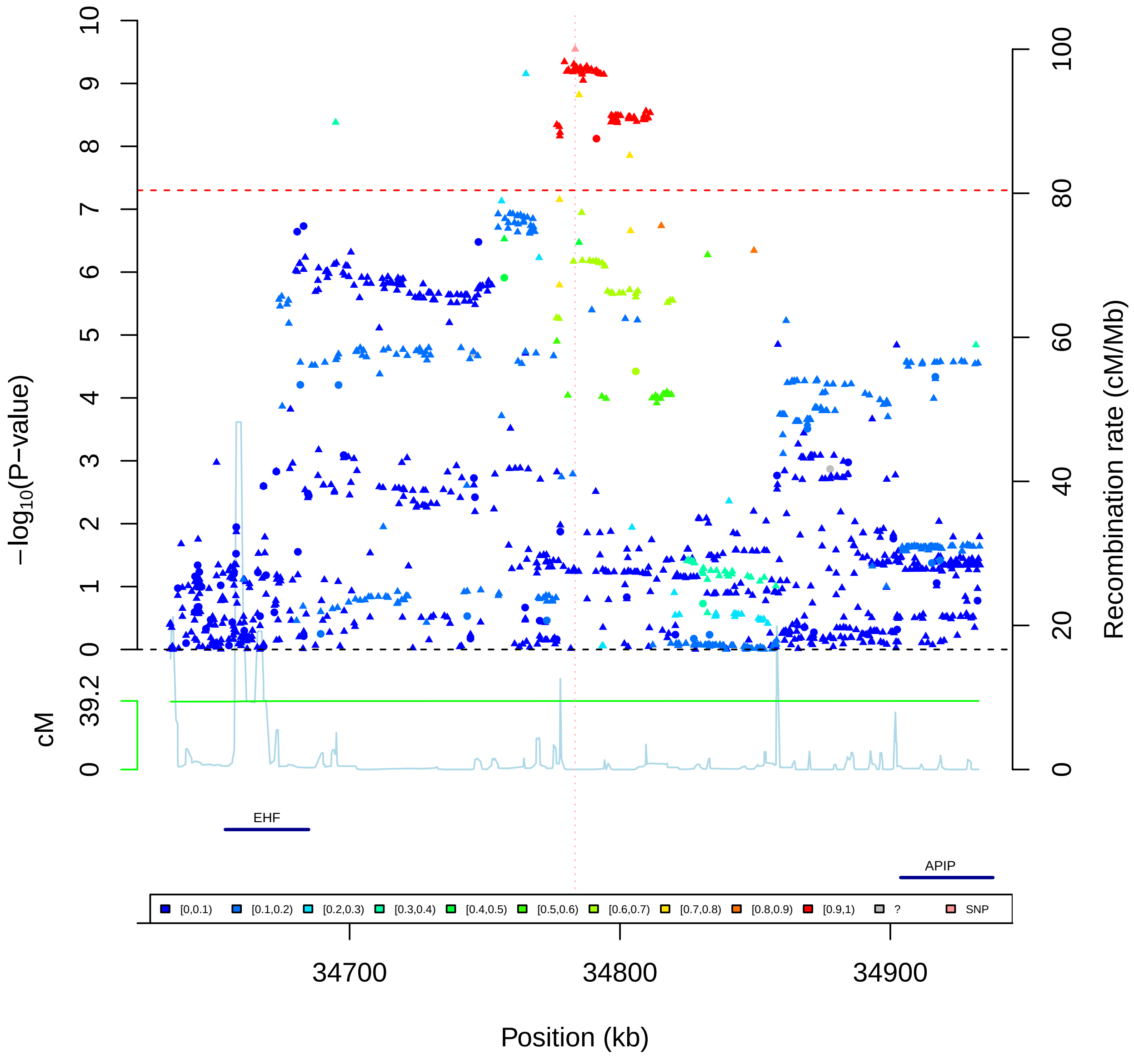

Supplementary Figure 3: Continued from previous page.

rs12285347

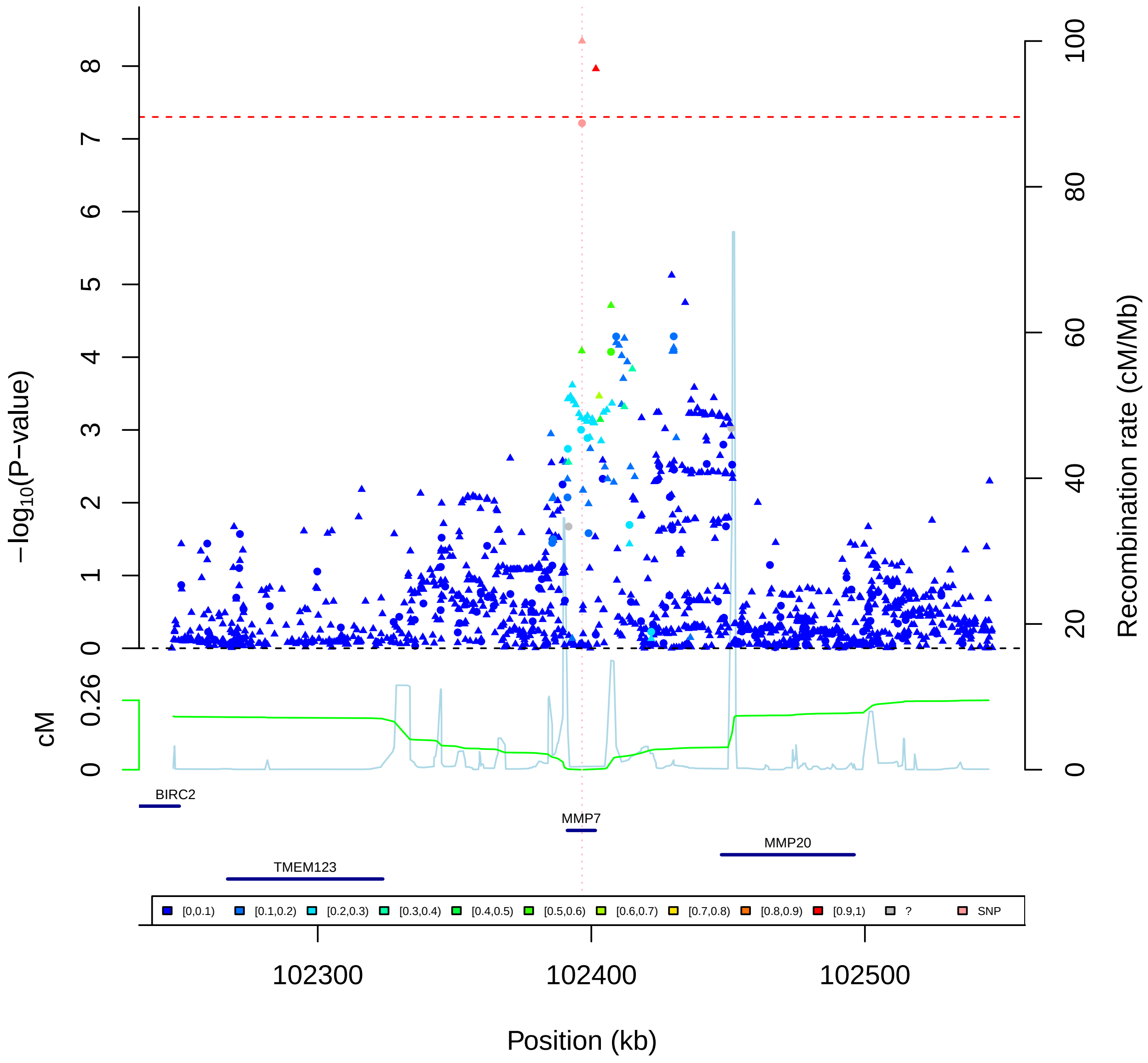

Supplementary Figure 3: Continued from previous page.

rs11067228

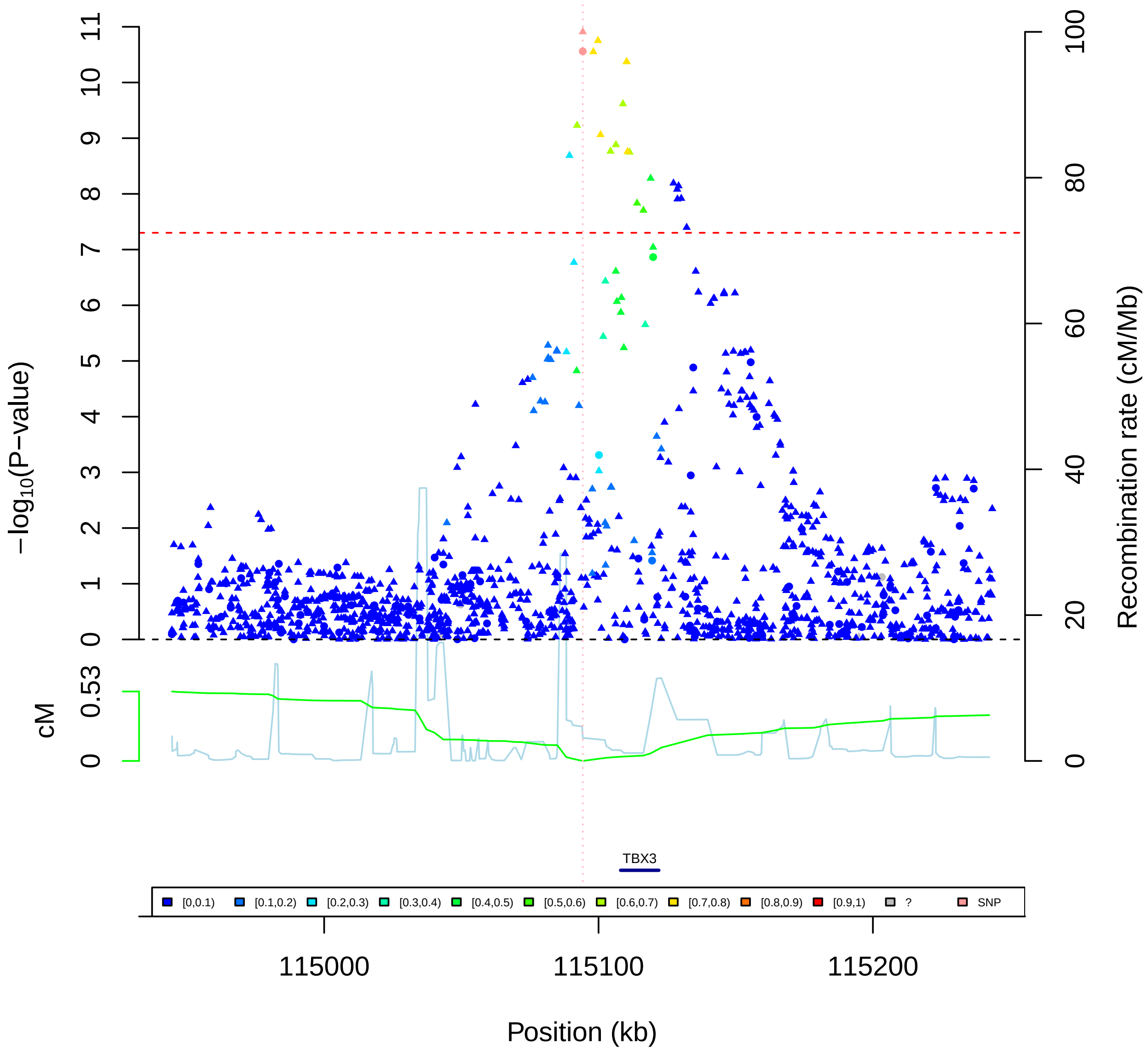

Supplementary Figure 3: Continued from previous page.

rs202346

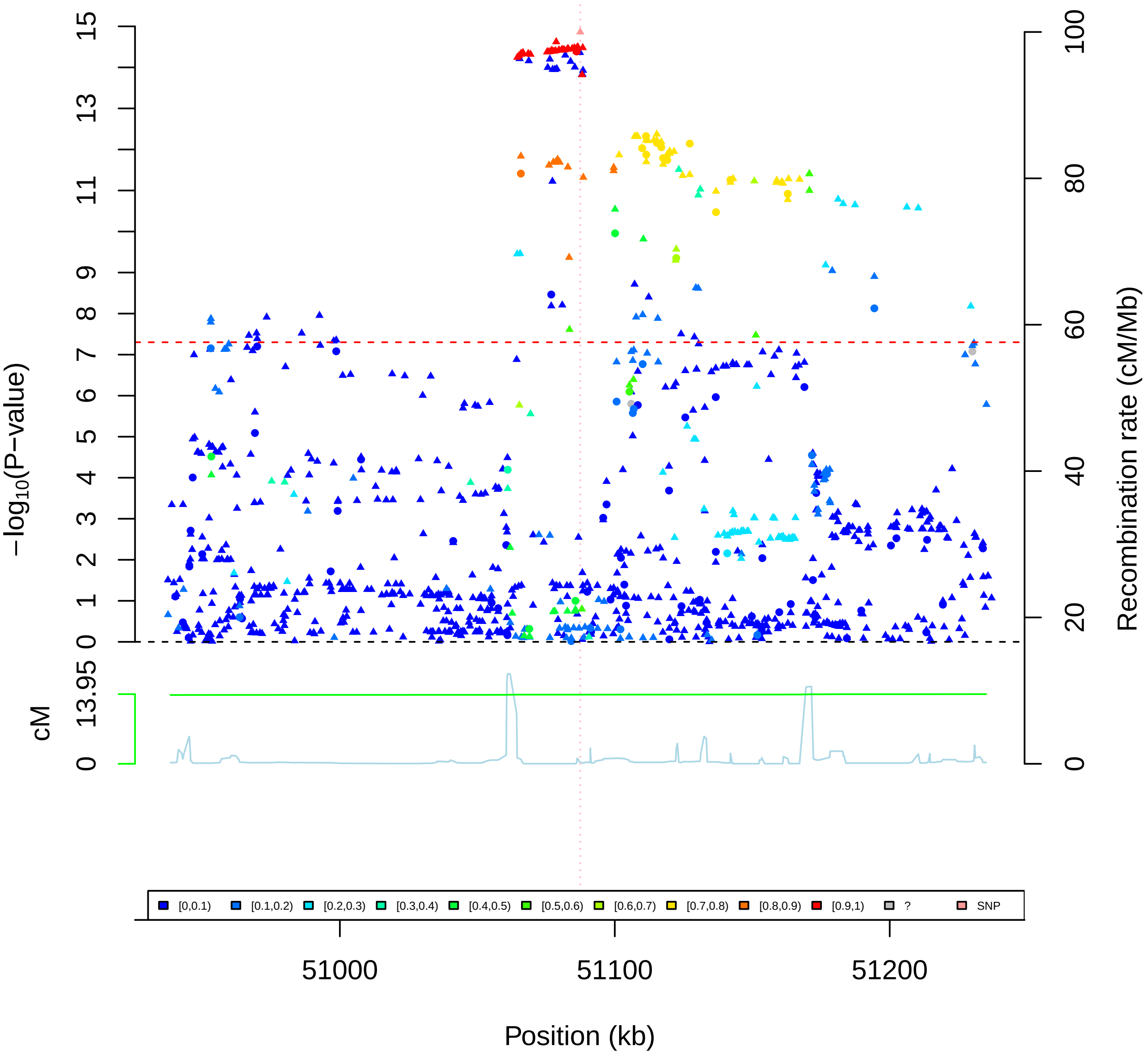

Supplementary Figure 3: Continued from previous page.

rs8023057

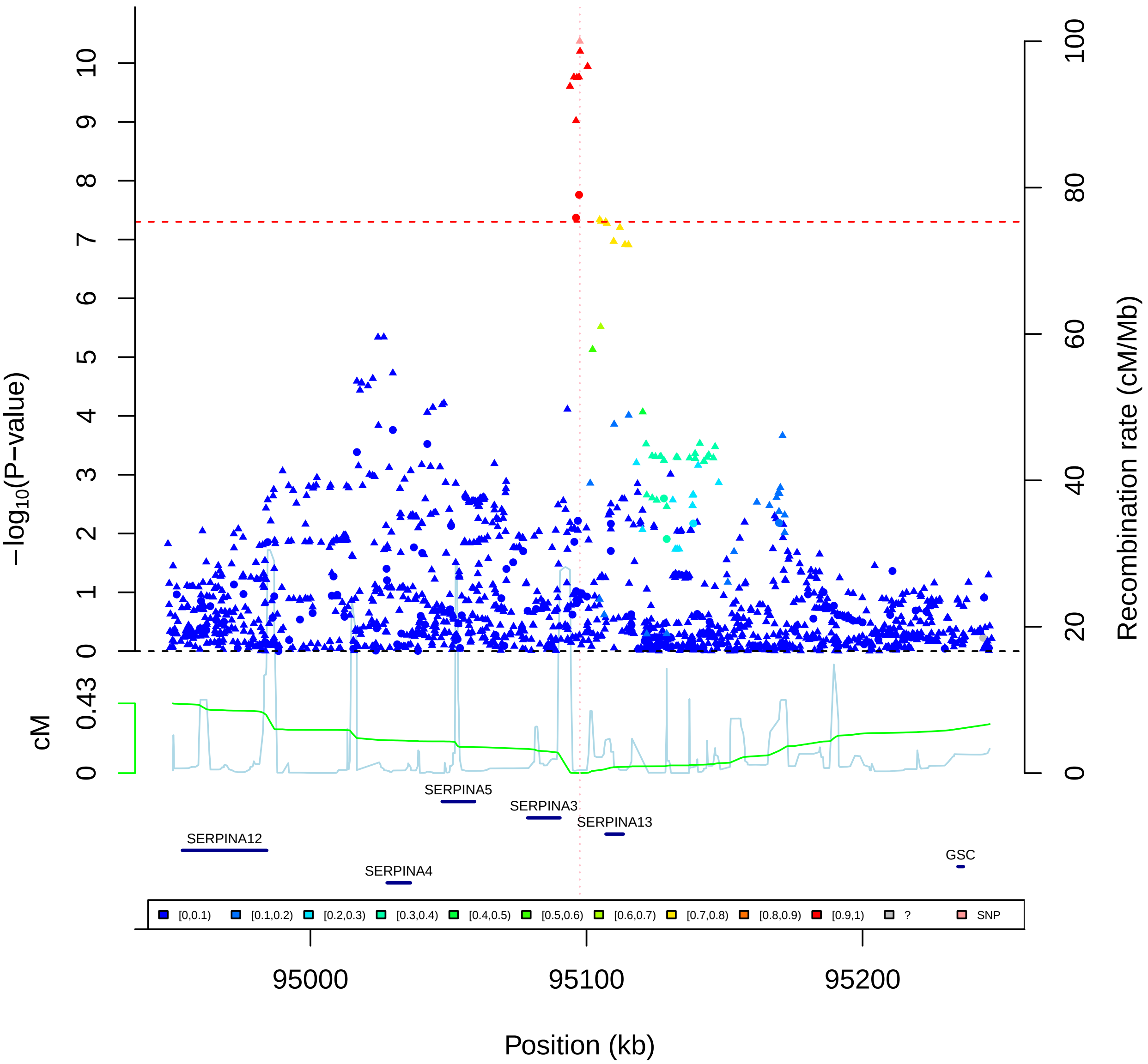

Supplementary Figure 3: Continued from previous page.

rs9921192

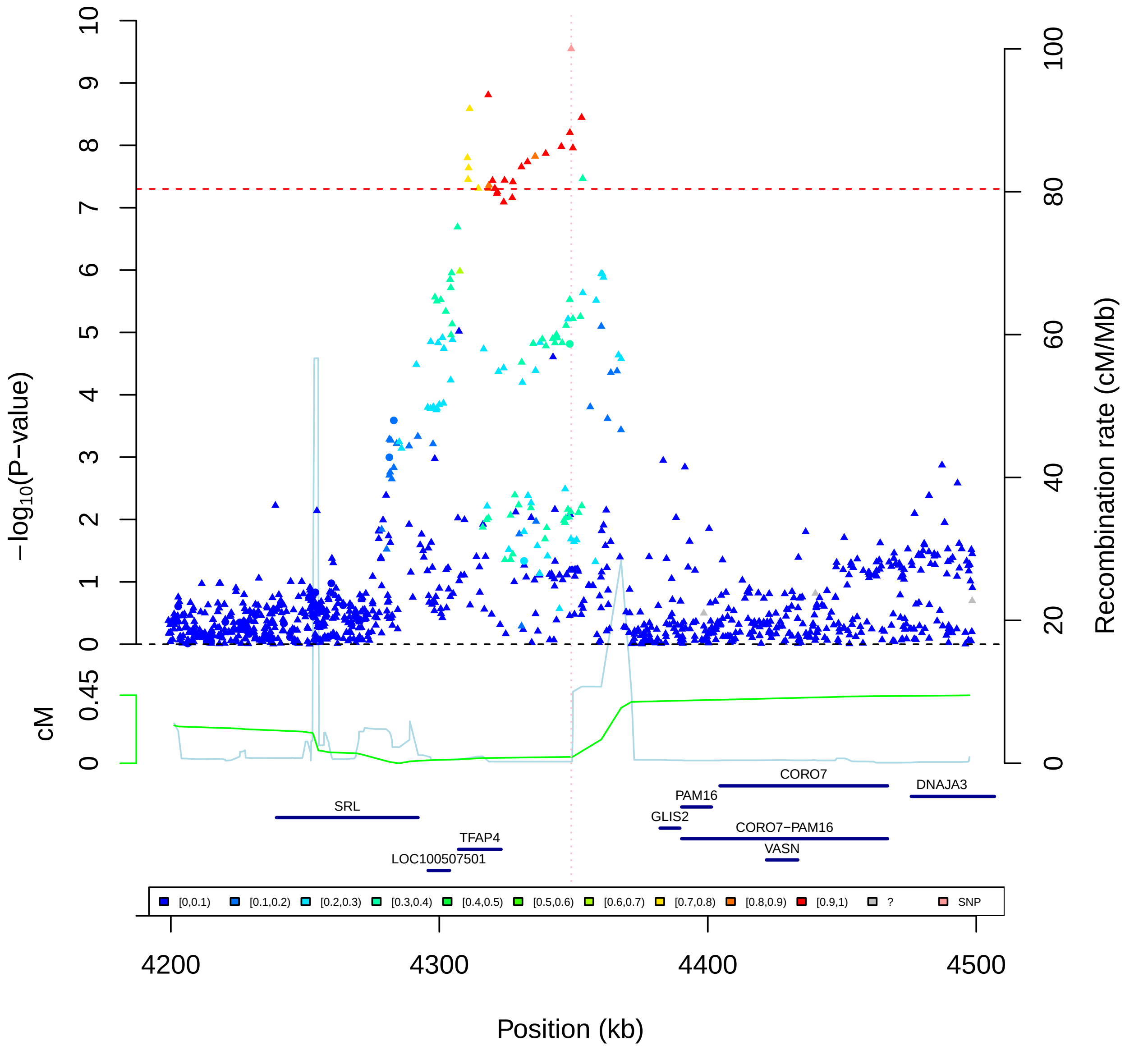

Supplementary Figure 3: Continued from previous page.

rs11263761

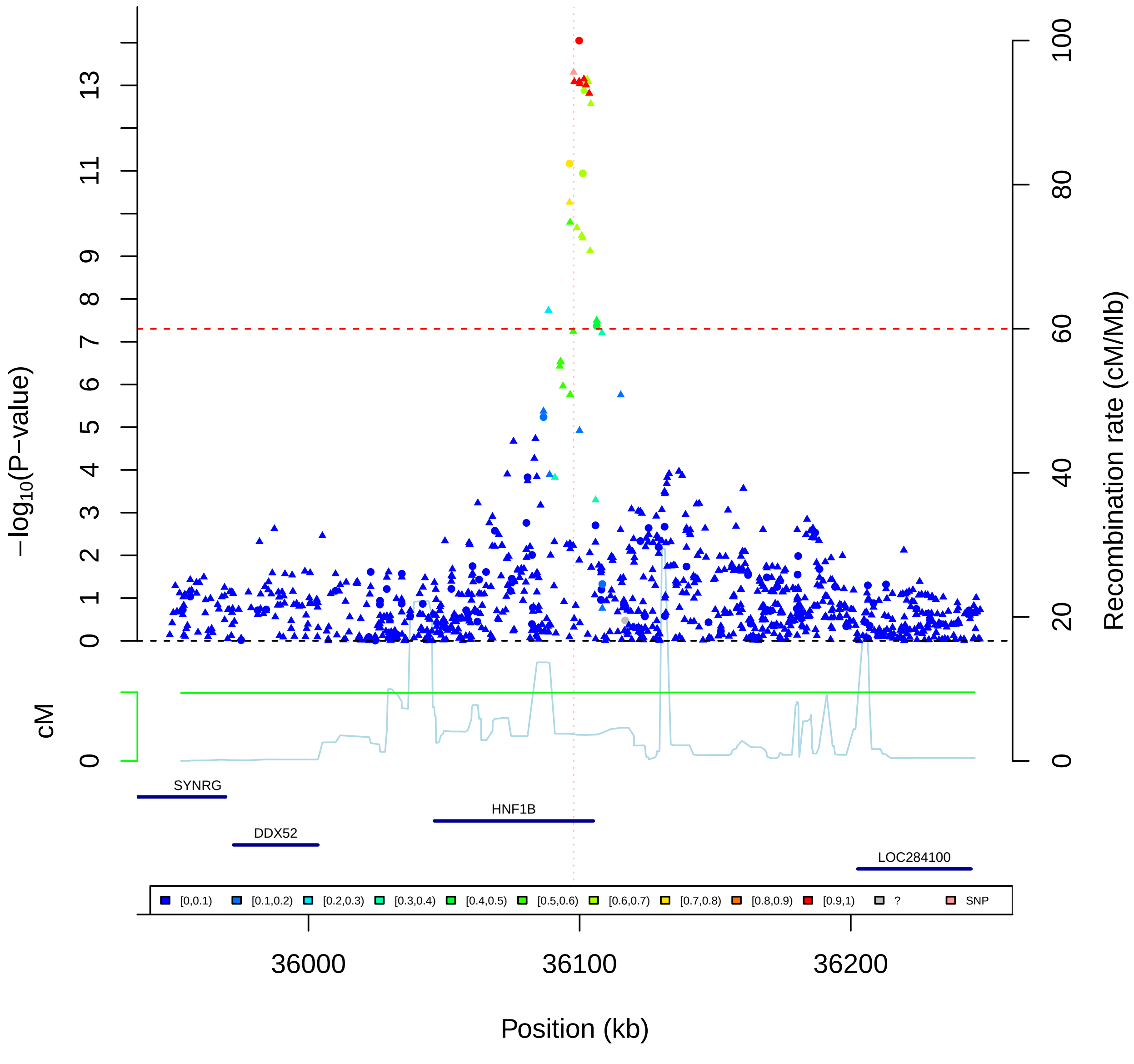

Supplementary Figure 3: Continued from previous page.

rs11084596

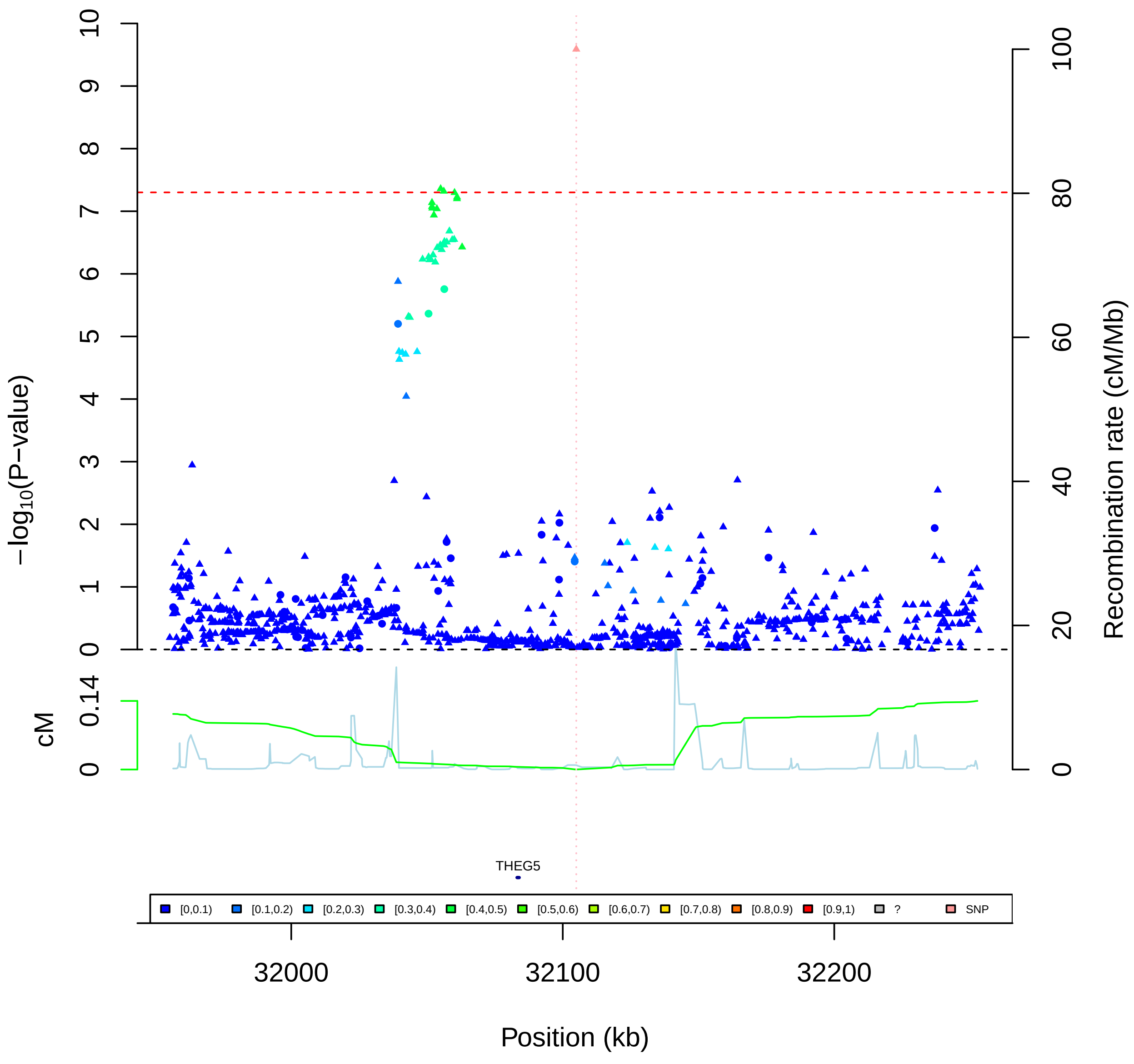

Supplementary Figure 3: Continued from previous page.

rs266849 (Conditional round 4)

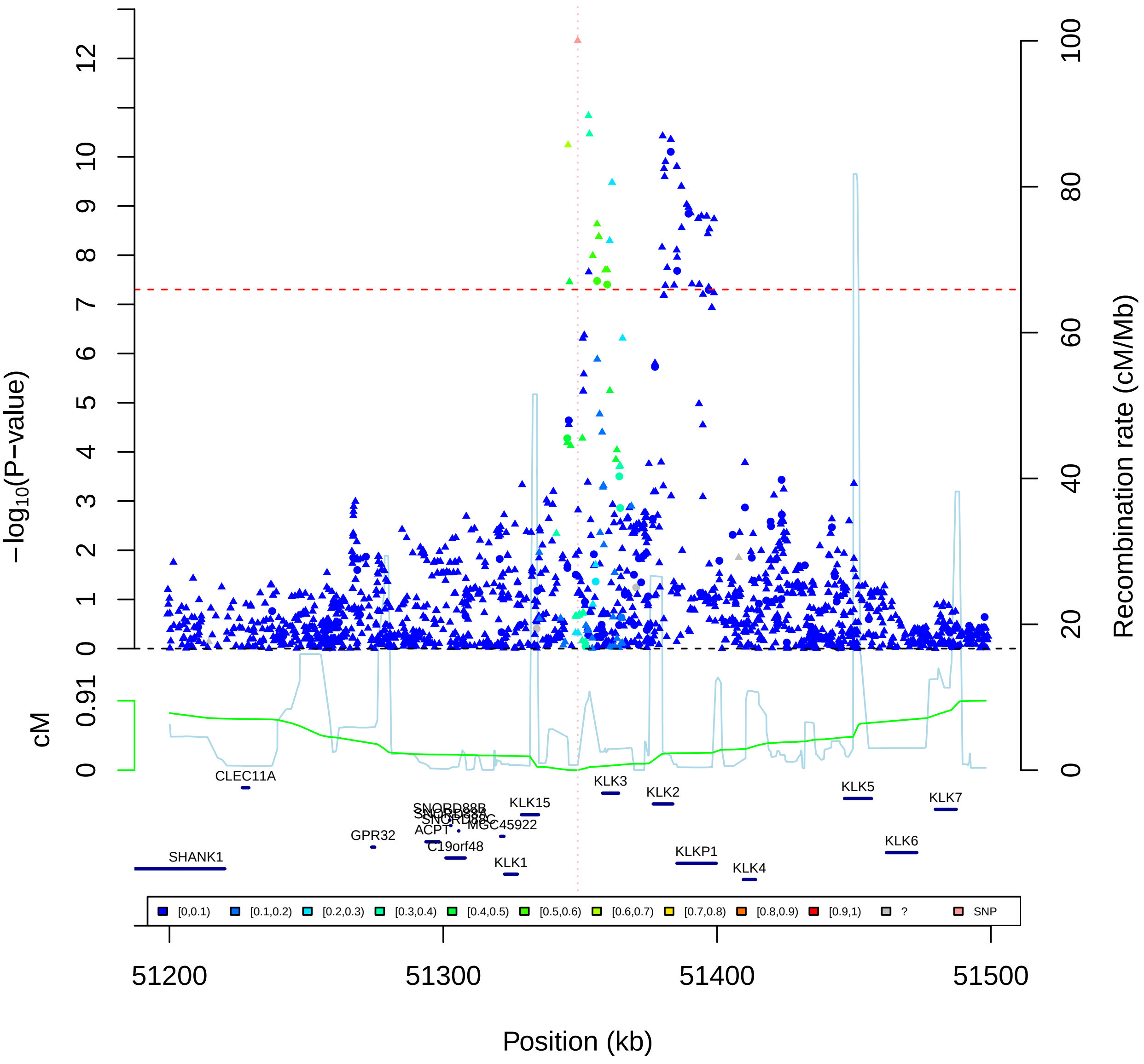

Supplementary Figure 3: Continued from previous page.

rs266868 (Conditional round 3)

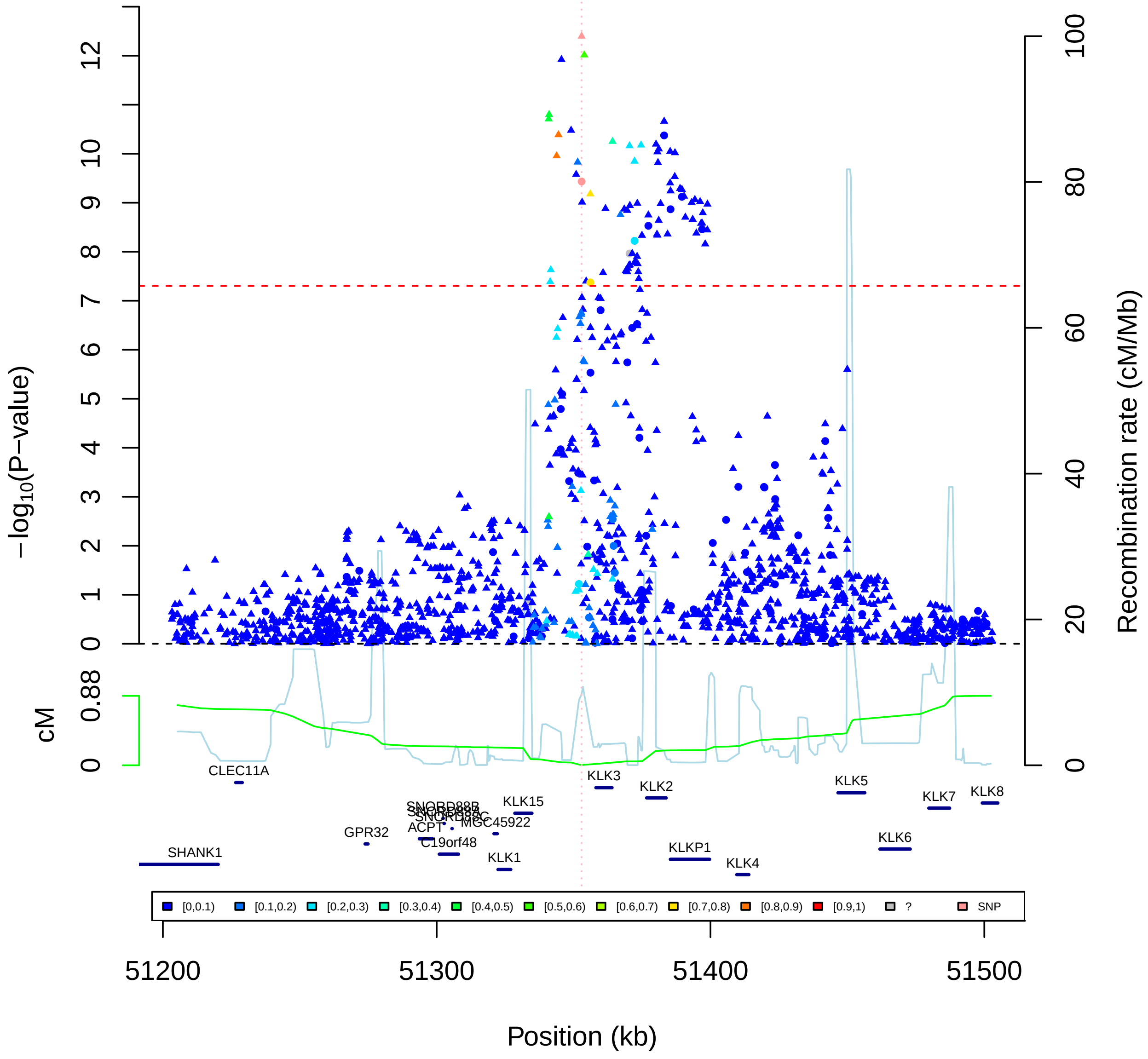

Supplementary Figure 3: Continued from previous page.

rs11665748 (Conditional round 1)

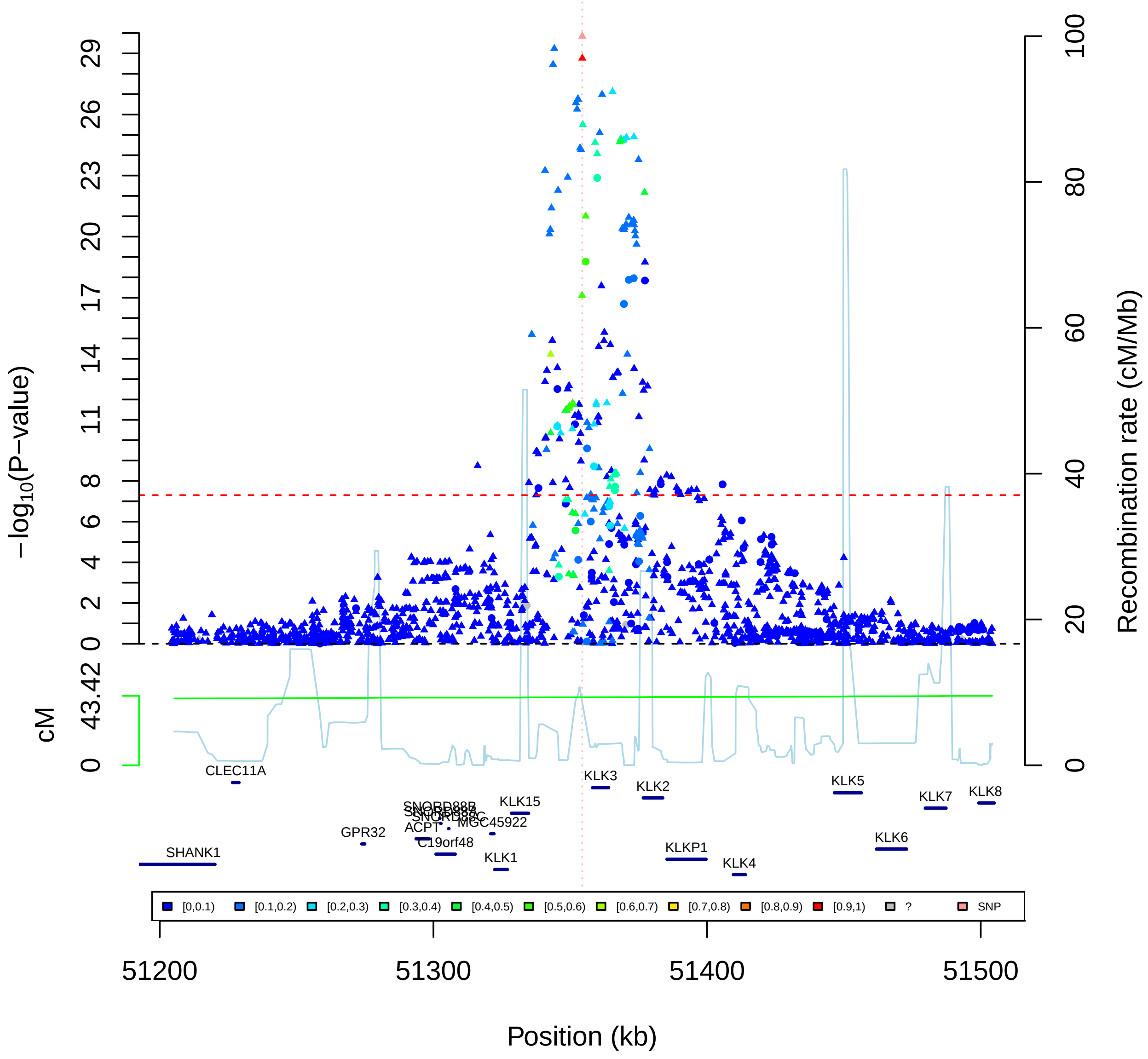

Supplementary Figure 3: Continued from previous page.

rs61752561 (Conditional round 2)

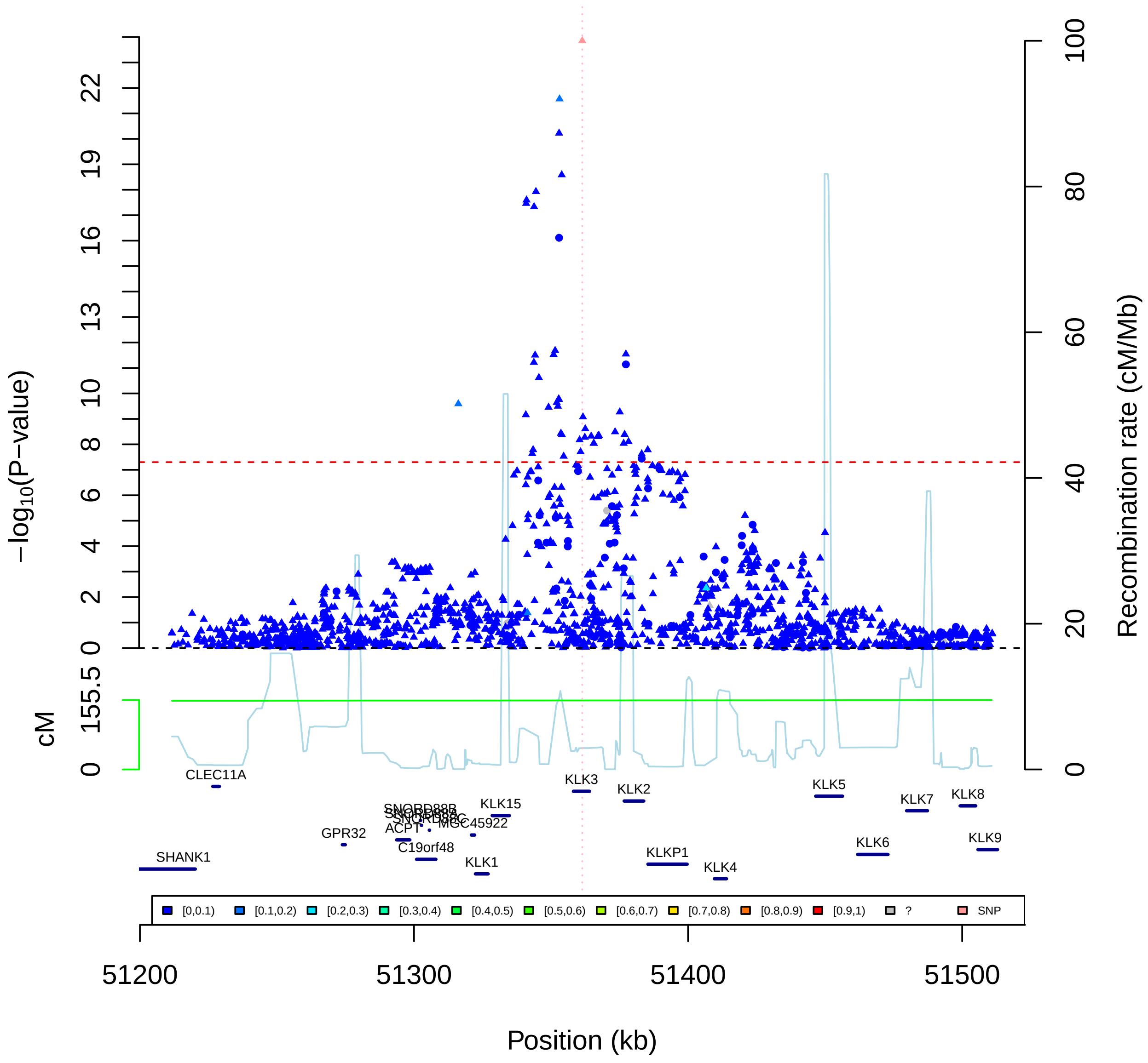

Supplementary Figure 3: Continued from previous page.

rs2739472 (Conditional round 6)

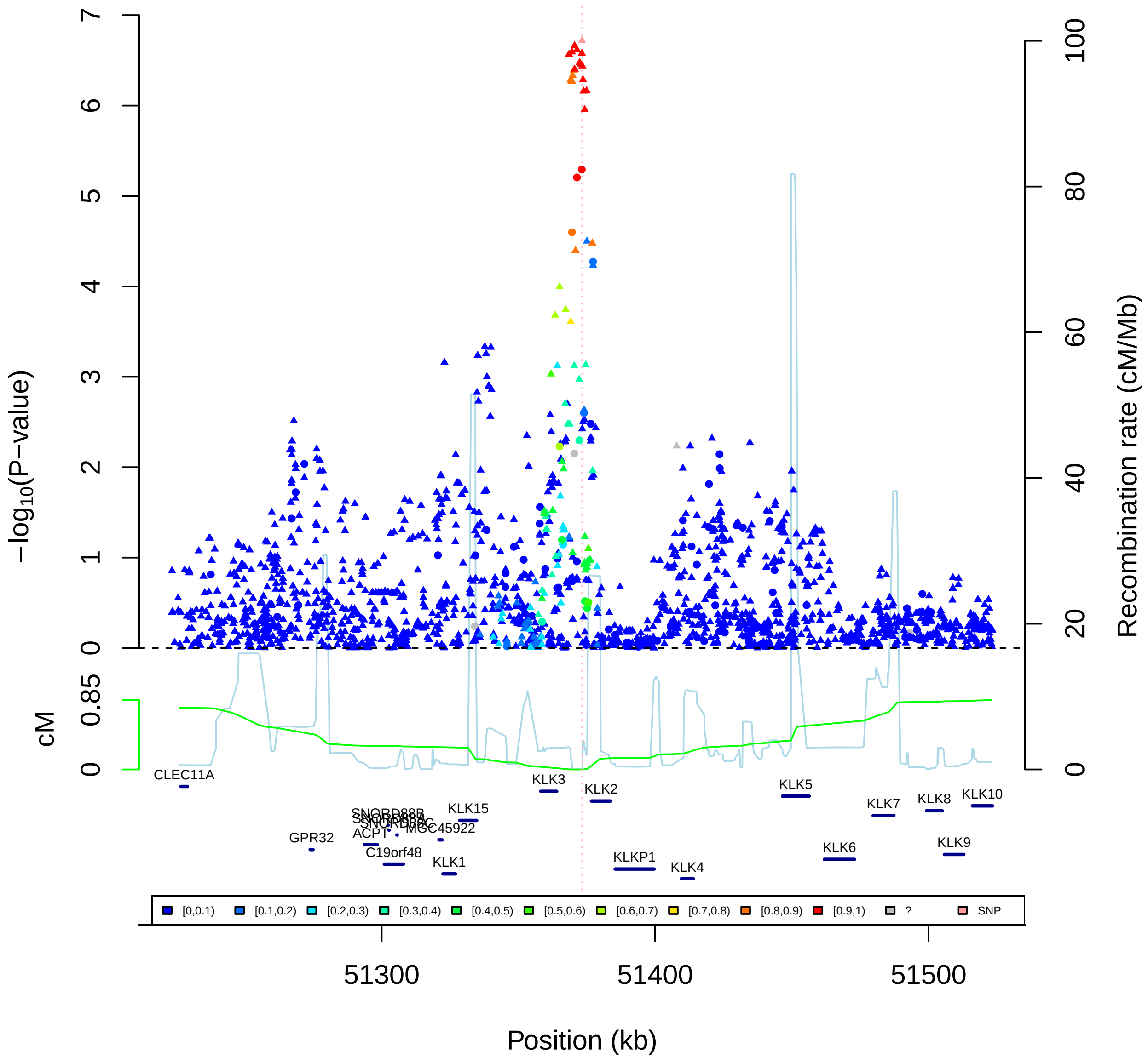

Supplementary Figure 3: Continued from previous page.

rs17632542

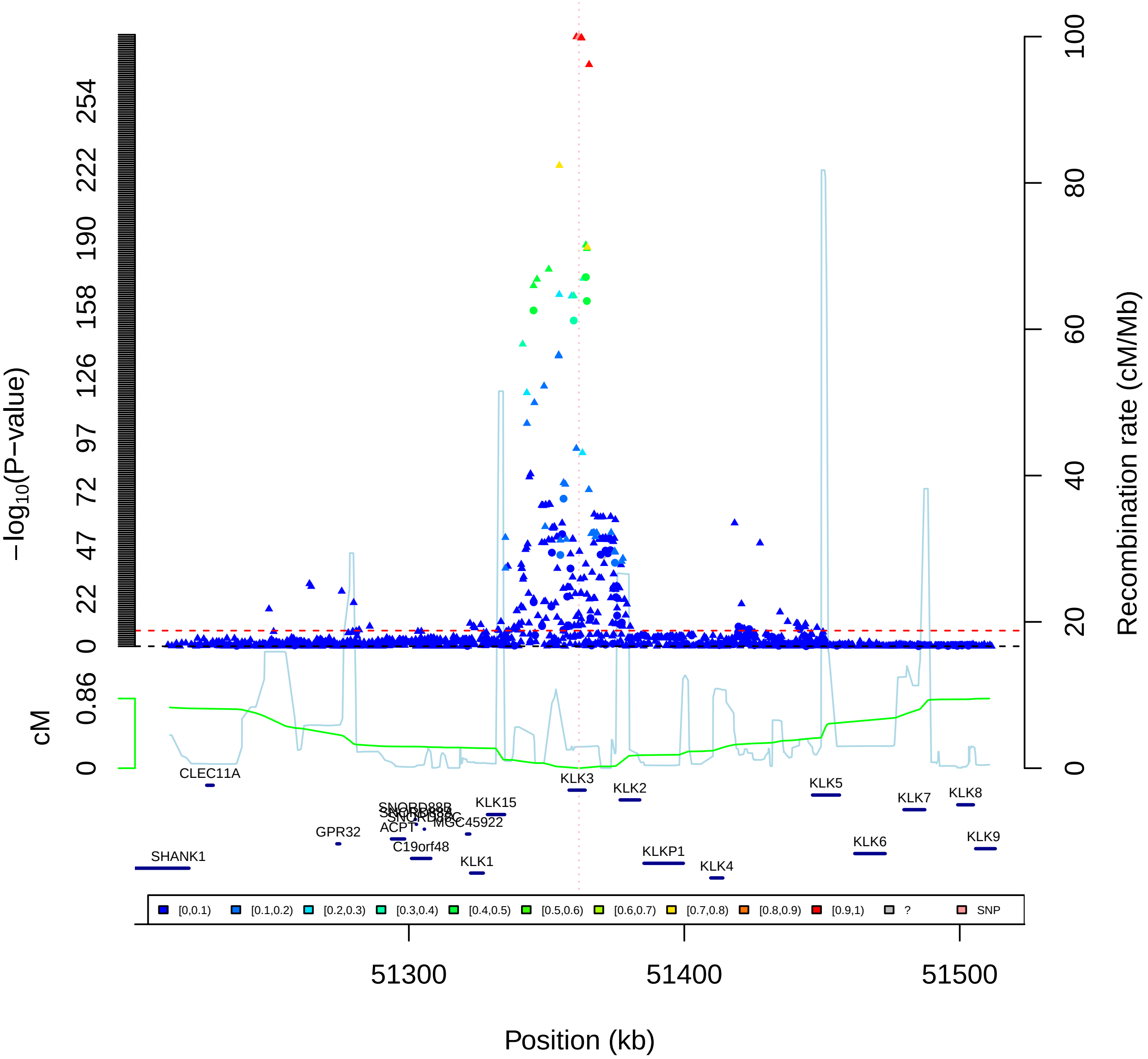

Supplementary Figure 3: Continued from previous page.

rs6070 (Conditional round 5)

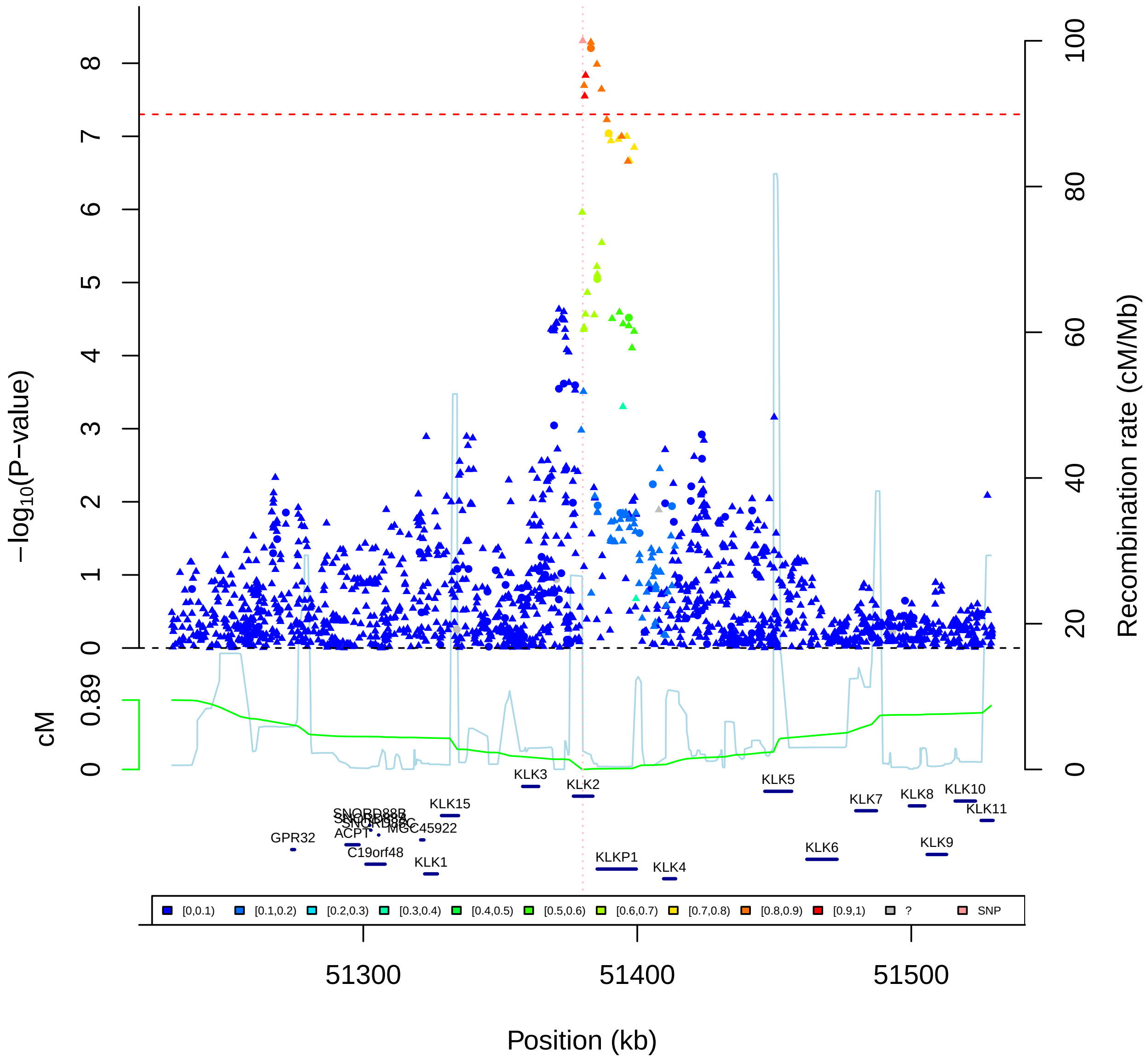

Supplementary Figure 3: Continued from previous page.

rs16980679

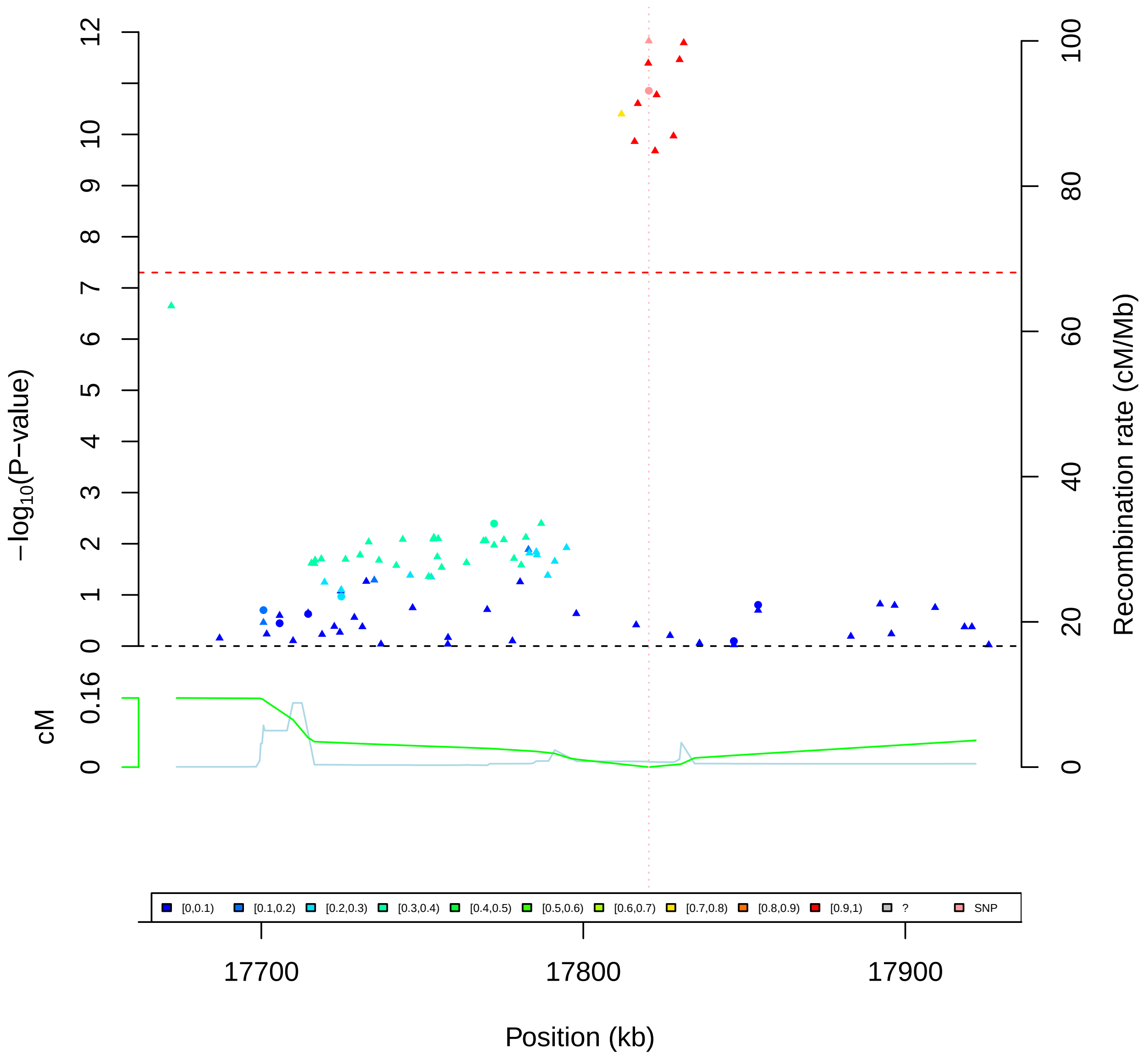

Supplementary Figure 3: Continued from previous page.

rs6627995

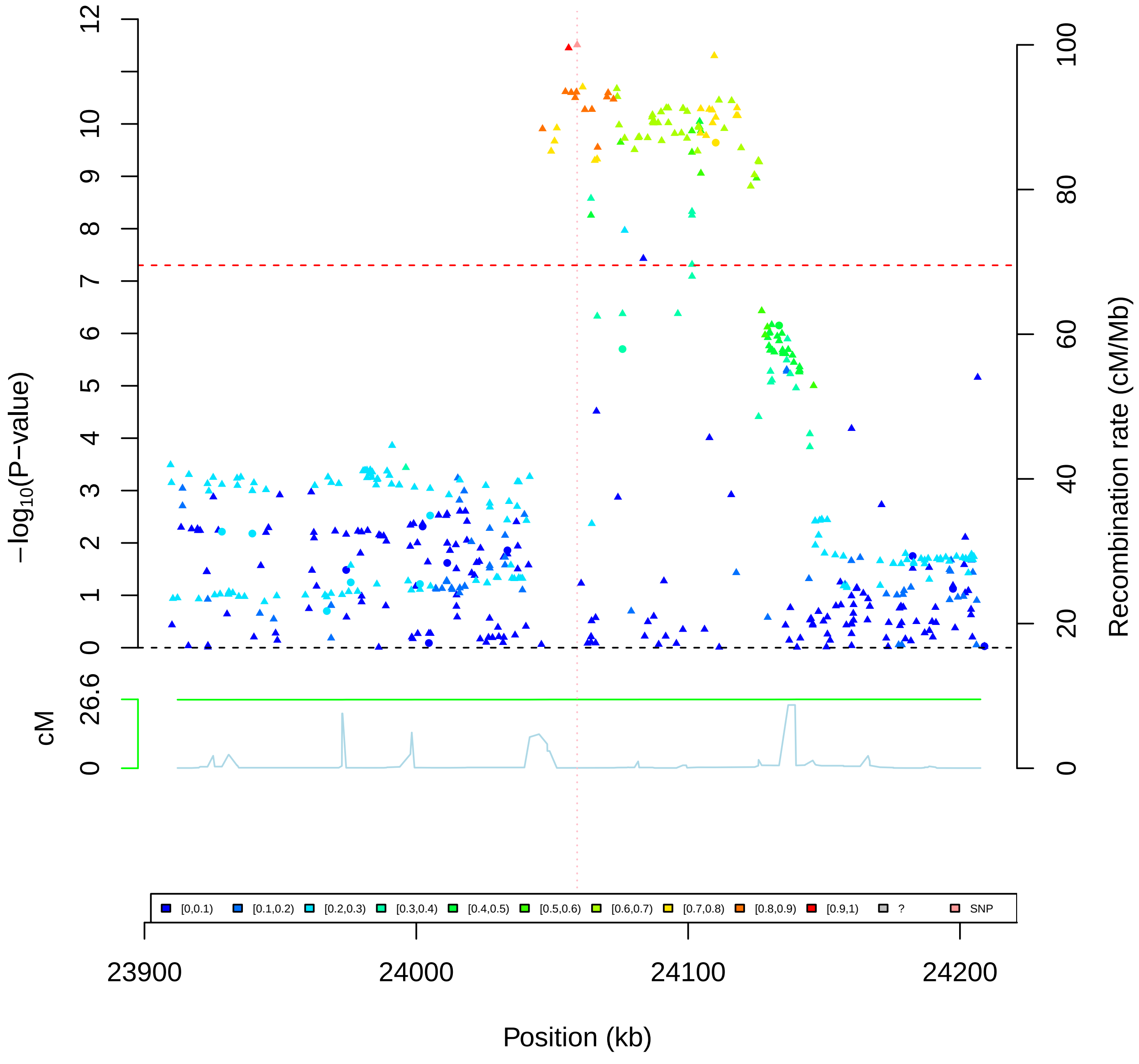

Supplementary Figure 3: Continued from previous page.

rs10855058

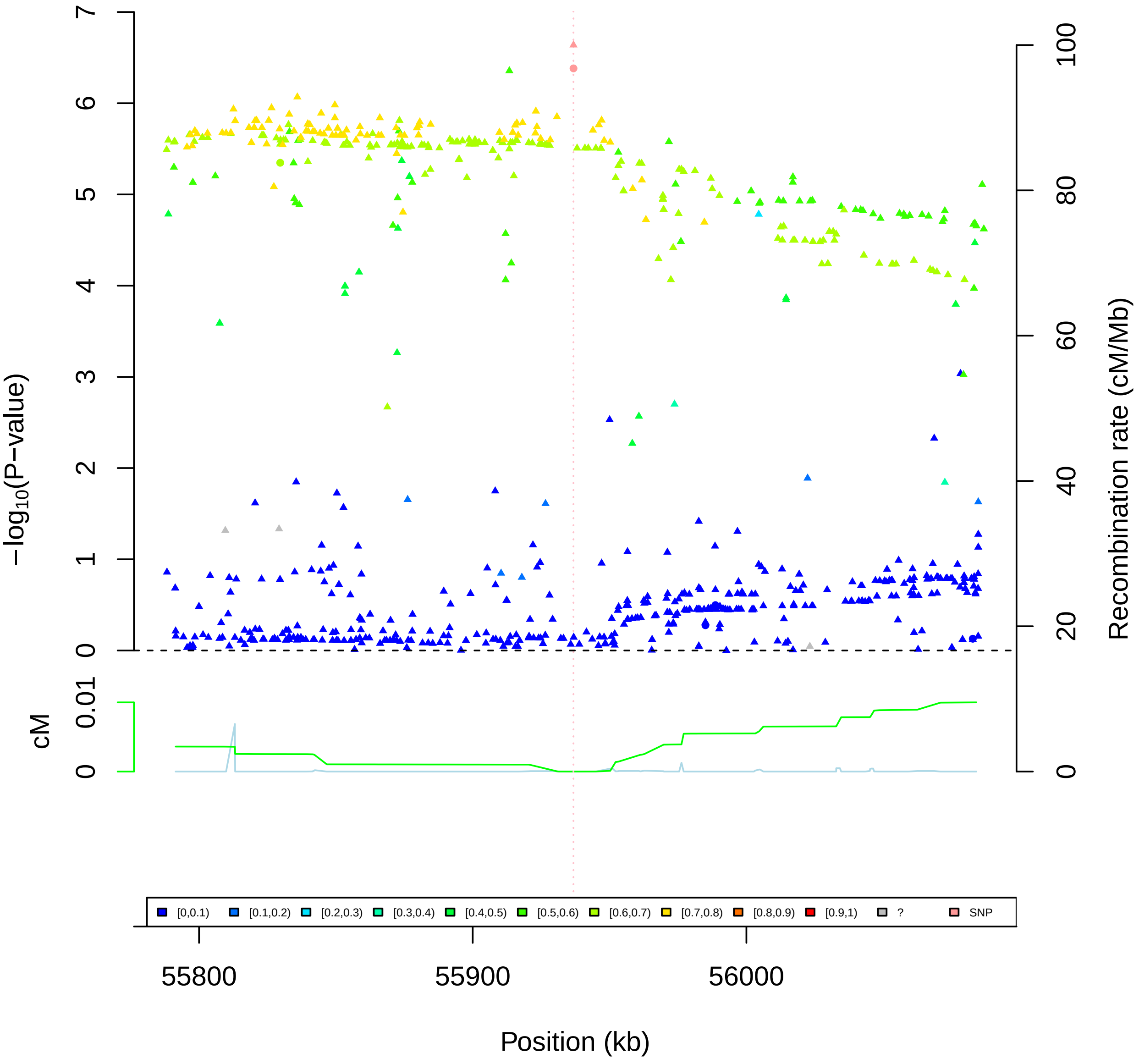

Supplementary Figure 3: Continued from previous page.

rs5969745 (Conditional round 1)

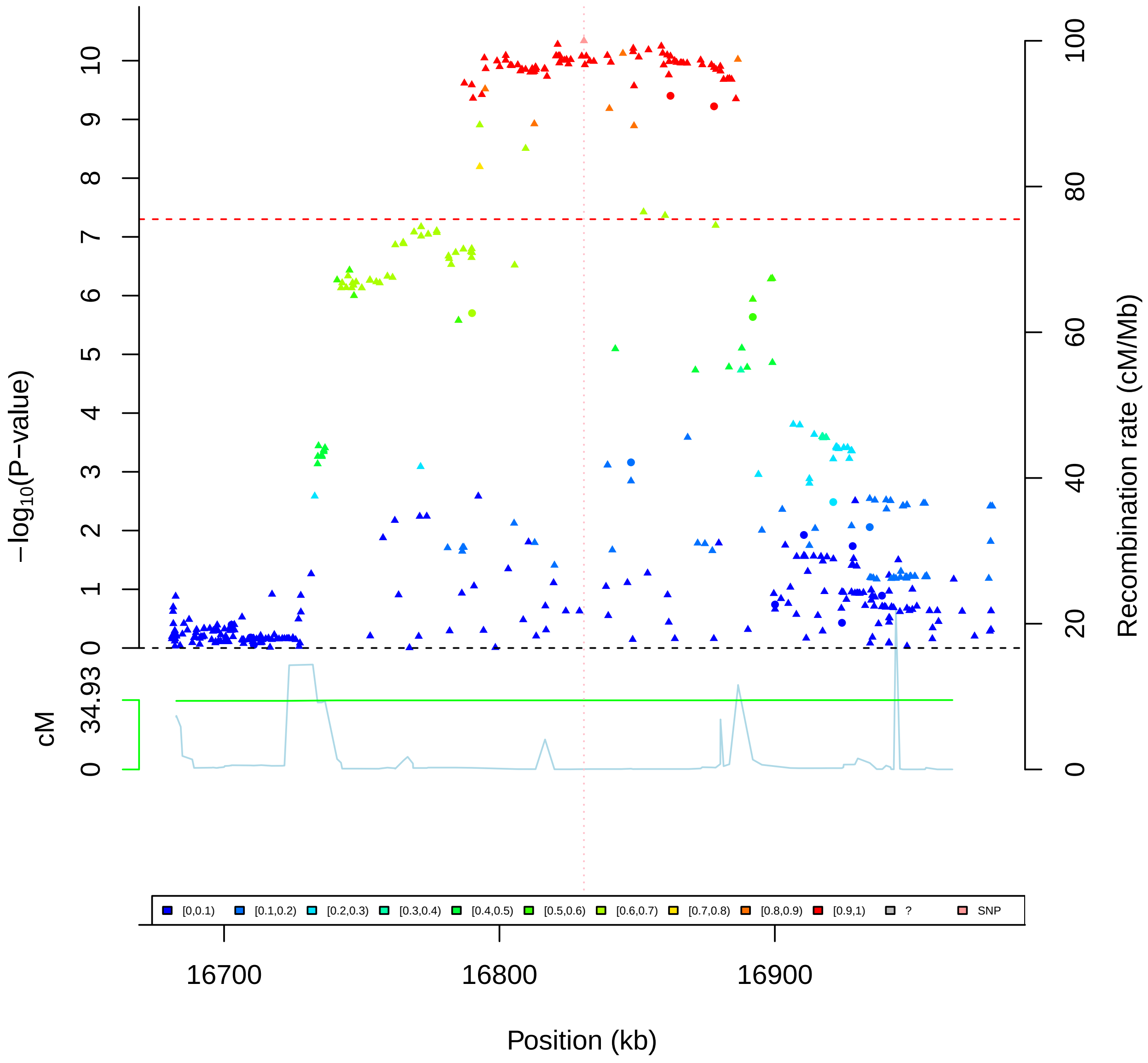

Supplementary Figure 3: Continued from previous page.

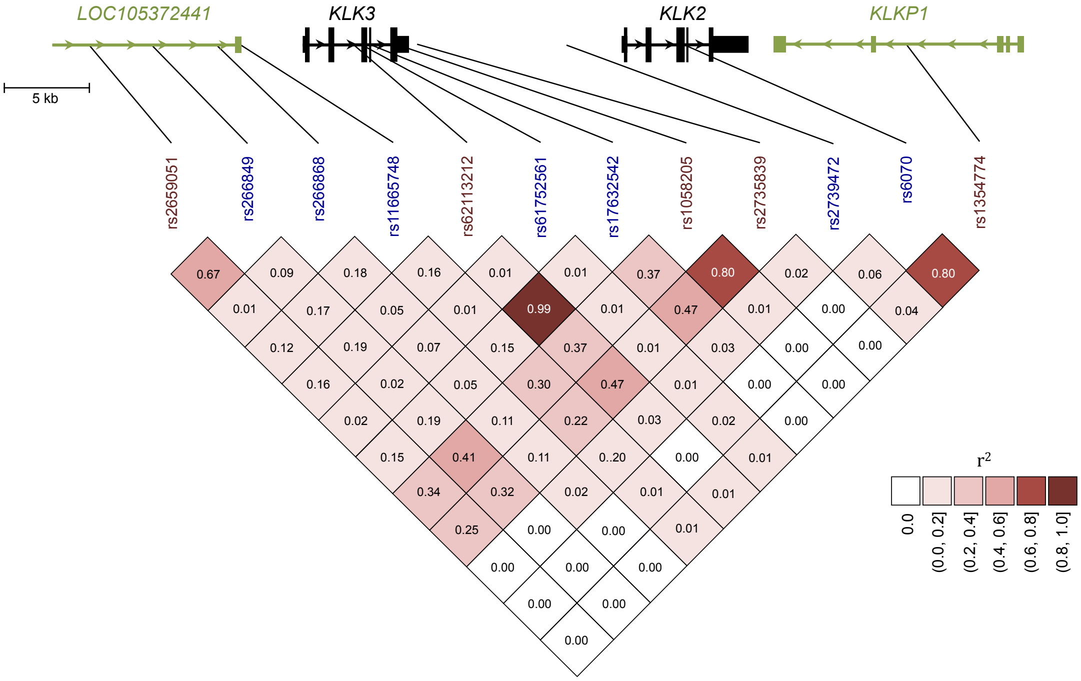

Supplementary Figure 4: Pairwise SNP correlations in 1000 Genomes European ancestry individuals in *KLK3* and *KLK2*. SNPs had p$< 5 \times 10^{-8}$ combined meta-analysis (blue) and from previous GWAS of PSA (red). Genomic positions based on GRCh37/hg19 assembly.
